# Clocking Epilepsies: A Chronomodulated Strategy-Based Therapy for Rhythmic Seizures

**DOI:** 10.3390/ijms24044223

**Published:** 2023-02-20

**Authors:** Sha Sun, Han Wang

**Affiliations:** 1 Center for Circadian Clocks, Soochow University, Suzhou 215123, China; 2 School of Biology and Basic Medical Sciences, Suzhou Medical College, Soochow University, Suzhou 215123, China

**Keywords:** epilepsy, epileptic genes, circadian clock, chronotherapy, animal models

## Abstract

Epilepsy is a neurological disorder characterized by hypersynchronous recurrent neuronal activities and seizures, as well as loss of muscular control and sometimes awareness. Clinically, seizures have been reported to display daily variations. Conversely, circadian misalignment and circadian clock gene variants contribute to epileptic pathogenesis. Elucidation of the genetic bases of epilepsy is of great importance because the genetic variability of the patients affects the efficacies of antiepileptic drugs (AEDs). For this narrative review, we compiled 661 epilepsy-related genes from the PHGKB and OMIM databases and classified them into 3 groups: driver genes, passenger genes, and undetermined genes. We discuss the potential roles of some epilepsy driver genes based on GO and KEGG analyses, the circadian rhythmicity of human and animal epilepsies, and the mutual effects between epilepsy and sleep. We review the advantages and challenges of rodents and zebrafish as animal models for epileptic studies. Finally, we posit chronomodulated strategy-based chronotherapy for rhythmic epilepsies, integrating several lines of investigation for unraveling circadian mechanisms underpinning epileptogenesis, chronopharmacokinetic and chronopharmacodynamic examinations of AEDs, as well as mathematical/computational modeling to help develop time-of-day-specific AED dosing schedules for rhythmic epilepsy patients.

## 1. Introduction

Epilepsy, resulting from neuronal hypersynchronous activities, is a severe neurological disorder that affects approximately 1% of the population worldwide [1]. The most disabling symptom of epilepsy is its unpredictability. The effective dosages of antiepileptic drugs (AEDs) vary among individual patients [2]; in particular, AED treatment efficacies for patients with the same epilepsy symptoms differ [3], and the prognoses among different types of epilepsy vary [4]. The unpredictability of epilepsy in individual patients may be due to genetic variation and heterogeneity [5]. Several reviews have discussed epilepsy pharmacogenomics or pharmacogenetics [6,7,8], which investigates different responses of individual patients with distinct genotypes to an AED and develops specific therapeutics for epilepsy patients based on their genotypes [8]. The effects of genes, for example, *ABCB1* (ATP-binding cassette subfamily B member 1, a drug transporter) [9], on epilepsy and interactions of *HLA-A* (major histocompatibility complex, class I, A) with adverse AED events [10] were investigated, highlighting the critical roles of genetic variation and heterogeneity in epilepsy treatment.

Although seizures are unpredictable, they are not random events. The circadian rhythms of epilepsy have been documented in earlier studies [11,12]. Findings in diagnostics using long-term EEG (electroencephalogram) recordings [13] and self-reported measures [14] further demonstrated the circadian patterns of epilepsies [15,16,17]. Generally, there are primarily diurnal and nocturnal types of epilepsies [18]. The sleep–wake cycle is also a non-negligible factor [19]. In addition, different epileptogenic regions display daily occurrences in various temporal epilepsy syndromes [20].

Chronotherapy emphasizes that time-of-day-specific treatment is critical for maximizing therapeutic efficacies and minimizing side effects [21]. In epilepsy, differential dosing of AEDs at the circadian-modulated seizure peak is an effective means of chronotherapy [22]. Evidence has demonstrated that differentially dosing AEDs improves seizure control and prevents drug resistance [23,24]. Hence, the prediction of time-dependent drugs and prevention of potential toxicity, based on chronotherapy testing, should help improve the efficacies of AEDs. Despite well-characterized circadian patterns, little is known about circadian roles in seizures and chronotherapeutics for epilepsy.

In this review, we compiled as many as 661 epilepsy-related genes from 2 public databases, focused on 192 epileptic driver genes as a cause of epilepsy, and then divided them into 20 KEGG categories. The molecular circadian system and the circadian rhythms of different human and animal seizures are discussed. Studies that link circadian clock genes and epilepsy are then reviewed, highlighting the role of the circadian clock in epilepsy diagnosis and therapy. The development of animal models for epilepsy studies is summarized. Finally, we emphasize targeting the circadian clock and circadian clock-regulated epileptic processes as a chronomodulated strategy for epilepsy therapy. Highlighting the roles of circadian clock genes and the circadian clock in epilepsy provides novel targets for developing AEDs and effective chronotherapy for the large proportion of rhythmic epilepsy patients.

## 2. Epilepsy Genes

Even though state-of-the-art medical management with a number of AEDs has been well developed in recent decades, approximately 40% of epilepsy patients treated with an AED fail to reach long-term remission and instead develop drug-resistant epilepsy (DRE) with frequent seizures and increased mortality [25,26]. In a long-term study of 144 childhood-onset epilepsy patients, only 23 (16%) patients were cured after 1 year of treatment without a relapse for almost 40 years, 46 (32%) patients took an average of 8.5 years of treatment to reach terminal remission, and 28 (19%) patients followed numerous years of a remitting–relapsing course to be cured; whereas 20 (14%) patients displayed remission but failed to reach terminal remission after relapse, and 27 (19%) patients never exhibited remission and were regarded as DRE [27]. In other words, approximately one-third of epilepsy patients eventually developed drug-resistant or refractory epilepsy. Among several hypotheses postulated for unraveling the pathogenesis of DRE, the “genetic hypothesis” assumes that single-nucleotide polymorphism (SNP) variants underpin the differential susceptibility to drug resistance in epilepsy patients [28,29]. Pharmacogenetics refers to designing a specific treatment for a patient based on his/her genotype [30]. Therefore, genetic variations affect the efficacy of drugs, and genetic testing has become necessary for clinical research [31]. Studies from twins have shown that the rate of generalized epilepsy and focal epilepsy is higher in monozygotic twins than in dizygotic twins [32,33]. The first epilepsy gene, *CHRNA4* (cholinergic receptor nicotinic alpha 4 subunit), one of the genes responsible for focal epilepsy, was reported in 1995 [34]. *CHRNA4* belongs to the ligand-gated ion channel family [35], and its mutations cause nocturnal frontal-lobe epilepsy [36]. These studies underscore the importance of genes and genetics for epilepsy diagnosis and treatment [37].

### 2.1. Compilation of 661 Epilepsy-Related Genes from 2 Public Databases

**Figure 1:**
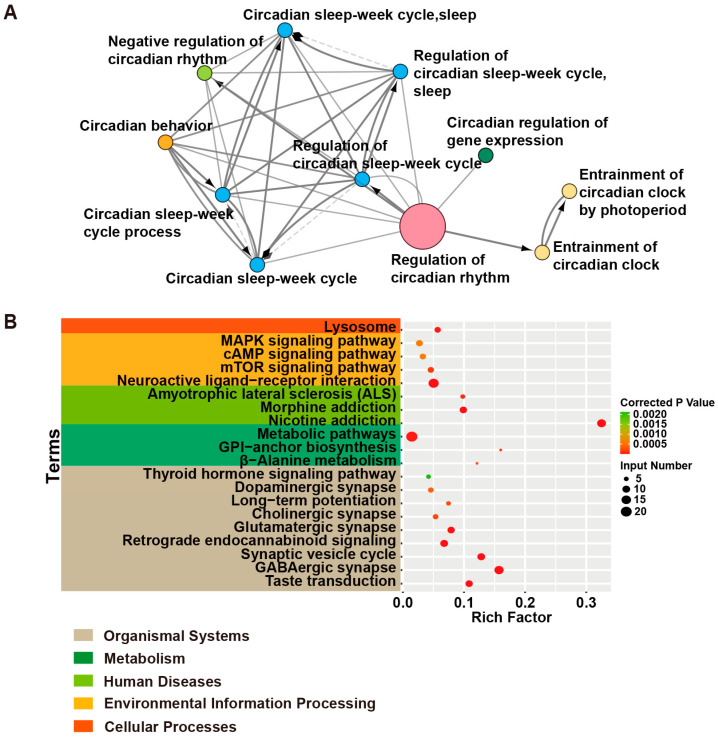
GO and KEGG analyses of human epilepsy-related genes. (**A**) The circadian rhythm pathway is enriched in the GO analysis of human epilepsy-related genes. These 661 epilepsy-related genes were classified into biological processes with ClueGO in Cytoscape. Those with Kappa scores higher than 0.4 are presented. Those with the circadian term are shown. The node size indicates the number of genes in each pathway, the lines between the nodes indicate the correlations between the terms, and dotted lines possible correlations between the terms. The same color in different nodes indicates the same sets of genes enriched in different pathways. The arrows indicate the affiliation between pathways. (**B**) The top 20 ranked KEGG pathways of the 192 driver genes are shown. Pathways with the same category were sorted and merged.

Next-generation sequencing (NGS) has facilitated the advancement of human genetics and genomics over the last decade [38]. In 2014, the Epilepsy Genetics Initiative (EGI) was established to help discover novel epilepsy genes and to identify new molecular diagnoses [39]. The Public Health Genomics and Precision Health Knowledge Base (PHGKB, https://phgkb.cdc.gov/, accessed on 9 March 2021) has compiled disease genes from published studies. Approximately 526 genes associated with epilepsy have been listed in the PHGKB—for instance, *SCN1A* (sodium voltage-gated channel alpha subunit 1) as a drug target gene [40,41,42] and *CYP2C9* (cytochrome P450 family 2 subfamily C member 9), which is involved in drug metabolism [43,44], have been examined in epilepsy studies. Furthermore, the Online Mendelian Inheritance in Man (OMIM, https://www.omim.org/, accessed on 9 March, 2021) database also catalogs epilepsy-related genes, listing 194 epileptic-associated genes. We compared epilepsy-related genes from PHGKB and OMIM and obtained a total of 661 epilepsy-related genes (overlapping genes + nonoverlapping genes of the 2 databases; Supplementary Table S1), which is comparable with previous efforts to assemble epilepsy-related genes [45,46]. Interestingly, the circadian clock pathway was enriched in GO analysis, and several circadian clock genes, such as *PER2* and *PER3,* were regarded as epilepsy-related genes (Figure 1A, Supplementary Table S2), indicative of circadian involvement in epilepsies/seizures.

### 2.2. Epileptic Driver Genes, Passenger Genes, and Undetermined Genes

Through interrogating the published relevant studies, these 661 genes were then divided into 3 groups: 192 driver genes, 176 passenger genes, and 293 undetermined genes (Supplementary Table S1). A driver gene is defined as one whose mutation or loss causes seizures. In contrast, the passenger gene is defined as one that exhibits altered expression during seizures or causes neurological diseases accompanying epilepsy. We classified the remaining genes into undetermined genes, which do not yet play a clear role in epilepsy, including those involved in drug metabolism. For example, protein SZT2 (seizure threshold 2) deficiency resulted in hyperactivation of mTORC1 (mechanistic/mammalian target of rapamycin complex 1) signaling in HEK293 and HeLa cells, leading to neonatal death in mice [47], and the homozygous mutation of its gene is responsible for epilepsy in a Saudi family [48]. Therefore, we classify this gene as an epilepsy driver gene. Even though the mutations of some genes were revealed in epileptic patients, whether these genetic mutations are responsible for epilepsy is unclear. For instance, *GNAO1* (G protein subunit alpha o1), whose loss-of-function (LOF) mutations are associated with epileptic encephalopathy but whose gain-of-function (GOF) and synonymous mutations are associated with patients with movement disorders that may not display epilepsy [49] or *SLC7A6OS* (solute carrier family 7 member 6 opposite strand), has recently been found in two unrelated progressive myoclonus epilepsy families, but its role in epilepsy is unknown [50]. Hence, the two genes are regarded as epilepsy passenger genes. Furthermore, some genes were associated with epilepsy, such as *SLC22A1* (solute carrier family 22 member 1), a cation transporter gene essential for removing environmental toxins and drugs [51]. *SLC22A1* was shown to contribute to altered lamotrigine (LTG) plasma concentrations during AED treatment [52], but no *SLC22A1* variants have been shown to cause or affect epilepsy to date; thus, we regard it as an undetermined gene.

We then employed KOBAS (KEGG Orthology-Based Annotation System, http://kobas.cbi.pku.edu.cn/kobas3, accessed on 8 August 2022) to classify these 192 epilepsy driver genes into several clusters based on biological processes (Table 1). The top 20 ranked pathways were sorted and merged according to upper-level categories (Figure 1B). We then focus on discussing the epileptic driver genes involved in ion channels, the mTOR pathway, synaptic support proteins, and transcription regulation, as well as the effects of some epileptic genes on circadian rhythms and the sleep–wake cycle [37], as many of the same pathways are also enriched in epileptic passenger genes.

#### 2.2.1. Ion Channel Genes

Not surprisingly, numerous nervous-system-related processes/pathways are enriched in these epilepsy driver genes, and we first focus on genes involved in the cholinergic and GABAergic synapses. As described above, *CHRNA4* involved in the cholinergic synapse was the first identified epilepsy gene causing autosomal dominant nocturnal frontal-lobe epilepsy (ADFLE), which occurs mainly during NREM (non-rapid eye movement) sleep [34]. Ever since, *CHRNA2* and *CHRNB2*, as well as an additional *CHRNA4* mutation, have been reported in sleep-related FLE (frontal lobe epilepsy) [53,54,55] (Figure 1B). The 3 genes *CHRNA2*, *CHRNA4*, and *CHRNB2* encode the α2, α4, and β2 subunits of the nicotinic acetylcholine receptor (nAChR), respectively, which, as members of a family of ligand-gated ion channels, form cation-selective ion channels for sodium and potassium in response to ligands such as nicotine and acetylcholine [56]. Although the 3 subunits α2, α4, and β2 are all highly expressed in the cerebral cortex of monkeys [57,58], only α2 is highly expressed in GABAergic cells of the deep layers in rodents [59,60,61]. A *CHRNB2* mutation (V287M) near the extracellular end of the M2 (second transmembrane) domain of the nAChR β2 subunit likely destroys the walls of the ion channel and was revealed in a Scottish family [54], indicating that the structural integrity of ion channels/receptors is important for resisting seizures. Furthermore, GABAergic synapses have been reported to contribute to seizure regulation (Figure 1B, Table 1) [62]. As one of the first targets of epilepsy gene therapy, increasing GABA levels in the epileptogenic area elevates the threshold of neuronal excitability, which reduces seizures [63]. GAD (glutamic acid decarboxylase), the catalytic enzyme of GABA biosynthesis, has been used to enhance GABA levels [64]. *GAD1* encodes a 67 KD molecular weight protein, i.e., GAD67, which produces up to 90% of the GABA in the CNS [65,66]. *GAD1* mutations are associated with neurodegenerative diseases such as schizophrenia, bipolar disorder, and other movement disorders [67,68], and *Gad67*^−/−^ knockout mice display neonatal death [65]. A recent study found that biallelic *GAD1* mutation was associated with seizures and reduced muscle tone in six unrelated families [69]. Animal studies have also shown that *GAD1* mutations result in reduced GABA synthesis and induce seizures that reduce GABA release and, in turn, cause imbalanced brain activity [70]. In addition, GABA receptors mediate the activity of downstream neurons. GABA_(A)_ receptors possess 18 subunits, including α(1–6), β(1–3), γ(1–3), δ, ε(1–3), θ, and π, and they mediate fast inhibitory actions in the brain [71,72]. While malfunction of GABA_(A)_ receptors (such as α1) has been shown to contribute to human epilepsy disorders [73], downregulation of synaptic GABA_(A)_ receptors, as well as the reduced phosphorylation of the β3 subunit, was observed in lithium-pilocarpine-induced status epilepticus rats [74,75]. However, upregulation of the GABA_(A)_ receptors has been observed in the dentate gyrus of temporal lobe epilepsy (TLE) mice [76]. Thus, GABA_(A)_ receptors have become a therapeutic target of epilepsy.

In addition to the ligand-gated ion channels, the voltage-gated ion channels, including sodium (Na^+^), potassium (K^+^), and calcium (Ca^2+^) channels [77], are also involved in epilepsy. Genetic variants in *SCN1A*, *SCN2A*, and *SCN1B*, encoding voltage-gated sodium channels, contributed to early-onset epilepsy [78]. Missense mutations or deletion of *SCN1A* have been shown to lead to loss of sodium current in GABA-mediated inhibitory interneurons, resulting in Dravet syndrome (DS), which often occurs in the first year of life) [79,80]. In contrast, gain-of-function *SCN2A* missense mutations have recently been reported to be associated with early infantile seizures, such as developmental and epileptic encephalopathy (DEE), which usually occur as early as three months of life) [78,81]. The patient with early infantile seizures was also shown to harbor the *SCN1B p.C121W* mutation [82], and mice engineered to carry the *SCN1B p.C121W* mutation display reduced dendrites of pyramidal neurons and hyperexcitability of the specific brain region [83]. Benign familial neonatal seizures (BFNS) as another early-onset epilepsy have been revealed to be associated with loss-of-function mutations of voltage-gated potassium channel genes, including *KCNQ2* (potassium voltage-gated channel subfamily Q member 2) and *KCNQ3* [84]. KCNQ2 and KCNQ3 are expressed in the whole brain region, form homo- and heterotetrameric channels, and produce the M-current important for controlling membrane potentials [85]. Whereas homozygous *Kcnq2*^−/−^ knockout mice die after birth due to pulmonary atelectasis, heterozygous *Kcnq2^+/^*^−^ mice are viable and sensitive to pentylenetetrazole (PTZ) [86]. *CACNA1A* (Ca_v_2.1 α1 subunit) encodes P/Q type channels, whose mutations are associated with absence seizures [87]. Thus, various mutations of ion channel genes have been shown to result in epilepsies [88].

#### 2.2.2. Genes Involved in the mTOR Pathway

In recent years, inhibition of the mTOR pathway has become a new therapeutic strategy in epilepsy [89,90] because the mTOR pathway is associated with malformations of cortical development (MCD) coupled with intractable epilepsy [91]. *DEPDC5* (DEP domain containing 5), *NPRL2* (nitrogen permease regulator-like 2), and *NPRL3* (nitrogen permease regulator-like 3) are 3 components of the GATOR1 (GTPase-activating protein (GAP) activity toward Rags 1) protein complex, and their loss-of-function mutations have been identified in MCD-associated epilepsies [92,93,94]. In particular, these mutations lead to the upregulation of mTORC1, thereby contributing to focal epilepsy [95].

#### 2.2.3. Genes Encoding Synaptic Support Proteins

The synaptic vesicle cycle is also included in the top 20 KEGG pathways in our analysis (Figure 1B). We will discuss several genes, such as *DNM1*, *STX1B,* and *STXBP1*. *DNM1* (dynamin 1) encodes a GTP-binding protein involved in synaptic vesicle fission on the presynaptic membrane [96]. Mutations in the GTPase and the middle domains of DNM1 result in impaired endocytosis of synaptic vesicles, leading to severe seizures with intellectual disability and hypotonia [97]. *STX1B* (syntaxin 1b) and *STXBP1* (syntaxin-binding protein 1) play a role in exocytosis; specifically, STX1B mainly mediates releasing the Ca^2+^-dependent synaptic vesicle, whereas STXBP1 secures the correct position of syntaxin-1 [98]. *STX1B* mutations result in tonic–clonic seizures, absence seizures, and myoclonic seizures [99]. *STX1B* and *STXBP1* mutations are often found in infant seizures [98].

#### 2.2.4. Transcriptional Regulators

Several epileptic driver genes involved in transcription have also been studied. The *ARX* (Aristaless-related homeobox) encodes a transcription factor important for neuronal development [100], whose loss-of-function variants/mutations contribute to X-linked intellectual disability and epilepsy [101,102], while increasing the gene copy number of the other X-linked gene *MECP2* (methyl-CpG binding protein 2) leads to MECP2 duplication syndrome (MDS) [103]. Up to 90% of children with MDS have been shown to have seizures during adolescence [104]. These studies suggest that transcription regulation also contributes to epileptogenesis.

#### 2.2.5. Effects of Epileptic Genes on Circadian Rhythms and the Sleep–Wake Cycle

Several epileptic driver genes have been known to affect circadian rhythms and the sleep–wake cycle. *Scn1a^+/^*^−^ DS mice display impaired sleep, characteristic of an extended sleep period and fragmented non-rapid eye movement (NREM) sleep [105], whereas *Scn2a*^−/−^ mice result in reduced NREM sleep and increased wakefulness, accompanied by a disrupted spontaneous firing pattern in SCN and altered expression of core circadian clock genes [106]. Epileptic *Kcna1*^−/−^ mice exhibit a lengthened circadian period with an extended wake period and reduced sleep time, as well as damped oscillations of core circadian clock genes such as *Clock*, *Bmal1*, and *Per1* [107]. Similarly, loss of *Drosophila cac* (*cacophony*) (an ortholog of CACNA1A) also results in reduced sleep time [108]. Furthermore, *MECP2* is likely regulated by the circadian clock, and its disrupted expression is expected to be responsible for sleep disorders during pathological stages [109]. Therefore, identifying these circadian-clock-related epileptic genes and further elucidating their functions should shed light on the reciprocal effects between epileptogenesis and the circadian clock, provide novel targets for drug development, and contribute to precise epilepsy treatment.

## 3. Circadian Rhythms in Human Epilepsies

As early as 1885, William R. Gowers observed three groups of epilepsy patients in daily patterns: diurnal, nocturnal, and diffuse [12]. Diurnal seizures occur at certain times of the day, whereas nocturnal seizures tend to occur primarily at bedtime and at night [110]. Recently, SeizureTracker (Springfield, VA, USA) and NeuroVista (Melbourne, VIC, Australia) were employed to analyze the rhythmic patterns of seizures and found that the seizure rates of approximately 80% of 1118 patients displayed daily variations [111]. The mechanisms underpinning why seizures display daily variation are not clear. A legitimate hypothesis is that the circadian clock contributes to seizure rhythmicity. However, little is known about the circadian roles in epilepsy and seizures.

**Figure 2:**
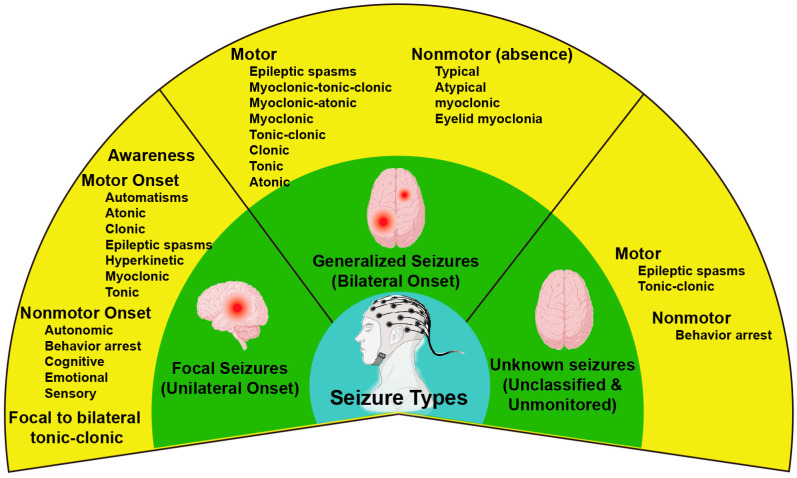
The classification of seizure types proposed by the International League Against Epilepsy 2017. Focal seizures occur within one hemisphere, whereas generalized seizures occur in the bilateral hemisphere. The onset of some seizures is unknown, which may not be classified as focal or generalized seizures. A further classification of seizures highlights features of seizure onset, including awareness level, motor onset, or nonmotor onset symptoms. Drawn from Fisher et al., 2017 [115].

The International League Against Epilepsy (ILAE) broadly categorizes seizures into focal seizures, generalized seizures, and seizures of unknown onset [112,113,114]. Focal seizures occur only in discrete brain regions limited to one hemisphere, generalized seizures involve large bilateral brain areas, even the whole brain cortex, and seizures of unknown onset do not belong to the focal or generalized categories (Figure 2) [112,114,115]. EEG recordings of generalized seizures have been revealed to be significantly more robust in the morning than in the afternoon [116]. Furthermore, focal seizures have been shown to manifest a predictable daily pattern (Table 2) [16]. Parietal lobe epilepsy (PLE) occurs primarily around the end of sleep in the morning [15,20]. Two peaks of PLE were found in Hofstra’s study of 450 times of seizures: one peaking around 05:00 to 11:00, and the other peaking around 17:00–23:00 [117]. In contrast, occipital and temporal lobe epilepsy often occurs in the afternoon [18,20,118]. Temporal lobe epilepsy has been classified into mesial (MTLE), lesional (LTLE), and neocortical temporal lobe (NTLE) epilepsy. MTLE displays two peaks, 07:00–08:00 and 16:00–17:00 [20,117,119,120], respectively, whereas LTLE peaks around 11:00, and NTLE peaks around 11:00–17:00 [117] and early morning [120]. On the other hand, interictal epileptiform discharges (IEDs), sharp waves in the EEG background between seizures, show a nocturnal predominance and often occur during NREM sleep [16,121,122]. However, recent studies have shown that the peak of nocturnal predominance in interictal epileptiform activity (IEA) was independent of the region of seizure irritability, monitored by an implantable brain stimulator (RNS^®^ system, Neuropace, Mountain View, CA, USA) continuously for a more extended period [120,123]. Together, these studies indicated that the circadian rhythm of seizures was robust and endogenous, independent of antiseizure dosing [111,124], indicating a possible circadian role in epileptic pathogenesis.

However, relatively little is known about disrupted circadian rhythms in epilepsy patients. Analysis of the sleep–wake cycle of 20 patients with TLE and 20 patients with juvenile myoclonic epilepsy (JME) showed that JME patients tend to sleep later at night and get up later in the morning, whereas TLE patients are morning-types [19]. Twenty-four-hour EEG (electrocardiography) recording of the interictal circadian rhythm of heart rate (HR) variability revealed no nocturnal increase in heart rate variability in TLE patients, highlighting the attenuated circadian heart rate rhythm in TLE patients [125]. Furthermore, epileptic patients produced significantly elevated melatonin levels during the nighttime with an altered phase, even though they maintained melatonin secretion rhythms [126]. Hence, elucidation of the mechanisms underlying circadian roles in epileptic pathogenesis is critically important for providing novel targets for AED development and developing a novel chronomodulated strategy-based treatment for these rhythmic epilepsy patients.

**Table 2:** Circadian rhythms of human epilepsies.

Seizures	Peak in 24-h Cycle	Subjects No. (Seizure No.)	References
**TLE**	11:00–17:00 11:00–19:00 11:00–15:00	176 (808) 26 (90) 1 (694)	Hofstra et al. (2009) [118] Pavlova et al. (2004) [18] Quigg et al. (2000) [15]
**LTLE**	Morning	8 (48)	Quigg et al. (1998) [127]
**MTLE**	05:00–11:00 and 11:00–17:00 15:00 07:00–10:00 and 16:00–19:00 06:00–08:00 and 15:00–17:00 03:00 and 17:00–20:00	33 (450) 64 (774) 131 (669) 60 (694) 72 (No mention)	Hofstra et al. (2009) [117] Quigg et al. (1998) [127] Durazzo et al. (2008) [20] Karafin et al. (2010) [119] Spencer et al. (2016) [120]
**NTLE**	11:00–17:00 03:00–07:00	33 (450) 18 (No mention)	Hofstra et al. (2009) [117] Spencer et al. (2016) [120]
**XTLE**	Morning	26 (465)	Quigg et al. (1998) [127]
**FLE**	23:00–05:00 19:00–23:00 04:00–07:00 around 03:00	33 (450) 26 (90) 131 (669) 17 (No mention)	Hofstra et al. (2009) [117] Pavlova et al. (2004) [18] Durazzo et al. (2008) [20] Spencer et al. (2016) [120]
**PLE**	05:00–11:00 and 17:00–23:00 04:00–07:00 01:00–06:00	33 (450) 131 (669) 1 (315)	Hofstra et al. (2009) [117] Durazzo et al. (2008) [20] Quigg et al. (2000) [15]
**OLE**	19:00–23:00 16:00–19:00	26 (90) 131 (669)	Pavlova et al. (2004) [18] Durazzo et al. (2008) [20]
**GED**	Morning	29 (No mention)	Labate et al. (2007) [116]

MTLE = mesial temporal lobe epilepsy; XTLE = extratemporal lobe epilepsy; LTLE = lesional temporal lobe epilepsy; NTLE = neocortical temporal lobe epilepsy; FLE = frontal lobe epilepsy; PLE = parietal lobe epilepsy; OLE = occipital lobe epilepsy; GED = generalized epileptiform discharge.

## 4. The Circadian Clock

Circadian rhythms, as biological rhythms with a period of approximately 24 h, are regulated and controlled by an endogenous time-keeping mechanism, i.e., the circadian clock [128,129,130,131]. In mammals, the central clock is situated at the suprachiasmatic nuclei (SCN) of the anterior hypothalamus [132]. The mammalian SCN neurons exhibit higher activity in electrical physiology and metabolism during the daytime [133,134]. External light is received by intrinsically photosensitive retinal ganglion cells (ipRGCs) and transmitted to the SCN via the retinohypothalamic tract (RHT) [135]. The efferent from the SCN projects to the pineal gland and drives the rhythmic release of melatonin, a sleep-promoting hormone. Since light is known to inhibit melatonin synthesis, the daily oscillation of melatonin is similar in both diurnal and nocturnal animals [136,137] and helps to synchronize peripheral organs in the body [138]. Intriguingly, most organs, tissues, and cells display circadian rhythmicity, regulated by the local peripheral clock, as well as neural, hormonal, and metabolic cues from the SCN [139].

**Figure 3:**
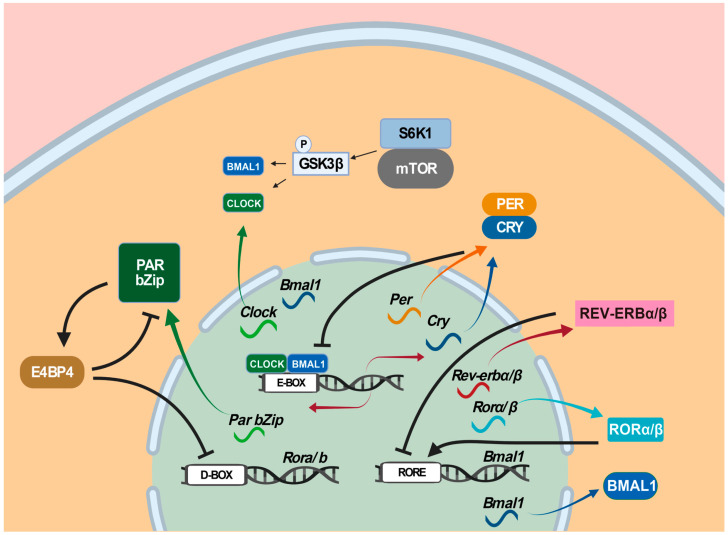
Mammalian circadian clockwork model. Three transcription-translation feedback loops are known to operate in the mammalian circadian clock. In the first loop, the CLOCK: BMAL1 heterodimer activates the expression of target genes, including Per genes (Per1, Per2, and Per3) and Cry genes (Cry1 and Cry2) via binding to E-box (5′-CACGTG-3′) in their promoter regions, whereas the PER: CRY heterodimer interferes with the transcriptional activity of the CLOCK-BMAL1 heterodimer and turns off their own expression. In the second loop, Rorα/β and Rev-erbα/β are regulated by CLOCK and BMAL1 via E-box, whereas their proteins RORα/β and REV-ERBα/β activate and suppress Bmal1 by competing for binding to the RORE (retinoic-acid-related orphan receptor response element). In the third loop, Dbp, Hlf, Tef, and E4bp4/Nfil3 are all regulated by CLOCK and BMAL1 via E-box, whereas their proteins DBP, HIF, TEF, and E4BP4/NFIL3 bind to D-box in the promoter regions of their target genes, where DBP, HIF, and TEF activate D-box-containing genes and E4BP4/NFIL3 represses them. Among these three circadian clock-controlled cis-elements-mediated transcriptional feedback loops, the E/E’-box-mediated loop plays the dominant role in the circadian clock. In addition, the mTOR pathway has been shown to contribute to circadian regulation. Color arrows indicate transcription or translation, black arrows transcription activation, and turnstile symbol suppression.

Three transcription-translation feedback loops as molecular time-keeping mechanisms are known to generate, regulate, and maintain circadian rhythms [140,141]. In the primary loop, the CLOCK: BMAL1 heterodimer as the positive limb activates the expression of target genes, including *Per* genes (*Per1*, *Per2,* and *Per3* ) and *Cry* genes (*Cry1* and *Cry2*) via binding to E-box (5′-CACGTG-3′) and E’-box (5′-CACGTT-3′) in their promoter regions [142], whereas the PER: CRY heterodimer as the negative limb interferes with the transcriptional activity of the CLOCK-BMAL1 heterodimer and turns off their expression [143]. In the second loop, *Rorα/β* and *Rev-erbα/β* are regulated by CLOCK and BMAL1 via E-box, whereas their proteins RORα/β and REV-ERBα/β activate and suppress *Bmal1* by competing for binding to the RORE (retinoic-acid-related orphan receptor response element) in the *Bmal1* promoter region (Figure 3). In the third loop, D-box-containing proline and acidic amino-acid-rich basic leucine zipper (PAR bZip) genes *Dbp* (albumin D-box-binding protein), *Hlf* (hepatic leukemia factor), *Tef* (thyrotroph embryonic factor), and *E4bp4/Nfil3* (E4 promoter-binding protein 4/nuclear factor interleukin-3-regulated protein/nuclear factor, interleukin 3 regulated) are all regulated by CLOCK and BMAL1 via E-box, whereas their proteins DBP, HIF, TEF, and E4BP4/NFIL3 bind to D-box in the promoter regions of their target genes, where DBP, HIF, and TEF activate D-box-containing genes and E4BP4/NFIL3 represses them [144]. Among these three circadian-clock-controlled *cis*-elements-mediated transcriptional feedback loops, the E/E’-box-mediated loop plays the dominant role in the circadian clock [145]. However, the E/E’-box-mediated loop, combined with the RORE-mediated loop and the D-box-mediated loop, forms the necessary transcriptional repression and delays for oscillating approximately 24 h a day. In particular, E/E’-box, D-box, and RORE act in the morning, evening, and night, respectively [146,147]. In addition, the mechanistic/mammalian target of the rapamycin (mTOR) pathway, implicated in numerous neurological disorders, has been shown to contribute to circadian regulation [148,149] by activating circadian clock genes through the phosphorylation of the translation factor S6K1 [148,150,151]. Specifically, S6K1 phosphorylates GSK3β, which, in turn, phosphorylates CLOCK, BMAL1, and REV-ERB [152].

## 5. The Roles of Circadian Clock Genes in Epilepsies

Two mechanisms have been proposed to account for the effects of the circadian clock on seizures [4]: one is that canonical clock genes such as *BMAL1* and *CLOCK* contribute directly to epilepsies, and the other is that the circadian clock acts through certain signaling pathways to exert its effect on epilepsy. Loss of the circadian PAR bZip transcription factors DBP, HIF, and TEF resulted in lethal spontaneous epileptic seizures in mice [144]. TEF was shown to regulate the expression of pyridoxal kinase involved in converting B6 vitamers into pyridoxal phosphate (PLP) [144], and downregulation of PLP is associated with the susceptibility of seizures [153]. The CLOCK protein was revealed to be significantly downregulated in the neurons of human focal epilepsy patients, and the seizure threshold was reduced in excitatory pyramidal neuron-specific *Clock*^−/−^ knockout mice. Similarly, downregulation of BMAL1 was found in hippocampal sclerosis (HS) patients with HS International League Against Epilepsy (ILAE) type I and III [154], and the threshold of seizures was also reduced in *Bmal1*^−/−^ knockout mice [140]. REV-ERBα was shown to be upregulated in the brain tissues of epileptic human patients and mice, while downregulation of *Rev-erbα* in mice reduced their seizure susceptibility, and REV-ERBα activated GABA transporters *Slc6a1* (*Gat1*) and *Slc6a11* (*Gat3*) through repressing *E4bp4/Nfil3* to downregulate GABA signaling [155]. This study illuminates how circadian clock genes act through GABA signaling to exert their role in epilepsy. Together, these genetic studies have provided strong support for the functional links between the circadian clock and epilepsy.

## 6. Mutual Effects between Epilepsy and Sleep

Sleep and epilepsy are reciprocally affected. On the one hand, NREM sleep, especially NREM stage 1 (N1) and stage 2 (N2) sleep, facilitates epileptogenesis, while REM sleep inhibits it [156]. Specifically, REM sleep has the most suppressive effect during the EEG desynchronization period [157], whereas NREM sleep facilitates seizures due to the effect of the synchronous discharge of the thalamocortical network [158,159], as evidenced by the fact that 95% of seizures occur in NREM sleep [160]. An interesting study demonstrated that a small lesion in focal cortical dysplasia (FCD) type II patients is highly associated with sleep-related epilepsy [161]. Interictal epileptiform discharges (IEDs) have been used to evaluate seizure exploding [162]. Melatonin, a sleep-promoting hormone, appears to contribute to the nocturnal predominance of IEDs during sleep [163], and reduced melatonin levels with shifted phases were reported in epilepsy patients [126,164,165]. In adult patients, frontal lobe epilepsy is the archetypal sleep-related epilepsy that tends to occur during sleep, whereas juvenile myoclonic epilepsy often occurs in the morning [166]. Furthermore, sleep deprivation has been known to cause seizures in generalized epilepsies and juvenile myoclonic epilepsy [167]. Obstructive sleep apnea (OSA) has been reported to display a high prevalence in epilepsy patients, especially in drug-resistant epilepsy [168]. Conversely, increasing sleep duration by 1.6 h can reduce seizure risk by 27% in focal drug-resistant epilepsy [169].

On the other hand, epileptic activity has been shown to affect sleep continuity, which increases waking time after sleep onset, reduces REM sleep quality, and delays the first REM sleep episode in epilepsy patients [169]. Epilepsy patients often suffer from severe sleep disturbances such as excessive daytime sleepiness, sleep fragmentation, and insomnia [170]. Nocturnal seizures may lead to severe sleep fragmentation and even NREM parasomnia [171], and diurnal seizures also result in the alteration of the sleep architecture [172]. The epileptic activity also alters the sleep oscillations, likely through desynchronizing hippocampal IED and remote cortical spindles [173]. The sleep architecture of JME patients is severely altered with prolonged REM onset latency and decreased REM percentage [174]. Furthermore, most AEDs have been known to result in sleepiness, whereas some AEDs, such as levetiracetam and lamotrigine, have been shown to lead to severe insomnia [175].

Hence, sleep problems are prevalent among epilepsy patients, and reciprocal interactions between epilepsy and sleep should be underscored in epilepsy treatment. Various questionnaire-based instruments, such as the Pittsburgh Sleep Quality Index (PSQI) and the Sleep Condition Indicator, should be employed to evaluate the sleep status of patients, and possible comorbid sleep disorders also must be assessed. In particular, comorbid sleep disorders, once verified, should be treated separately. Generally, after epileptic patients are cured with drug treatment or surgeries, their sleep quality is expected to be improved with normal sleep patterns and melatonin levels [166]. Good quality of sleep helps contain seizures.

## 7. Animal Models for Epilepsies

**Figure 4:**
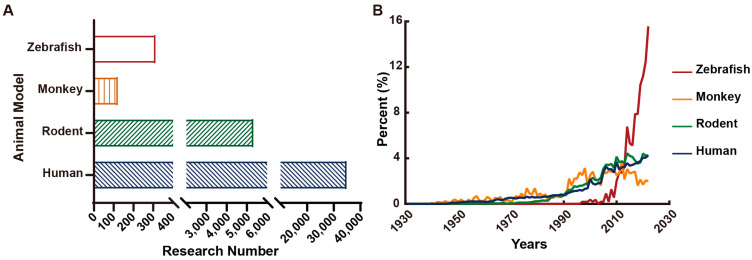
Summary of epileptic publications using different animals. (**A**) Number of epileptic publications using animal models, compiled by the Web of Science™ (https://clarivate.com/webofsciencegroup/solutions/web-of-science/, accessed on 23 February 2022) during the past 5 years. (**B**) Tendency of using different animal models for epileptic studies. Percentages of the number of published articles annually in total literature for each animal model are shown.

Animal models have played important roles in epileptic studies, especially in genetic and molecular mechanistic investigations [176]. In addition to human and some monkey epileptic studies, rodents, primarily mice and rats, are often used, and zebrafish have been increasingly used in recent years (Figure 4). In the following, we focus on pharmacological and genetic models of mice and zebrafish for epilepsies, even though electrical or acoustic stimulation of the brain has been employed to induce rodent models of seizures/epilepsies [177], which are not reviewed here.

### 7.1. Pharmacological Models

Several drugs have been widely used to evoke seizures in rodents and zebrafish (Table 3). Pentylenetetrazole (PTZ) as a GABAA receptor antagonist is the early convulsant drug [178], while (D, L)-allylglycine (AG) reduces the level of GABA biosynthesis key enzyme glutamic acid decarboxylase (GAD), leading to the depletion of GABA and the accumulation of glutamate [179]. In mice, a 300 mg/kg dose of AG is sufficient to induce 100% recurrent clonic seizures, similar to 20 mM PTZ-induced seizures. Picrotoxin, a chloride-channel blocker, can result in seizures in zebrafish with a 300 μM dose [180] and in mice with a 12 mg/kg dose [181]. Seizures evoked by picrotoxin can be suppressed by methanolic extracts of *Hyoscyamus niger* L. in mice [181]. Kainic acid (KA) causes excitotoxicity as an agonist of glutamatergic receptors and induces dosage-dependent clonus-like convulsions in zebrafish with an intraperitoneal injection at 1–8 mg/kg [182] and seizures in rats with an intracerebral injection of 0.4–2 ug [183]. Pilocarpine as a cholinergic agonist has been used to induce temporal lobe epilepsy in mice [184] but is rarely used in zebrafish [185]. Ginkgotoxin, purified from *Ginkgo biloba*, is hypothesized to inhibit GABA synthesis and can induce seizure-like behavior in zebrafish larvae [186,187]. In addition, tetanus toxin (TT) induces severe neurological disease manifested by generalized muscular convulsion in rats [188]. Adult rats displayed seizures 1–2 days after ablating interneurons by TT injection into the Cornu Ammonis 3 (CA3) hippocampal region [189]. Finally, caffeine and strychnine also induce seizures in rodents. An overdose of caffeine (400 mg/kg) as a nonselective antagonist of adenosine receptors induces epilepsy by reducing the threshold of convulsive seizures [190], and a low dose of strychnine as an antagonist of cholinergic and glycinergic receptors also induces epilepsy in mice [191]. These drugs are commonly used to establish pharmacological epilepsy models in rodents and zebrafish.

### 7.2. Genetic Models

Next-generation sequencing technology has helped uncover many new epilepsy genes [242]. Elucidation of these epilepsy genes is of great importance for the diagnosis and treatment of epilepsy. Well-established animal models for these epilepsy genes provide an efficient way to understand human pathology. Genetic models have been generated with sophisticated genetic manipulations (Table 3). Remarkably, a mutation in *SCN1A*, voltage-gated sodium channel alpha subunit 1 (VGSC), causes over 80% of Dravet syndrome cases [40,243]. Several knockout rodent models have been generated for Dravet syndrome [244,245], whereas zebrafish homozygous *scn1lab*^−/−^ mutants have also been used as an epilepsy model [246]. Except for α subunit, β subunit (*Scn1b*), type 2 (*Scn2a*), and type 8 (*Scn8a*) have also been used as genetic models for epilepsy in mice. *Scn2a* transgenic mice display spontaneous seizures [247]. In addition to epilepsy, *Scn2a* has also been implicated in other neurological disorders, including schizophrenia and autism spectrum disorder [202]. Intriguingly, one *Scn8a* mutation leading to hypoexcitation of cortical circuits results in convulsive seizure resistance, whereas the other *Scn8a* mutation leading to hyperexcitation of thalamocortical circuits causes nonconvulsive absence epilepsy [248]. The members of the KCNQ family, especially *KCNQ2* and *KCNQ3*, encode voltage-gated potassium channels (VGKC), which are associated with epilepsy, such as benign familial neonatal seizures (BFNS) [249]. In zebrafish, *kcnq2*, *kcnq3*, and *kcnq5* are expressed in early development, and inhibitors of K_v_7 channels evoke convulsive behaviors in larvae from 3 dpf (days postfertilization) to 7 dpf [236]. Homozygous *Kcnq2*^−/−^ and *Kcnq3*^−/−^ mice also exhibit severe spontaneous generalized seizures concurrent with a disturbed M-current [205]. Moreover, *Kcnj10* (potassium inwardly rectifying channel subfamily J10), expressed in glial cells, is an important causative gene for EAST (epilepsy, ataxia, sensorineural deafness, tubulopathy) syndrome [250]. In human patients, *KCNJ10* missense or nonsense mutations lead to electrolyte imbalance, seizures, and deafness [251], which are recapitulated in *Kcnj10*^−/−^ knockout mice [194]. In zebrafish, knocking down orthologs *kcnj10a* and *kcnj10b* leads to locomotor defects [235]. Furthermore, models for GABA-receptor-related genes also have been generated [252]. *Gabra1*^−/−^ knockout mice exhibit brain dysfunction, such as anxiety and seizures, and *gabra1*^−/−^ zebrafish show generalized seizures in the larval stages [233]. Zebrafish mutants for *gabrg2* [212,234] and *gabrb3* [239] have been established for epilepsy research. Other genetic models have been established with gene-editing tools such as CRISPR-Cas9 for numerous zebrafish epilepsy genes, including *arxa, eef1a2, pnpo,* and *strada*, some of which have been used for drug screens [228,233,234,239] (Table 3).

### 7.3. Circadian Rhythms of Epileptic Animal Models

These drug-induced and genetic epilepsy models are invaluable for unraveling the specific mechanisms underlying not only epileptic pathogenesis but also how the circadian clock contributes to epilepsy. Pilocarpine-induced temporal lobe epilepsy mice [184] and electrically induced post-limbic status (PLS) rats [127] were shown to display robust rhythmicity of seizures. Interestingly, transcriptome analysis of the ventral hypothalamus of pilocarpine-induced temporal lobe epilepsy mice revealed altered rhythmicity of the genes involved in oxidative phosphorylation and aerobic glycolysis [184], providing novel insights into circadian involvement in epilepsy pathogenesis. Long-term intracranial EEG monitoring with implantable devices (NeuroVista Seizure Advisory System and Summit RC + S) revealed that one human patient and seven epileptic dogs displayed robust rhythmicity of interictal epileptiform spikes (IES), and, intriguingly, thalamic deep brain stimulations (DBS) could alter IES rhythmicity in the human patient and epileptic dogs [253], indicating that the thalamocortical system is involved in regulating the circadian rhythm of epilepsy. Intriguingly, a comparison of the rhythms of chronic TLE rats evoked by chemical drugs or electrical stimuli showed that approximately 78% (7 out of 9) of these epilepsies peak in the light period when these animals sleep [254], whereas most human TLE patients peak in the afternoon, i.e., the light and wakefulness period, indicating that the robust and endogenous circadian rhythm of TLE is independent of the diurnality and nocturnality of animals. Monitoring electrical-stimulation-induced epileptic rats with hippocampal electrodes found that seizures peak at 14:05 under constant dark (DD) conditions but around 14:59 under 12–12 h light/dark (LD) conditions [255], indicating that the circadian clock likely contributes to seizures. Epileptic seizures as abnormal stimuli were shown to induce phase shifts of core body temperature (CBT) rhythms [256]. Furthermore, a comparison of the latency of evoked potentials of the dentate gyrus (DG) of electrically induced epileptic rats found that the latency was significantly reduced in the high-seizure phase (14:00 to 22:00) but not in the low-seizure phase (22:00 to 14:00) [257], indicating that limbic seizures are likely regulated by circadian excitation and inhibition of the DG in chronic epilepsy. These lines of investigation are much needed for a chronomodulated strategy-based epilepsy chronotherapy.

### 7.4. Advantages and Challenges of Epileptic Animal Models

Mice have played an important role in studies on the mechanisms of epilepsy because their anatomical structures and gene expression patterns are highly similar to those of humans [258,259]. In particular, the development of long-term EEG recordings allows for monitoring of seizures that are usually difficult to measure, including certain age-related spontaneous seizures, infrequent seizures, and nonconvulsive electrographic seizures [177].

However, several limitations of mouse epilepsy models are difficult to circumvent; for instance, some seizure models fail to recapitulate relevant human behaviors [260], and other genetic models result in fatal seizures [261,262]. Mice EEG recordings make long-term seizure monitoring possible, but the procedure also damages the brain due to the insertion of electrodes, and animals are socially isolated during seizure monitoring. Despite these challenges, mice still stand as excellent models for studying epilepsy.

The zebrafish has figured prominently as a model for epileptic studies in recent years, largely due to its large clutch size, transparent embryos, sophisticated genetic manipulation, and utility for high-throughput drug screens [263]. In 2005, Baraban et al. reported the PTZ-induced zebrafish epilepsy model and clearly showed clonus-like convulsion of larval zebrafish as a new powerful system for epileptic studies [228].

As discussed above, several pharmacological and genetic manipulations have successfully been employed to elicit robust seizure-like behavioral and neurophysiological phenotypes in both larval and adult zebrafish. High-throughput drug screens have been developed using zebrafish larvae [264], and a unique scoring system has been established to measure the seizures of adult zebrafish [265]. Light-sheet microscopy and in vivo calcium imaging with genetically encoded indicators allow for directly visualizing almost whole-brain neuronal activities and network connections during epilepsy [266], and pERK (phosphorylated extracellular signal-regulated kinase) [267,268] combined with the Map-Map method as effective biomarkers have been used to characterize neuronal circuits involved in zebrafish epilepsy models. However, the relatively primitive zebrafish behaviors have compromised their predictive power [269]. Zebrafish larvae are thought to be effective only for modeling early-onset epilepsy because of their simple movement and underdeveloped neural system [270], and the small size of zebrafish makes it challenging to use them to perform specific epilepsy interventions such as deep brain stimulation [271]. Nevertheless, as an effective and complementary model, zebrafish epilepsy studies have advanced rapidly [272].

## 8. A Chronomodulated Strategy for Epilepsy Therapy

### 8.1. Circadian Mechanisms Underlying Epileptogenesis

**Figure 5:**
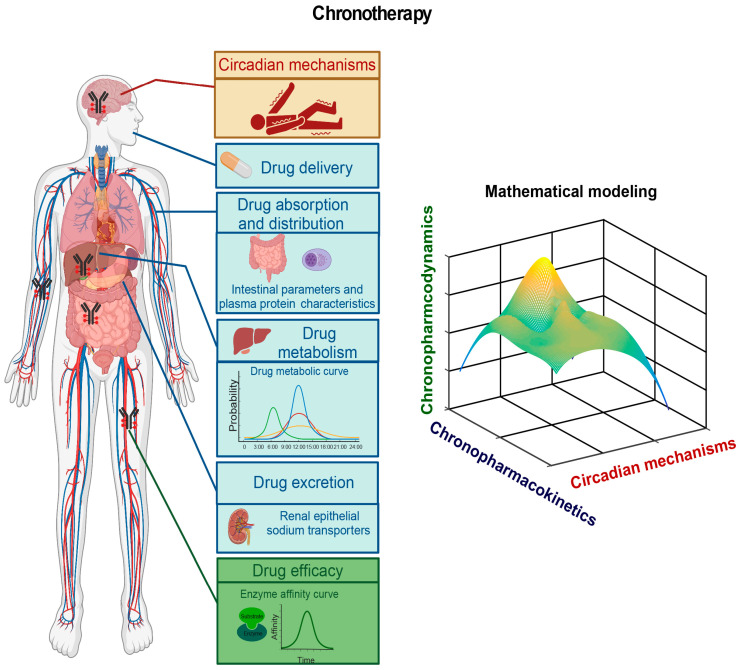
A chronomodulated strategy for epilepsy chronotherapy. Three lines of experiments are required for developing chronomodulated strategy-based epilepsy chronotherapy: (1) to investigate circadian mechanisms underlying time-of-day-specific dynamics of rhythmic epilepsies (red), (2) to investigate chronopharmacokinetics of specific AEDs (blue), and (3) to investigate chronopharmacodynamics of specific AEDs (green). The findings from these three lines of experiments should help develop chronomodulated strategy-based chronotherapy for specific rhythmic epilepsy. Mathematical/computational modeling is also needed to help select an optimal chronotherapy plan.

Although our understanding of the mechanisms underlying epilepsy remains limited, mounting evidence indicates circadian involvement in epilepsy pathogenesis, as numerous types of human epilepsies display robust daily rhythmicity [111]. Future efforts will aim to identify circadian biomarkers by elucidating molecular genetic mechanisms underlying how the circadian clock regulates the robust rhythmicity of these epilepsies. In doing so, the circadian clock system and possible circadian-clock-regulated epilepsy processes such as the hypothalamus–pituitary–adrenal (HPA) axis and the hypothalamus–pituitary–gonadal (HPG) axis should be investigated. The circadian clock regulates the HPA axis [273,274] and the HPG axis [275,276], which are known to contribute to epilepsy pathogenesis [277,278,279]. In some epilepsies, it would be worthwhile to investigate how the circadian clock acts through the HPA axis or the HPG axis to regulate epilepsy pathogenesis. Furthermore, it would be intriguing to determine whether the core circadian clock genes and circadian-clock-controlled epilepsy genes harbor mutations or whether the normal rhythmic expression patterns of these circadian clock genes and circadian-clock-controlled epilepsy genes are altered in individual patients. This line of investigation should provide insights into the circadian regulation of the dynamics of the pathogenesis of a particular epilepsy, which should provide cues for the time-of-day delivery of AEDs (Figure 5). It is important to investigate the mechanisms underlying why epilepsy displays robust rhythmicity. We have reanalyzed the circadian rhythms of epilepsy-related genes in TLE mice [184] and observed that the circadian rhythmicity of approximately 50 epileptic driver genes is altered in TLE mice, with some losing their rhythmicity, some gaining rhythmicity, and some maintaining rhythmicity [280]. For instance, *APEH* (acylaminoacyl-peptide hydrolase) was shown to be associated with valproic acid metabolism in Chinese epileptic patients [281], and its expression amplitude increased with the lengthened period in TLE mice. The specific alteration of the rhythmicity of circadian clock genes and epilepsy genes in epilepsy patients and animal models must be emphasized when developing a chronomodulated chronotherapy.

### 8.2. Pharmacokinetic and Pharmacodynamic Studies of AEDs

The circadian clock has been known to contribute to pharmacokinetics and pharmacodynamics [282]. Chronopharmacokinetics investigates how the circadian clock regulates drug absorption, distribution, metabolism, and excretion (ADME), each of which plays a critical role in regulating drug levels in the body [283,284]. In particular, peripheral molecular clocks in several vital organs, such as the intestine, liver, and drug target tissues, play a direct role in regulating blood drug levels. The absorption of oral drugs depends on the intestinal tract’s physiological parameters [285]. Considerable evidence has shown the importance of circadian clocks in intestinal physiology [286]. Recently, a study reported intestinal dysbiosis associated with a particular form of epilepsy and short-bowel syndrome in an epilepsy patient who was successfully treated with valproic acid (VPA) and levetiracetam (LEV) [287]. Further, intractable epilepsy in children is comorbid with intestinal bacterial dysbiosis [288]. In addition to the lipophilicity of drugs, the distribution of drugs is also determined by plasma protein characteristics and the transport capabilities of membrane channels [282]. Daily variations of the free fraction of valproic acid (VPA) are affected by the collective actions of albumin concentration, free fatty acid (FFA) levels, and valproate concentration [289], and both free and total plasma levels of carbamazepine (CBZ) exhibit diurnal fluctuations, which should be monitored to help adjust the dosing schedule to minimize its intermittent adverse effects [290]. Numerous transporters regulated by the circadian clock are critical for drug distribution [291,292]. Metabolism and excretion are affected by liver and kidney functions, respectively. Drug efficacy duration depends on the metabolizing speed regulated by the liver clock [293]. Both aerobic glycolysis and oxidative phosphorylation are altered in TLE mice [184], indicating that AED metabolism is also altered in epilepsy. Finally, the circadian clock also regulates the expression of renal epithelial sodium transporters, directly affecting drug excretion in mice [294,295].

Furthermore, chronopharmacodynamics focuses on how the circadian clock regulates the factors that affect drug efficacy [282]. The circadian clock acts through the genes encoding drug targets, transporters, and enzymes, as well as those involved in intracellular signaling pathways, to exert effects on drug efficacy [296]. Together, chronopharmacokinetic and chronopharmacodynamic studies of an AED should help develop a chronomodulated schedule for time-of-day-specific drug delivery to maximize its efficacy and minimize its toxicities/side effects (Figure 5).

### 8.3. Epileptic Chronotherapy

In a study with a small cohort of 17 children with nocturnal or early-morning seizures, instead of conventional administration of equal doses of AEDs in the morning and evening each day, two times the morning AED dose was delivered in the evening with the equivalent total dosage. After the 5-month differential dosing treatment, 64.7% (11/17) of patients became seizure-free, and 88.2% (15/17) experienced a ≥50% reduction in seizures [24]. AED therapy with CBZ treatment was shown to significantly reduce urinary melatonin metabolite levels of epileptic patients during 06:00–14:00 and 22:00–06:00 [126]. Further, a 5–10 mg evening melatonin delivery can effectively reduce the frequency of epileptic attacks [297]. These studies have demonstrated clear efficacies of time-of-day-specific dosing of AEDs [284,298]. Indeed, the robust rhythmicity of epilepsy/seizures allows for timing the dosing of higher levels of AEDs to be around the time when seizures peak to control seizures effectively. Chronomodulation-based chronotherapy aims at enhancing efficacy and reducing side effects through the proper timing and dosing of AEDs [298,299,300,301] (Figure 5). In addition, as numerous factors are involved, mathematical/computational modeling is also needed to help develop an optimal chronotherapy plan for a specific epilepsy (Figure 5).

## 9. Discussion

We interrogated 2 major disease gene databases, PHGKB and OMIM, as well as relevant published studies, compiled 661 epilepsy-related genes (Supplementary Table S1), and classified 192 as epilepsy causative/driver genes (Table 1). These epilepsy driver genes included those involved in GABAergic, cholinergic, glutamatergic, and dopaminergic synapses; mTOR signaling, MAPK signaling, and numerous metabolic pathways; and lysosomes, as well as those encoding various ligand-gated and voltage-gated ion channels and transcription factors (Figure 1B, Table 1), indicating the complex, polygenic, and heterogeneous nature of epilepsy. A great majority of these 192 driver genes were identified with the ever-increasing power of DNA/RNA sequencing technologies [45] but have yet to be investigated in animal models. A future endeavor will be to generate mouse and/or zebrafish models for these epilepsy driver genes, particularly employing CRISPR-Cas9-mediated base-editing tools to generate mutated animals that precisely model relevant human variants [302]. The animal models of these epilepsy driver genes will help elucidate their roles in epileptic pathogenesis and provide novel targets for AED development. In the latter case, zebrafish models should be employed to conduct large-scale high-throughput epileptic drug screens [269]. In addition, whether the remaining 469 passenger and undetermined genes contribute to epilepsy also needs to be genetically ascertained using animal models in the future. However, even though OMIM and PHGKB are widely accepted disease databases, we may have missed some epileptic genes that were not yet collected in the two disease databases. This list of 661 epilepsy-related genes will need to be revised and updated because some passenger or undetermined genes may have new experimental verification. The subjective classification may result in an inaccurate grouping. In addition, limited references likely result in data omission for the pharmacological and genetic animal models. Nevertheless, we have made efforts to avoid these situations.

Many types of human epilepsies display robust rhythmicity (Table 2) [20], which is also observed in some epileptic animal models [127,184,253]. Epileptic rhythmicity supports the notion of circadian involvement in epileptogenesis and provides a unique opportunity for developing a chronomodulated strategy-based epileptic chronotherapy. We postulate three lines of experiments for developing epileptic chronotherapy (Figure 4). First, to investigate circadian mechanisms underlying time-of-day-specific dynamics of rhythmic epilepsies, particularly to determine how the peak occurrences of epilepsies are regulated by the circadian clock genes and/or by the circadian-clock-controlled epileptic genes. The findings of this line of experiments are useful for developing new AEDs per se and for helping to determine time-of-day-specific timing for dosing AEDs. Second, to investigate the chronopharmacokinetics of specific AEDs, i.e., to determine how the circadian clock regulates the ADME of the AEDs. Third, to investigate the chronopharmacodynamics of specific AEDs, i.e., to determine how the circadian clock regulates the factors affecting the efficacy and toxicity of the AEDs. The findings from chronopharmacokinetic and chronopharmacodynamic studies, combined with the circadian investigation of rhythmic epilepsies, should help select time-of-day-specific timing for the AED delivery to maximize its efficacy but minimize its toxicity [298,299,300,301] (Figure 5).

The sleep–wake cycle is the most overt circadian rhythm [303] and also affects epilepsy [304]. The sleep status of epileptic patients cannot be ignored. Sleep disorders are commonly comorbid with epilepsy and should be separately diagnosed and treated, if verified, as a part of the epilepsy treatment. Sleep problems can wreak havoc on epilepsy treatment, but ensuring that patients have good-quality sleep helps contain epilepsy.

Even though sophisticated medical management with numerous AEDs has been well carried out in clinics, more than 30% of epilepsy patients cannot be cured. Unfortunately, they must live with unpredictable seizures, pains, and fears [305]. With approximately 1% of the population worldwide suffering from epilepsies [1], the enormous number of drug-resistant epilepsy (DRE) patients makes it necessary and urgent to develop effective therapeutics. Targeting the circadian clock and circadian-clock-regulated epileptic processes will shed light on novel aspects of epilepsy pathogenesis, provide novel targets for AED development, and promise to develop effective chronomodulated strategy-based chronotherapy for the large proportion of rhythmic epilepsy patients.

## 10. Methods

Epilepsy-related genes in PHGKB [306] and OMIM were searched for with the keyword “epilepsy and genes” and with the keyword “epilepsy” in 2022, respectively. In addition to the papers listed on PHGKB and OMIM, relevant papers in PubMed of NCBI were also searched for with the keyword “epilepsy and gene name.” If one gene had fewer than three references, all were included. Only the latest articles were included for those genes with more than three references. A total of 543 articles were cited. Those genes without a reference are still shown in Supplementary Table S1, marked with an asterisk. Two authors selected papers independently, and only English papers were selected. According to the conclusions of these relevant articles, epilepsy-related genes were classified into driver genes, passenger genes, or undetermined genes, respectively. Because this is a narrative review, no sensitivity analysis was performed. The KEGG enrichment analysis was conducted with the KOBAS database (http://kobas.cbi.pku.edu.cn/kobas3/, accessed on 8 August 2022) [307], and a Q-value less than 0.05 was selected; the top 20 ranked KEGG pathways of the 192 driver genes are shown in Supplementary Table S3. GO enrichment analysis was performed with ClueGO in Cytoscape, and those with a kappa score higher than 0.4 were selected.

## Figures and Tables

**Table 1:** KEGG groups of epilepsy driver genes.

Terms	Input Genes
Taste transduction	*CACNA1A, HCN4, GABRA5, SCN2A, GEFSP7, GABBR2, GABRA6, GABRA1, GABRA2*
GABAergic synapse	*SLC6A1, GABRA6, SLC38A3, GABRG2, GAD1, SLC12A5, ABAT, CACNA1A, GABRA5, GABBR2, GABRB3, GABRA1, GABRB2, GABRA2*
Synaptic vesicle cycle	*ATP6V0C, SLC6A1, STX1B, ATP6V1A, CACNA1A, STXBP1, CPLX1, DNM1, ATP6V0A1, SLC1A2*
Retrograde endocannabinoid signaling	*PLCB1, GABRA6, GRIA2, GABRG2, CACNA1A, GABRA5, GABRB2, GABRB3, GABRA1, GABRA2*
Glutamatergic synapse	*PLCB1, SLC38A3, GRIA2, CACNA1A, GRIK2, GRIN2A, PPP3CA, GRIN1, SLC1A2*
Cholinergic synapse	*PLCB1, CACNA1A, CHRNB2, KCNQ2, KCNQ3, CHRNA4*
Long-term potentiation	*GRIA2, PPP3CA, GRIN1, GRIN2A, PLCB1*
Dopaminergic synapse	*PLCB1, GRIA2, CACNA1A, GRIN2A, SCN1A, PPP3CA*
Thyroid hormone signaling pathway	*PDPK1, NOTCH3, MTOR, SLC2A1, PLCB1*
β-Alanine metabolism	*ABAT, GAD1, ALDH2, ALDH7A1*
Glycosylphosphatidylinositol (GPI)-anchor biosynthesis	*PIGP, PIGQ, PIGS, PIGA*
Metabolic pathways	*ST3GAL3, PIGA, ATP6V0C, ATP6V1A, ASAH1, PNPO,* *PLCB1, ATP6V0C, ACP1, ALDH2, PIGP, PIGQ, PIGS, SYNJ1, MDH2, ABAT, ALDH7A1, CAD, ALG14, GAD1, UGP2*
Amyotrophic lateral sclerosis (ALS)	*GRIA2, PPP3CA, GRIN2A, SLC1A2, GRIN1*
Nicotine addiction	*GABRA6, GRIA2, GABRG2, CHRNB2, CHRNA4, GRIN2A, CACNA1A, GABRA5, GRIN1, GABRB3, GABRA1, GABRB2, GABRA2*
Morphine addiction	*GABRA6, GABRG2, CACNA1A, GABRA5, GABBR2, GABRB3, GABRA1, GABRB2, GABRA2*
Neuroactive ligand–receptor interaction	*GABRA6, GRIA2, GABRG2, GRIK2, CHRNB2, GABRB2, GLUD1, LEPR, CHRNA4, GRIN2A, CHRNA2, GABRA5, GRIN1, GABRB3, GABRA1, GABBR2, GABRA2*
mTOR signaling pathway	*ATP6V1A, MTOR, DEPDC5, PDPK1, STRADA, NPRL3, NPRL2*
cAMP signaling pathway	*GRIA2, HCN2, HCN4, GLI3, GRIN2A, GRIN1, GABBR2*
MAPK signaling pathway	*RAPGEF2, CACNA1A, CACNA1E, NTRK2, CACNB4, MEF2C, EJM4, PPP3CA*
Lysosome	*MFSD8, AP3B2, ASAH1, ATP6V0C, SCARB2, ATP6V0A1, TPP1*
Others	*SZT2, DOCK7, HNRNPU, GEFSP4, GEFSP8, GEFSP6, SLC25A12, SLC25A22, LNPK, EJM9, EJM3, ETL6, ETL3, TRAK1, P4HTM, DALRD3, UBA5, YEATS2, TNRC6A, MARCH6, EPPS, HWE1, HWE2, SPATA5, CYFIP2, PHACTR1, CNPY3, ICK, ACTL6B, RHOBTB2, PLPBP, OXR1, EIG1, EIG2, EIG3, EIG4, EIG5, EIG7, ETL4, ETL2, FRRS1L, KCNC1, DENND5A, TRAPPC4, PACS2, DMXL2, AARS1, SEMA6B, SYN1, SIK1, EEF1A2, SCN8A, LGI1, ARHGEF9, HCN1, NHLRC1, ADAM22, ADAM10, KIF3C, SMS, PDPK1(PDK1), CDYL, KCNV2, MECP2, NIPA1, SYNGAP1, EJM1, EJM2, CYFIP1, PCDH19, TRAPPC6B, SAMD12, LGI4, TBC1D24, SETD1A, CDKL5, EFHC1, POLG, NRXN1, CNTNAP2, EPM2A, ARX, KCNA2, FOXG1, CSTB, CHRNA7, SLC9A6, KCNAB1, ZEB2, SMC1A, REST, NR2F1, MYH1, KCNT2, ARV1, CASR, GUF1, PRDM8, YWHAG, NECAP1, SLC13A5, GOSR2, LMNB2, KCNB1, CLN8, FGF12, SATB2, KCNMA1, SCN1B, KCNE1, KCNMB3, FGF12, NCDN, FBXO28, YIPF5, MED23, CELF2, KCNC2*

**Table 3:** Pharmacological and genetic epileptic models in rodents and zebrafish.

Rodents		Zebrafish	
Pharmacological Models	Genetic Models	Pharmacological Models	Genetic Models
Pentylenetetrazol (PTZ) [192], (D,L)-Allylglycine (AG) [179], Kainic acid (KA) [176,183], Picrotoxin [193], Bicuculline [193], Pilocarpine [184], Tetanus toxin [188], Caffeine [190], Strychnine [191]	*Kcnj10* [194], *Aldh7a1* [195], *Mecp2* [196], *Scn1a* [197], *Cdkl5* [198], *Syngap1* [199], *Lgi1* [200], *Ube3a* [201], *Scn2a* [202], *Scn8a* [203], *Scn1b* [204], *Kcnq2/3* [205], *Kcna1* [206], *Kcna2* [207], *Kcnmb4* [208], *Cacna1a* [209], *Gria2* [210], *Chma4* [211], *Gabrg2* [212], *Fgf13* [213], *App* [214], *Ube3a* [215], *Shank3* [216], *Cntnap2* [217], *Epm2a* [218], *Celf4* [219], *Otx1* [220], *Sv2a* [221], *Trpm2* [222], *Scamp5* [223], *Grin2a* [224], *Depdc5* [225], *Alg13* [226], *Hcn1* [227]	Pentylenetetrazol (PTZ) [228], (D,L)-Allylglycine (AG) [179], Kainic acid (KA) [182], Picrotoxin [180], Pilocarpine [229,230], Ginkgotoxin [187,231]	*scn1lab* [232], *gabra1* [233], *gabrg2* [234], *kcnj10* [235], *kcnq2/3* [236], *stx1b* [237], *chd2* [238], *arxa* [239], *eef1a2* [239], *gabrb3* [239], *pnpo* [239], *strada* [239], *lgi1a* [240], *cacna1a/b* [186], *depdc5* [241]

## Supplementary Materials

The following supporting information can be downloaded at: https://www.mdpi.com/article/10.3390/ijms24044223/s1.
