# Supplementary material for: Clocking Epilepsies: A Chronomodulated Strategy-Based Therapy for Rhythmic Seizures
Source: Int J Mol Sci. 2023 Feb 20;24(4):4223. doi: 10.3390/ijms24044223 (PMC9962262; doi:10.3390/ijms24044223)
Supplement: Supplementary file 1 [file ijms-24-04223-s001.zip › Supplementary files/Supplementary Table S1.pdf]

Table S1. 661 Epilepsy-related genes.

**Driver Genes**	*SZT2*[1,2], *ST3GAL3*[3], *DOCK7*[4], *CACNA1E*[5], *CACNA1A*[6], *HNRNPU*[7], *GEFSP4*[8], *GEFSP8*[9], *GEFSP7*[10], *GEFSP6*[11], *CAD*[12], *SLC25A12*[13], *SLC25A22*[14], *SLC12A5*[15], *SLC1A2*[16], *LNPK*[17], *EJM9*[18], *EJM4*[19], *EJM3*[20], *ETL6*[21], *ETL3*[22], *TRAK1*[23], *P4HTM*[24], *DALRD3*[25], *ATP6V1A*[26], *UBA5*[27], *YEATS2*[28], *TNRC6A*[29], *RAPGEF2*[29], *MARCH6*[30], *CPLX1*[31], *EPPS*[32], *SCARB2*[33], *HWE1*[34], *HWE2*[35], *SPATA5*[36], *CYFIP2*[37], *PHACTR1*[38,39], *CNPY3*[40], *ICK*[41], *MDH2*[42], *ACTL6B*[43], *RHOBTB2*[44], *PLPBP*[45], *OXR1*[46], *EIG1*[47], *EIG2*[48], *EIG3*[49], *EIG4*[50], *EIG5*[51], *EIG7*[52], *ETL4*[53], *ETL2*[54], *FRRS1L*[55], *DNM1*[56], *KCNMA1*[57], *KCNC1*[58], *DENND5A*[59], *TRAPPC4*[60], *PACS2*[61], *DMXL2*[62], *AP3B2*[63], *AARS1*[64], *STRADA*[65], *SEMA6B*[66], *SYNJ1*[67], *SYN1*[68], *SIK1*[69], *STX1B*[70], *NPRL3*[71], *NPRL2*[71], *DEPDC5*[71], *EEF1A2*[72], *SCN8A*[73], *SCN1B* [73], *LGI1*[74], *ARHGEF9*[75], *STXBP1*[76], *GAD1*[77], *GABBR2*[78], *GABRG2*[79], *GABRB2*[80], *GABRA1*[81], *GABRB3*[81], *GABRA5*[82], *GABRA2*[82], *NTRK2*[83], *CHRNB2*[84], *HCN1*[85], *PLCB1*[86], *NHLRC1*[87], *ADAM22*[88], *ADAM10*[89], *KIF3C*[90], *SMS*[91], *PNPO*[92], *GABRA6*[93], *PDPK1(PDK1)*[94], *ACP1*[95], *GLUD1*[96], *CDYL*[97], *KCNV2*[98], *KCNE1*[99], *MECP2*[100], *NIPA1*[101], *SYNGAP1*[102], *MTOR*[103], *EJM1*[104], *EJM2*[105], *CYFIP1*[106], *KCNQ2*[100,107,108], *PCDH19*[109-112], *SLC2A1*[111,113-115], *ALDH2*[116], *TRAPPC6B*[117], *SAMD12*[118-120], *HCN2*[121-124], *LGI4*[125,126], *TBC1D24*[94,127,128], *ATP6V0C*[94], *MFSD8*[129], *ABAT*[130,131], *NOTCH3*[132], *LEPR*[133], *SETD1A*[134,135], *SCN1A*[107,113,136], *SCN2A*[100,107,137], *CHRNA4*[138,139], *CDKL5*[100,111], *EFHC1*[140,141], *CHRNA2*[142-144], *POLG*[111,145], *NRXN1*[146], *CNTNAP2*[147,148], *EPM2A*[149], *ARX*[150,151], *GRIK2*[152,153], *MEF2C*[113,154], *KCNA2*[155], *KCNQ3*[156], *ALDH7A1*[157], *GRIA2*[158], *GRIN2A*[159], *FOXG1*[100], *CSTB*[160,161], *CHRNA7*[52], *SLC9A6*[109,162], *KCNAB1*[163,164], *PIGA*[165], *ZEB2*[166], *SMC1A*[167], *REST*[168], *SLC6A1*[137], *CACNB4*[169], *NR2F1*[90,170,171], *TPP1*[170], *GLI3*[172], *KCNMB3*[173], *MYH1*[174], *ALG14*[175], *KCNT2*[176], *ARV1*[177], *UGP2*[178], *CASR*[179], *GUF1*[180], *PRDM8*[181], *YWHAG*[182], *ASAH1*[183], *NECAP1*[184], *PIGQ*[185], *SLC13A5*[186], *GOSR2*[187], *LMNB2*[188], *KCNB1*[189], *PIGP*[190], *CLN8*[191], *FGF12*[192], *PPP3CA*[193], *SATB2*[194], *NCDN*[195], *FBXO28*[196], *SLC38A3*[197], *YIPF5*[198], *MED23*[199], *GRIN1*[200], *CELF2*[201], *KCNC2*[202], *HCN4*[203], *PIGS*[204], *ATP6V0A1*[205], *KCNA1*[206], *ADGRV1*[207]
**Passenger Genes**	*KDM5C[208],* *SNIP1*[209], *MDH1*[210], *STARD7*[211], *PRICKLE1*[212], *ENFL2*[213], *GNAO1*[214], *SLC7A6OS*[215], *IER3IP1*[216], *NARS1*[217], *CERS1*[218], *GRIN2D*[219], *FGF13*[220], *ALG13*[221,222], *NBEA*[223], *KCTD7*[224], *CACNG3*[225], *PAX6*[226], *CLCN6*[174], *NRXN3*[227], *RTN4R*[228,229], *DSG2*[230], *KCNH2*[231], *SYT1*[232], *SV2A*[233], *CLCN4*[234], *RBFOX1*[235], *SLC35A2*[236], *CD40LG*[237], *GABRE*[238], *TSC2*[239], *TSC1*[234], *SPG11*[240], *ZFYVE26(SPG15)*[241], *ATP1A3*[242],

	ATP6[243], *SLC1A1*[244], *GABRR2*[245], *GABRA4*[245], *SEPSECS*[246], *OPRM1*[247], *ALDH5A1*[248], *IL4*[249], *CAMSAP2(CAMSAP1L1)*[250], *TRPM6*[251], TRPM2[252], *GLO1*[253], *APOE*[254], *KRAS*[255], *CLOCK*[256], *IL6*[257], *BIN1*[258], *SEC24B*[259], *TPH2*[260], *SOD1*[261,262], *ABL2*[263], *NRG1*[264], *KCNJ6*[265], *BRAF*[266], *PEX10*[267], *SUCLG1*[268], *CHRFAM7A*[269], *PRRT2*[270], *SRR*[271], *INSR*[272], *GABRG3*[273], *GABBR1*[274], *SV2B*[275], *MYT1L*[227], *NF1*[276], *MIR146A*[277], *ARHGAP11B*[278], *GSTT1*[279], *BRD2*[280], *HP*[95], *VAMP2*[232,281,282], *IDH2*[283], *DRD2*[284,285], *PCDH7*[286,287], *CHRNA3*[288], *PARK2*[289,290], *CHD2*[111,234], *ABCC1*[291], *TAP1*[292], *IL6RA*[293], *MVP*[294,295], *IRS2*[272], *SLC6A3*[209], *DHCR7*[296], *GSR*[297], *GRM1*[298], *GC*[296], *MTHFD1*[299], *ITGB3*[300], *KCNQ1*[301-303], *PARS2*[304,305], *CUX2*[306], *IL1B*[307], *BDNF*[308-311], *KCNJ10*[312,313], *IDH1*[283,314], *TNF*[315], *VDR*[296], *CACNA1G*[316,317], *SLC6A11*[318], *STX1A*[282], *ANKK1*[319], *MAOA*[320], *IL1RN*[321], *KCNJ3*[265,322], *ACHE*[323], *MYH6*[230], *IQSEC2*[324,325], *VRK2*[286,326], *ZDHHC9*[327], *SCN2B*[328], *KCNT1*[329], *NIPA2*[330], *CD40*[237], *GAB2*[263,331], *TNFSF10*[332], *GPR98*[333], *CASP8*[334], *CUL4B*[325], *AP1S2*[325], *RORA*[335], *RELN*[336], *SCN4B*[337], *SCN5A*[303,338], *SCN7A*[339], *UPF3B*[325], *MBOAT7*[340], *PPDPF*[341], *UBE2A*[325], *C3*[342], *TRIM3*[343], *CHRM3*[326,344], *CHRNA5*[345], *CLCN1*[346], *FLNA*[347], *AKT1*[348], *GABRA3*[349], *EMP1*[350], *DRD4*[285], *GLUL*[351,352], *FAS*[353], *HTR2A*[354,355], *NFE2L2*[356], *AQP4*[357,358], *INHA*[359], *CDK19*[360], *CPA6*[361], *GRIN2B*[347], *PIGB*[362], *WWOX*[363], *ITPA*[364], *AGER*[365], *GOT2*[366], *SPTAN1*[367,368], *PRL*[369], *NLRP3*[370], *HIF1A*[371], *INS*[372], *RASAL2*[289], *BAI3*[289], *TNR*[289], *MACROD2*[289], *NEDD9*[289], *SOX8*[289], *HIP1*[289], *ADORA2A*[373], *ACAP3*[374], *H19*[375], *TGFBR1*[376], *NLRP1*[377], *NSF*[378]
**Undetermined Genes**	*NNMT[379],* *CNTN2*[380], *ECA1*[381], *ECT*[382], *ARSD**, *PAFAH1B1*[383], *NOS2**, *IDS*[384], *CYP24A1*[296], *CPS1*[385], *ABCC2*[386], *ITIH4*[387], *ATXN3**, *FTSJ1*[388], *SMG6**, *AP3M2*[389], *TBX21*[390], *DLG3*[391], *ME2*[392], *ATRX*[325], *ABCB1*[393], *COMT*[309], *CYP2D6*[394], *ABHD12*[395], *HNF4A*[396], *PDYN*[397], *FMR1*[398], *VIPR2*[399], *CCL2*[400], *PHOX2B*[401], *GAD2**, *GABARAP*[402], *CLCN3*[403], *ELP4*[404], *MEP1A*[140], *RHAG*[140], *SOD2*[405], *POLR3G*[333], *ABCC5*[406], *MTR*[299], *AKT3*[407], *MMP8*[408], *PIK3CA*[409], *ELOVL4*[409], *NDUFB3**, *KIAA1267**, *CLU*[410], *GJA8**, *LDB3*[230], *ATXN1**, *ATXN10**, *ATXN7**, *POR*[411], *TPH1**, *TOMM40*[412], *KHDRBS3**, *RAF1*[263], *PRKAA1*[413], *P2RX7*[413], *SNAP25*[232], *SYT11**, *LARGE**, *GSTM1*[414], *GSTM3**, *CFHR4*[415], *DSC2*[416], *KCNMB4*[417], *SUCLA2*[418], *SETDB1*[419], *SETDB2*[419], *SETBP1*[388], *IL10*[257], *TLR4*[420], *CYP2E1*[421], *CYP2A6*[422], *CYP2C9*[422], *CYP3A4*[423], *CYP2C8*[423], *CYP2B6*[394], *CYP2C19*[394], *CYP1A1*[394], *CYP4F3**, *PPIG**, *WARS**, *NR1I2*[424], *AGTR1**, *ANK2*[425], *FABP2*[426], *TNFRSF21*[140], *EGFR*[427], *CDKN2A*[263], *HMGA2*[428], *CACNA1C*[429], *CACNA1D*[430], *GSTA4*[431], *PTPRD*[431], *AFF2*[384], *SYN2*[432], *IL6R*[433], *ADCY9**, *SHANK2*[434],

	ADORA1*, CTLA4*, CDX2[435], PRKCB*, UGT1A6[436], FOXI1[437], UGT2B7[438], PTEN[439], PRNP[440,441], SLC22A1[442], LYSMD3[333], IDSP1[384], MTHFR[443], SLC17A8*, OR3A1*, F2[444], GSTA5*, C16ORF72[399], PGP[445], MED12[325], CALHM1[446,447], PDCD1*, HLA-DQA1[448], HLA-DRB1[448], HLA-DQB1[448], HLA-B[449], HLA-A[449], HLA-C[449], DYNC1H1[388], COX2*, CYTB[450], ND5[451], MIR1468*, MIR146[277], MIR196A2[452], MIR149[452], MIR499[452], TRNK*, TRNL1*, ICCA(PRRT2)[453], KIAA0415*, FAM82B*, OR5H8P*, ACCN2*, MELAS[454], LHON[454], TUBGCP5[455], MBD5[456], MICA[457], BAG6[457], ATXN2[458], SNORD94*, IGHE*, OR2A2*, DHFR[459], UGT1A[460], UGT1A4[461], UGT1A9[462], UGT1A3[463], UGT1A8[464], SHANK3[465], GOLGA8H*, OR2Q1P*, SLC6A4 [466,467], SCN9A[468,469], GRM4[470-472], CBS[473], TERT*, CYP1B1[394], SV2C*, HTR1D*, OPHN1[325,391], NOTCH4[474], ABCB6[475], APEH [476], RNF13[477], GPX1[478,479], PER3[480], SLC26A4[437], ATP1A2[481], MTRR[473,482], EPHX1[457,483,484], ABCG2[406,485], CYP3A5[486,487], SCN3A[328,488,489], IL1A*, CLCN2[490,491], UGT1A1[442], CYP1A2[492], HTR1A[493], KCNJ11[494], HLA-DQB[448,495], HTR1B*, DBH[496,497], GABRD[490,498], SLCO2B1*, ABCC4[499], RALBP1[500], TGFB1[501,502], SLC16A1[503,504], PPARG[505], RORB[335], PTGER3[293], SCN3B[506], CACNA1I[507], CAT[478], SLC16A7*, CD36[508], SLC25A27[140], KEAP1[356], SLC7A3[509], KAT2B*, SH3BGRL2*, EFHC2[510,511], PER2[480], ALLC*, PTGS2*, BAD[512], RPLP2*, SPG21[241], PMM2[513], NDE1[514], PRKCD[515], NADSYN1[296], ABCB4[516], SLC1A6[517], CCL12[518], TOR3A*, SHMT1[482], SLC22A4[519], SLC2A3[520], SP1[521], FA2H[241], USF1[522], TIMP4[523], TCN2*, TRPC4[524], CETN3[333], MBD6[419], CLCN7*, CYP2R1[296], CLCN5[525], COL1A1, GLYCTK[521], UNC5D[227], MBLAC2[333], CYP27A1[296], CYP27B1[296], AKR1B1*, DDHD2[526], F5*, HEPACAM[527], FDPS[528], FGFR1[263], AGTR1A*, DCX[529], NAGS[530], GSS*, GSTA3[531], PDE7B[532], SRPX2[382], GBA[533], GNL3[387], APP[290], CDKL4*, HTR2C[534], GSTM4*, CFH[415], DDR2[428], NMBR[532], MYH11[514], MMP9[535], KCNJ9[357,481], IDO1*, IRS1[272], ABCC6[514], GSTK1*, RHOA[229], UGDH[536], KCNK4[537,538], GAL[539], ATN1[540], MTHFS[541], NACC1[542], ATP7B*, MBLAC2[333], IFNG*, ESR1[543], FAM19A2*, RG9MTD2*, AHR*, MIRLET7B*

*: No reference.

References

1. Peng, M.; Yin, N.; Li, M.O. SZT2 dictates GATOR control of mTORC1 signalling. *Nature* **2017**, *543*, 433-437, doi:10.1038/nature21378.
2. Naseer, M.I.; Alwasiyah, M.K.; Abdulkareem, A.A.; Bajammal, R.A.; Trujillo, C.; Abu-Elmagd, M.; Jafri, M.A.; Chaudhary, A.G.; Al-Qahtani, M.H. A novel homozygous mutation in SZT2 gene in Saudi family with developmental delay, macrocephaly and epilepsy. *Genes & genomics* **2018**, *40*, 1149-1155, doi:10.1007/s13258-018-0673-5.

3. van Diepen, L.; Buettner, F.F.R.; Hoffmann, D.; Thiesler, C.T.; von Bohlen Und Halbach, O.; von Bohlen Und Halbach, V.; Jensen, L.R.; Steinemann, D.; Edvardson, S.; Elpeleg, O.; et al. A patient-specific induced pluripotent stem cell model for West syndrome caused by ST3GAL3 deficiency. *European journal of human genetics : EJHG* **2018**, *26*, 1773-1783, doi:10.1038/s41431-018-0220-5.

4. Perrault, I.; Hamdan, F.F.; Rio, M.; Capo-Chichi, J.M.; Boddaert, N.; Décarie, J.C.; Maranda, B.; Nabbout, R.; Sylvain, M.; Lortie, A.; et al. Mutations in DOCK7 in individuals with epileptic encephalopathy and cortical blindness. *American journal of human genetics* **2014**, *94*, 891-897, doi:10.1016/j.ajhg.2014.04.012.

5. Helbig, K.L.; Lauerer, R.J.; Bahr, J.C.; Souza, I.A.; Myers, C.T.; Uysal, B.; Schwarz, N.; Gandini, M.A.; Huang, S.; Keren, B.; et al. De Novo Pathogenic Variants in CACNA1E Cause Developmental and Epileptic Encephalopathy with Contractures, Macrocephaly, and Dyskinesias. *American journal of human genetics* **2018**, *103*, 666-678, doi:10.1016/j.ajhg.2018.09.006.

6. Damaj, L.; Lupien-Meilleur, A.; Lortie, A.; Riou, É.; Ospina, L.H.; Gagnon, L.; Vanasse, C.; Rossignol, E. CACNA1A haploinsufficiency causes cognitive impairment, autism and epileptic encephalopathy with mild cerebellar symptoms. *European journal of human genetics : EJHG* **2015**, *23*, 1505-1512, doi:10.1038/ejhg.2015.21.

7. Bramswig, N.C.; Lüdecke, H.J.; Hamdan, F.F.; Altmüller, J.; Beleggia, F.; Elcioglu, N.H.; Freyer, C.; Gerkes, E.H.; Demirkol, Y.K.; Knupp, K.G.; et al. Heterozygous HNRNPU variants cause early onset epilepsy and severe intellectual disability. *Human genetics* **2017**, *136*, 821-834, doi:10.1007/s00439-017-1795-6.

8. Audenaert, D.; Claes, L.; Claeys, K.G.; Deprez, L.; Van Dyck, T.; Goossens, D.; Del-Favero, J.; Van Paesschen, W.; Van Broeckhoven, C.; De Jonghe, P. A novel susceptibility locus at 2p24 for generalised epilepsy with febrile seizures plus. *Journal of medical genetics* **2005**, *42*, 947-952, doi:10.1136/jmg.2005.031393.

9. Poduri, A.; Wang, Y.; Gordon, D.; Barral-Rodriguez, S.; Barker-Cummings, C.; Ulgen, A.; Chitsazzadeh, V.; Hill, R.S.; Risch, N.; Hauser, W.A.; et al. Novel susceptibility locus at chromosome 6q16.3-22.31 in a family with GEFS+. *Neurology* **2009**, *73*, 1264-1272, doi:10.1212/WNL.0b013e3181bd10d3.

10. Singh, R.; Scheffer, I.E.; Crossland, K.; Berkovic, S.F. Generalized epilepsy with febrile seizures plus: a common childhood-onset genetic epilepsy syndrome. *Annals of neurology* **1999**, *45*, 75-81, doi:10.1002/1531-8249(199901)45:1<75::aid-art13>3.0.co;2-w.

11. Baulac, S.; Gourfinkel-An, I.; Couarch, P.; Depienne, C.; Kaminska, A.; Dulac, O.; Baulac, M.; LeGuern, E.; Nabbout, R. A novel locus for generalized epilepsy with febrile seizures plus in French families. *Archives of neurology* **2008**, *65*, 943-951, doi:10.1001/archneur.65.7.943.

12. Zhou, L.; Xu, H.; Wang, T.; Wu, Y. A Patient With CAD Deficiency Responsive to Uridine and Literature Review. *Frontiers in neurology* **2020**, *11*, 64.

13. Falk, M.J.; Li, D.; Gai, X.; McCormick, E.; Place, E.; Lasorsa, F.M.; Otieno, F.G.; Hou, C.; Kim, C.E.; Abdel-Magid, N.; et al. AGC1 Deficiency Causes Infantile Epilepsy, Abnormal Myelination, and Reduced N-Acetylaspartate. *JIMD reports* **2014**, *14*, 77-85, doi:10.1007/8904_2013_287.

14. Poduri, A.; Heinzen, E.L.; Chitsazzadeh, V.; Lasorsa, F.M.; Elhosary, P.C.; LaCoursiere, C.M.; Martin, E.; Yuskaitis, C.J.; Hill, R.S.; Atabay, K.D.; et al. SLC25A22 is a novel gene for migrating partial seizures in infancy. *Annals of neurology* **2013**, *74*, 873-882, doi:10.1002/ana.23998.

15. Stödberg, T.; McTague, A.; Ruiz, A.J.; Hirata, H.; Zhen, J.; Long, P.; Farabella, I.; Meyer, E.; Kawahara, A.; Vassallo, G.; et al. Mutations in SLC12A5 in epilepsy of infancy with migrating focal seizures. *Nature communications* **2015**, *6*, 8038, doi:10.1038/ncomms9038.
16. Stergachis, A.B.; Pujol-Giménez, J.; Gyimesi, G.; Fuster, D.; Albano, G.; Troxler, M.; Picker, J.; Rosenberg, P.A.; Bergin, A.; Peters, J.; et al. Recurrent SLC1A2 variants cause epilepsy via a dominant negative mechanism. *Annals of neurology* **2019**, *85*, 921-926, doi:10.1002/ana.25477.
17. Breuss, M.W.; Nguyen, A.; Song, Q.; Nguyen, T.; Stanley, V.; James, K.N.; Musaev, D.; Chai, G.; Wirth, S.A.; Anzenberg, P.; et al. Mutations in LNPK, Encoding the Endoplasmic Reticulum Junction Stabilizer Lunapark, Cause a Recessive Neurodevelopmental Syndrome. *American journal of human genetics* **2018**, *103*, 296-304, doi:10.1016/j.ajhg.2018.06.011.
18. Ratnapriya, R.; Vijai, J.; Kadandale, J.S.; Iyer, R.S.; Radhakrishnan, K.; Anand, A. A locus for juvenile myoclonic epilepsy maps to 2q33-q36. *Human genetics* **2010**, *128*, 123-130, doi:10.1007/s00439-010-0831-6.
19. Kapoor, A.; Ratnapriya, R.; Kuruttukulam, G.; Anand, A. A novel genetic locus for juvenile myoclonic epilepsy at chromosome 5q12-q14. *Human genetics* **2007**, *121*, 655-662, doi:10.1007/s00439-007-0360-0.
20. Greenberg, D.A.; Durner, M.; Keddache, M.; Shinnar, S.; Resor, S.R.; Moshe, S.L.; Rosenbaum, D.; Cohen, J.; Harden, C.; Kang, H.; et al. Reproducibility and complications in gene searches: linkage on chromosome 6, heterogeneity, association, and maternal inheritance in juvenile myoclonic epilepsy. *American journal of human genetics* **2000**, *66*, 508-516, doi:10.1086/302763.
21. Chahine, L.; Abou-Khalil, B.; Siren, A.; Andermann, F.; Hedera, P.; Ge, Q.; Andermann, E.; Pandolfo, M. A new locus for familial temporal lobe epilepsy on chromosome 3q. *Epilepsy Res* **2013**, *106*, 338-344, doi:10.1016/j.eplepsyres.2013.07.007.
22. Hedera, P.; Blair, M.A.; Andermann, E.; Andermann, F.; D'Agostino, D.; Taylor, K.A.; Chahine, L.; Pandolfo, M.; Bradford, Y.; Haines, J.L.; et al. Familial mesial temporal lobe epilepsy maps to chromosome 4q13.2-q21.3. *Neurology* **2007**, *68*, 2107-2112, doi:10.1212/01.wnl.0000261246.75977.89.
23. Barel, O.; Malicdan, M.C.V.; Ben-Zeev, B.; Kandel, J.; Pri-Chen, H.; Stephen, J.; Castro, I.G.; Metz, J.; Atawa, O.; Moshkovitz, S.; et al. Deleterious variants in TRAK1 disrupt mitochondrial movement and cause fatal encephalopathy. *Brain : a journal of neurology* **2017**, *140*, 568-581, doi:10.1093/brain/awx002.
24. Rahikkala, E.; Myllykoski, M.; Hinttala, R.; Vieira, P.; Nayebzadeh, N.; Weiss, S.; Plomp, A.S.; Bittner, R.E.; Kurki, M.I.; Kuismin, O.; et al. Biallelic loss-of-function P4HTM gene variants cause hypotonia, hypoventilation, intellectual disability, dysautonomia, epilepsy, and eye abnormalities (HIDEA syndrome). *Genetics in medicine : official journal of the American College of Medical Genetics* **2019**, *21*, 2355-2363, doi:10.1038/s41436-019-0503-4.
25. Lentini, J.M.; Alsaif, H.S.; Faqeih, E.; Alkuraya, F.S.; Fu, D. DALRD3 encodes a protein mutated in epileptic encephalopathy that targets arginine tRNAs for 3-methylcytosine modification. *Nature communications* **2020**, *11*, 2510, doi:10.1038/s41467-020-16321-6.
26. Fassio, A.; Esposito, A.; Kato, M.; Saitsu, H.; Mei, D.; Marini, C.; Conti, V.; Nakashima, M.; Okamoto, N.; Olmez Turker, A.; et al. De novo mutations of the ATP6V1A gene cause developmental encephalopathy with epilepsy. *Brain : a journal of neurology* **2018**, *141*, 1703-

1718, doi:10.1093/brain/awy092.
27. Mignon-Ravix, C.; Milh, M.; Kaiser, C.S.; Daniel, J.; Riccardi, F.; Cacciagli, P.; Nagara, M.; Busa, T.; Liebau, E.; Villard, L. Abnormal function of the UBA5 protein in a case of early developmental and epileptic encephalopathy with suppression-burst. *Human mutation* **2018**, *39*, 934-938, doi:10.1002/humu.23534.
28. Yeetong, P.; Pongpanich, M.; Srichomthong, C.; Assawapitaksakul, A.; Shotelersuk, V.; Tantirukdham, N.; Chunharas, C.; Suphapeetiporn, K.; Shotelersuk, V. TTTCA repeat insertions in an intron of YEATS2 in benign adult familial myoclonic epilepsy type 4. *Brain : a journal of neurology* **2019**, *142*, 3360-3366, doi:10.1093/brain/awz267.
29. Lei, X.X.; Liu, Q.; Lu, Q.; Huang, Y.; Zhou, X.Q.; Sun, H.Y.; Wu, L.W.; Cui, L.Y.; Zhang, X. TTTCA repeat expansion causes familial cortical myoclonic tremor with epilepsy. *European journal of neurology* **2019**, *26*, 513-518, doi:10.1111/ene.13848.
30. Florian, R.T.; Kraft, F.; Leitão, E.; Kaya, S.; Klebe, S.; Magnin, E.; van Rootselaar, A.F.; Buratti, J.; Kühnel, T.; Schröder, C.; et al. Unstable TTTTA/TTTCA expansions in MARCH6 are associated with Familial Adult Myoclonic Epilepsy type 3. *Nature communications* **2019**, *10*, 4919, doi:10.1038/s41467-019-12763-9.
31. Redler, S.; Strom, T.M.; Wieland, T.; Cremer, K.; Engels, H.; Distelmaier, F.; Schaper, J.; Küchler, A.; Lemke, J.R.; Jeschke, S.; et al. Variants in CPLX1 in two families with autosomal-recessive severe infantile myoclonic epilepsy and ID. *European journal of human genetics : EJHG* **2017**, *25*, 889-893, doi:10.1038/ejhg.2017.52.
32. Kinton, L.; Johnson, M.R.; Smith, S.J.; Farrell, F.; Stevens, J.; Rance, J.B.; Claudino, A.M.; Duncan, J.S.; Davis, M.B.; Wood, N.W.; et al. Partial epilepsy with pericentral spikes: a new familial epilepsy syndrome with evidence for linkage to chromosome 4p15. *Annals of neurology* **2002**, *51*, 740-749, doi:10.1002/ana.10221.
33. Rubboli, G.; Franceschetti, S.; Berkovic, S.F.; Canafoglia, L.; Gambardella, A.; Dibbens, L.M.; Riguzzi, P.; Campieri, C.; Magaudda, A.; Tassinari, C.A.; et al. Clinical and neurophysiologic features of progressive myoclonus epilepsy without renal failure caused by SCARB2 mutations. *Epilepsia* **2011**, *52*, 2356-2363, doi:10.1111/j.1528-1167.2011.03307.x.
34. Ratnapriya, R.; Satishchandra, P.; Kumar, S.D.; Gadre, G.; Reddy, R.; Anand, A. A locus for autosomal dominant reflex epilepsy precipitated by hot water maps at chromosome 10q21.3-q22.3. *Human genetics* **2009**, *125*, 541-549, doi:10.1007/s00439-009-0648-3.
35. Ratnapriya, R.; Satishchandra, P.; Dilip, S.; Gadre, G.; Anand, A. Familial autosomal dominant reflex epilepsy triggered by hot water maps to 4q24-q28. *Human genetics* **2009**, *126*, 677-683, doi:10.1007/s00439-009-0718-6.
36. Puusepp, S.; Kovacs-Nagy, R.; Alhaddad, B.; Braunisch, M.; Hoffmann, G.F.; Kotzaeridou, U.; Lichvarova, L.; Liiv, M.; Makowski, C.; Mandel, M.; et al. Compound heterozygous SPATA5 variants in four families and functional studies of SPATA5 deficiency. *European journal of human genetics : EJHG* **2018**, *26*, 407-419, doi:10.1038/s41431-017-0001-6.
37. Begemann, A.; Sticht, H.; Begtrup, A.; Vitobello, A.; Faivre, L.; Banka, S.; Alhaddad, B.; Asadollahi, R.; Becker, J.; Bierhals, T.; et al. New insights into the clinical and molecular spectrum of the novel CYFIP2-related neurodevelopmental disorder and impairment of the WRC-mediated actin dynamics. *Genetics in medicine : official journal of the American College of Medical Genetics* **2021**, *23*, 543-554, doi:10.1038/s41436-020-01011-x.
38. Ali, S.R.; Malone, T.J.; Zhang, Y.; Prechova, M.; Kaczmarek, L.K. Phactr1 regulates Slack (KCNT1)

channels via protein phosphatase 1 (PP1). *FASEB journal : official publication of the Federation of American Societies for Experimental Biology* **2020**, *34*, 1591-1601, doi:10.1096/fj.201902366R.
39. Marakhonov, A.V.; Přechová, M.; Konovalov, F.A.; Filatova, A.Y.; Zamkova, M.A.; Kanivets, I.V.; Solonichenko, V.G.; Semenova, N.A.; Zinchenko, R.A.; Treisman, R.; et al. Mutation in PHACTR1 associated with multifocal epilepsy with infantile spasms and hypsarrhythmia. *Clinical genetics* **2021**, *99*, 673-683, doi:10.1111/cge.13926.
40. Mutoh, H.; Kato, M.; Akita, T.; Shibata, T.; Wakamoto, H.; Ikeda, H.; Kitaura, H.; Aoto, K.; Nakashima, M.; Wang, T.; et al. Biallelic Variants in CNPY3, Encoding an Endoplasmic Reticulum Chaperone, Cause Early-Onset Epileptic Encephalopathy. *American journal of human genetics* **2018**, *102*, 321-329, doi:10.1016/j.ajhg.2018.01.004.
41. Bailey, J.N.; de Nijs, L.; Bai, D.; Suzuki, T.; Miyamoto, H.; Tanaka, M.; Patterson, C.; Lin, Y.C.; Medina, M.T.; Alonso, M.E.; et al. Variant Intestinal-Cell Kinase in Juvenile Myoclonic Epilepsy. *The New England journal of medicine* **2018**, *378*, 1018-1028, doi:10.1056/NEJMoa1700175.
42. Ait-El-Mkadem, S.; Dayem-Quere, M.; Gusic, M.; Chaussenot, A.; Bannwarth, S.; François, B.; Genin, E.C.; Fragaki, K.; Volker-Touw, C.L.M.; Vasnier, C.; et al. Mutations in MDH2, Encoding a Krebs Cycle Enzyme, Cause Early-Onset Severe Encephalopathy. *American journal of human genetics* **2017**, *100*, 151-159, doi:10.1016/j.ajhg.2016.11.014.
43. Bell, S.; Rousseau, J.; Peng, H.; Aouabed, Z.; Priam, P.; Theroux, J.F.; Jefri, M.; Tanti, A.; Wu, H.; Kolobova, I.; et al. Mutations in ACTL6B Cause Neurodevelopmental Deficits and Epilepsy and Lead to Loss of Dendrites in Human Neurons. *American journal of human genetics* **2019**, *104*, 815-834, doi:10.1016/j.ajhg.2019.03.022.
44. Belal, H.; Nakashima, M.; Matsumoto, H.; Yokochi, K.; Taniguchi-Ikeda, M.; Aoto, K.; Amin, M.B.; Maruyama, A.; Nagase, H.; Mizuguchi, T.; et al. De novo variants in RHOBTB2, an atypical Rho GTPase gene, cause epileptic encephalopathy. *Human mutation* **2018**, *39*, 1070-1075, doi:10.1002/humu.23550.
45. Tremiño, L.; Forcada-Nadal, A.; Rubio, V. Insight into vitamin B(6) -dependent epilepsy due to PLPBP (previously PROSC) missense mutations. *Human mutation* **2018**, *39*, 1002-1013, doi:10.1002/humu.23540.
46. Wang, J.; Rousseau, J.; Kim, E.; Ehresmann, S.; Cheng, Y.-T.; Duraine, L.; Zuo, Z.; Park, Y.-J.; Li-Kroeger, D.; Bi, W.; et al. Loss of Oxidation Resistance 1, OXR1, Is Associated with an Autosomal-Recessive Neurological Disease with Cerebellar Atrophy and Lysosomal Dysfunction. *The American Journal of Human Genetics* **2019**, *105*, 1237-1253, doi:https://doi.org/10.1016/j.ajhg.2019.11.002.
47. Winawer, M.R.; Marini, C.; Grinton, B.E.; Rabinowitz, D.; Berkovic, S.F.; Scheffer, I.E.; Ottman, R. Familial clustering of seizure types within the idiopathic generalized epilepsies. *Neurology* **2005**, *65*, 523-528, doi:10.1212/01.wnl.0000172920.34994.63.
48. Sander, T.; Schulz, H.; Saar, K.; Gennaro, E.; Riggio, M.C.; Bianchi, A.; Zara, F.; Luna, D.; Bulteau, C.; Kaminska, A.; et al. Genome search for susceptibility loci of common idiopathic generalised epilepsies. *Human molecular genetics* **2000**, *9*, 1465-1472, doi:10.1093/hmg/9.10.1465.
49. Baykan, B.; Madia, F.; Bebek, N.; Gianotti, S.; Güney, A.I.; Cine, N.; Bianchi, A.; Gökyiğit, A.; Zara, F. Autosomal recessive idiopathic epilepsy in an inbred family from Turkey: identification of a putative locus on chromosome 9q32-33. *Epilepsia* **2004**, *45*, 479-487, doi:10.1111/j.0013-9580.2004.30903.x.

50. Puranam, R.S.; Jain, S.; Kleindienst, A.M.; Saxena, S.; Kim, M.K.; Kelly Changizi, B.; Padma, M.V.; Andrews, I.; Elston, R.C.; Tiwari, H.K.; et al. A locus for generalized tonic-clonic seizure susceptibility maps to chromosome 10q25-q26. *Annals of neurology* **2005**, *58*, 449-458, doi:10.1002/ana.20598.
51. Kinirons, P.; Verlaan, D.J.; Dubé, M.P.; Poirier, J.; Deacon, C.; Lortie, A.; Clément, J.F.; Desbiens, R.; Carmant, L.; Cieuta-Walti, C.; et al. A novel locus for idiopathic generalized epilepsy in French-Canadian families maps to 10p11. *American journal of medical genetics. Part A* **2008**, *146a*, 578-584, doi:10.1002/ajmg.a.32139.
52. Helbig, I.; Mefford, H.C.; Sharp, A.J.; Guipponi, M.; Fichera, M.; Franke, A.; Muhle, H.; de Kovel, C.; Baker, C.; von Spiczak, S.; et al. 15q13.3 microdeletions increase risk of idiopathic generalized epilepsy. *Nature genetics* **2009**, *41*, 160-162, doi:10.1038/ng.292.
53. Teive, H.A.; Piovesan, E.J.; Kowacs, P.A.; Werneck, L.C. Familial occipitotemporal lobe epilepsy and migraine with visual aura: linkage to chromosome 9q new evidence for a genetic link between epilepsy and migraine. *Neurology* **2008**, *70*, 896; author reply 896-897, doi:10.1212/01.wnl.0000307659.43996.ca.
54. Gurnett, C.A.; Dobbs, M.B.; Keppel, C.R.; Pincus, E.R.; Jansen, L.A.; Bowcock, A.M. Additional evidence of a locus for complex febrile and afebrile seizures on chromosome 12q22-23.3. *Neurogenetics* **2007**, *8*, 61-63, doi:10.1007/s10048-006-0063-z.
55. Madeo, M.; Stewart, M.; Sun, Y.; Sahir, N.; Wiethoff, S.; Chandrasekar, I.; Yarrow, A.; Rosenfeld, J.A.; Yang, Y.; Cordeiro, D.; et al. Loss-of-Function Mutations in FRRS1L Lead to an Epileptic-Dyskinetic Encephalopathy. *American journal of human genetics* **2016**, *98*, 1249-1255, doi:10.1016/j.ajhg.2016.04.008.
56. De novo mutations in synaptic transmission genes including DNM1 cause epileptic encephalopathies. *American journal of human genetics* **2014**, *95*, 360-370, doi:10.1016/j.ajhg.2014.08.013.
57. Tabarki, B.; AlMajhad, N.; AlHashem, A.; Shaheen, R.; Alkuraya, F.S. Homozygous KCNMA1 mutation as a cause of cerebellar atrophy, developmental delay and seizures. *Human genetics* **2016**, *135*, 1295-1298, doi:10.1007/s00439-016-1726-y.
58. Muona, M.; Berkovic, S.F.; Dibbens, L.M.; Oliver, K.L.; Maljevic, S.; Bayly, M.A.; Joensuu, T.; Canafoglia, L.; Franceschetti, S.; Michelucci, R.; et al. A recurrent de novo mutation in KCNC1 causes progressive myoclonus epilepsy. *Nature genetics* **2015**, *47*, 39-46, doi:10.1038/ng.3144.
59. Han, C.; Alkhater, R.; Froukh, T.; Minassian, A.G.; Galati, M.; Liu, R.H.; Fotouhi, M.; Sommerfeld, J.; Alfrook, A.J.; Marshall, C.; et al. Epileptic Encephalopathy Caused by Mutations in the Guanine Nucleotide Exchange Factor DENND5A. *American journal of human genetics* **2016**, *99*, 1359-1367, doi:10.1016/j.ajhg.2016.10.006.
60. Van Bergen, N.J.; Guo, Y.; Al-Deri, N.; Lipatova, Z.; Stanga, D.; Zhao, S.; Murtazina, R.; Gyurkovska, V.; Pehlivan, D.; Mitani, T.; et al. Deficiencies in vesicular transport mediated by TRAPPC4 are associated with severe syndromic intellectual disability. *Brain : a journal of neurology* **2020**, *143*, 112-130, doi:10.1093/brain/awz374.
61. Olson, H.E.; Jean-Marçais, N.; Yang, E.; Heron, D.; Tatton-Brown, K.; van der Zwaag, P.A.; Bijlsma, E.K.; Krock, B.L.; Backer, E.; Kamsteeg, E.J.; et al. A Recurrent De Novo PACS2 Heterozygous Missense Variant Causes Neonatal-Onset Developmental Epileptic Encephalopathy, Facial Dysmorphism, and Cerebellar Dysgenesis. *American journal of human genetics* **2018**, *102*, 995-1007, doi:10.1016/j.ajhg.2018.03.005.

62. Esposito, A.; Falace, A.; Wagner, M.; Gal, M.; Mei, D.; Conti, V.; Pisano, T.; Aprile, D.; Cerullo, M.S.; De Fusco, A.; et al. Biallelic DMXL2 mutations impair autophagy and cause Ohtahara syndrome with progressive course. *Brain : a journal of neurology* **2019**, *142*, 3876-3891, doi:10.1093/brain/awz326.
63. Assoum, M.; Philippe, C.; Isidor, B.; Perrin, L.; Makrythanasis, P.; Sondheimer, N.; Paris, C.; Douglas, J.; Lesca, G.; Antonarakis, S.; et al. Autosomal-Recessive Mutations in AP3B2, Adaptor-Related Protein Complex 3 Beta 2 Subunit, Cause an Early-Onset Epileptic Encephalopathy with Optic Atrophy. *American journal of human genetics* **2016**, *99*, 1368-1376, doi:10.1016/j.ajhg.2016.10.009.
64. Simons, C.; Griffin, L.B.; Helman, G.; Golas, G.; Pizzino, A.; Bloom, M.; Murphy, J.L.; Crawford, J.; Evans, S.H.; Topper, S.; et al. Loss-of-function alanyl-tRNA synthetase mutations cause an autosomal-recessive early-onset epileptic encephalopathy with persistent myelination defect. *American journal of human genetics* **2015**, *96*, 675-681, doi:10.1016/j.ajhg.2015.02.012.
65. Nelson, K.; Jackman, C.; Bell, J.; Shih, C.S.; Payne, K.; Dlouhy, S.; Walsh, L. Novel Homozygous Deletion in STRADA Gene Associated With Polyhydramnios, Megalencephaly, and Epilepsy in 2 Siblings: Implications for Diagnosis and Treatment. *Journal of child neurology* **2018**, *33*, 925-929, doi:10.1177/0883073818802724.
66. Hamanaka, K.; Imagawa, E.; Koshimizu, E.; Miyatake, S.; Tohyama, J.; Yamagata, T.; Miyauchi, A.; Ekhilevitch, N.; Nakamura, F.; Kawashima, T.; et al. De Novo Truncating Variants in the Last Exon of SEMA6B Cause Progressive Myoclonic Epilepsy. *American journal of human genetics* **2020**, *106*, 549-558, doi:10.1016/j.ajhg.2020.02.011.
67. Hardies, K.; Cai, Y.; Jardel, C.; Jansen, A.C.; Cao, M.; May, P.; Djémié, T.; Hachon Le Camus, C.; Keymolen, K.; Deconinck, T.; et al. Loss of SYNJ1 dual phosphatase activity leads to early onset refractory seizures and progressive neurological decline. *Brain : a journal of neurology* **2016**, *139*, 2420-2430, doi:10.1093/brain/aww180.
68. Fassio, A.; Patry, L.; Congia, S.; Onofri, F.; Piton, A.; Gauthier, J.; Pozzi, D.; Messa, M.; Defranchi, E.; Fadda, M.; et al. SYN1 loss-of-function mutations in autism and partial epilepsy cause impaired synaptic function. *Human molecular genetics* **2011**, *20*, 2297-2307, doi:10.1093/hmg/ddr122.
69. Pröschel, C.; Hansen, J.N.; Ali, A.; Tuttle, E.; Lacagnina, M.; Buscaglia, G.; Halterman, M.W.; Paciorkowski, A.R. Epilepsy-causing sequence variations in SIK1 disrupt synaptic activity response gene expression and affect neuronal morphology. *European journal of human genetics : EJHG* **2017**, *25*, 216-221, doi:10.1038/ejhg.2016.145.
70. Vardar, G.; Gerth, F.; Schmitt, X.J.; Rautenstrauch, P.; Trimbuch, T.; Schubert, J.; Lerche, H.; Rosenmund, C.; Freund, C. Epilepsy-causing STX1B mutations translate altered protein functions into distinct phenotypes in mouse neurons. *Brain : a journal of neurology* **2020**, *143*, 2119-2138, doi:10.1093/brain/awaa151.
71. Iffland, P.H., 2nd; Carson, V.; Bordey, A.; Crino, P.B. GATORopathies: The role of amino acid regulatory gene mutations in epilepsy and cortical malformations. *Epilepsia* **2019**, *60*, 2163-2173, doi:10.1111/epi.16370.
72. Lam, W.W.; Millichap, J.J.; Soares, D.C.; Chin, R.; McLellan, A.; FitzPatrick, D.R.; Elmslie, F.; Lees, M.M.; Schaefer, G.B.; Abbott, C.M. Novel de novo EEF1A2 missense mutations causing epilepsy and intellectual disability. *Molecular genetics & genomic medicine* **2016**, *4*, 465-474, doi:10.1002/mgg3.219.

73. Zaman, T.; Helbig, I.; Božović, I.B.; DeBrosse, S.D.; Bergqvist, A.C.; Wallis, K.; Medne, L.; Maver, A.; Peterlin, B.; Helbig, K.L.; et al. Mutations in SCN3A cause early infantile epileptic encephalopathy. *Annals of neurology* **2018**, *83*, 703-717, doi:10.1002/ana.25188.
74. van Sonderen, A.; Thijs, R.D.; Coenders, E.C.; Jiskoot, L.C.; Sanchez, E.; de Bruijn, M.A.; van Coevorden-Hameete, M.H.; Wirtz, P.W.; Schreurs, M.W.; Sillevis Smitt, P.A.; et al. Anti-LGI1 encephalitis: Clinical syndrome and long-term follow-up. *Neurology* **2016**, *87*, 1449-1456, doi:10.1212/wnl.0000000000003173.
75. Klein, K.M.; Pendziwiat, M.; Eilam, A.; Gilad, R.; Blatt, I.; Rosenow, F.; Kanaan, M.; Helbig, I.; Afawi, Z. The phenotypic spectrum of ARHGEF9 includes intellectual disability, focal epilepsy and febrile seizures. *Journal of neurology* **2017**, *264*, 1421-1425, doi:10.1007/s00415-017-8539-3.
76. Stamberger, H.; Nikanorova, M.; Willemsen, M.H.; Accorsi, P.; Angriman, M.; Baier, H.; Benkel-Herrenbrueck, I.; Benoit, V.; Budetta, M.; Caliebe, A.; et al. STXBP1 encephalopathy: A neurodevelopmental disorder including epilepsy. *Neurology* **2016**, *86*, 954-962, doi:10.1212/wnl.0000000000002457.
77. Neuray, C.; Maroofian, R.; Scala, M.; Sultan, T.; Pai, G.S.; Mojarrad, M.; Khashab, H.E.; deHoll, L.; Yue, W.; Alsaif, H.S.; et al. Early-infantile onset epilepsy and developmental delay caused by bi-allelic GAD1 variants. *Brain : a journal of neurology* **2020**, *143*, 2388-2397, doi:10.1093/brain/awaa178.
78. Yoo, Y.; Jung, J.; Lee, Y.N.; Lee, Y.; Cho, H.; Na, E.; Hong, J.; Kim, E.; Lee, J.S.; Lee, J.S.; et al. GABBR2 mutations determine phenotype in rett syndrome and epileptic encephalopathy. *Annals of neurology* **2017**, *82*, 466-478, doi:10.1002/ana.25032.
79. Pedersen, M.; Kowalczyk, M.; Omidvarnia, A.; Perucca, P.; Gooley, S.; Petrou, S.; Scheffer, I.E.; Berkovic, S.F.; Jackson, G.D. Human GABRG2 generalized epilepsy: Increased somatosensory and striatothalamic connectivity. *Neurology. Genetics* **2019**, *5*, e340, doi:10.1212/nxg.0000000000000340.
80. Kumari, R.; Lakhan, R.; Kalita, J.; Garg, R.K.; Misra, U.K.; Mittal, B. Potential role of GABAA receptor subunit; GABRA6, GABRB2 and GABRR2 gene polymorphisms in epilepsy susceptibility and pharmacotherapy in North Indian population. *Clinica chimica acta; international journal of clinical chemistry* **2011**, *412*, 1244-1248, doi:10.1016/j.cca.2011.03.018.
81. Liu, J.; Tong, L.; Song, S.; Niu, Y.; Li, J.; Wu, X.; Zhang, J.; Zai, C.C.; Luo, F.; Wu, J.; et al. Novel and de novo mutations in pediatric refractory epilepsy. *Mol Brain* **2018**, *11*, 48, doi:10.1186/s13041-018-0392-5.
82. Butler, K.M.; Moody, O.A.; Schuler, E.; Coryell, J.; Alexander, J.J.; Jenkins, A.; Escayg, A. De novo variants in GABRA2 and GABRA5 alter receptor function and contribute to early-onset epilepsy. *Brain : a journal of neurology* **2018**, *141*, 2392-2405, doi:10.1093/brain/awy171.
83. Torres, C.M.; Siebert, M.; Bock, H.; Mota, S.M.; Krammer, B.R.; Duarte, J.; Bragatti, J.A.; Castan, J.U.; de Castro, L.A.; Saraiva-Pereira, M.L.; et al. NTRK2 (TrkB gene) variants and temporal lobe epilepsy: A genetic association study. *Epilepsy Res* **2017**, *137*, 1-8, doi:10.1016/j.eplepsyres.2017.08.010.
84. Lerche, H.; Jurkat-Rott, K.; Lehmann-Horn, F. Ion channels and epilepsy. *American journal of medical genetics* **2001**, *106*, 146-159, doi:10.1002/ajmg.1582.
85. Marini, C.; Porro, A.; Rastetter, A.; Dalle, C.; Rivolta, I.; Bauer, D.; Oegema, R.; Nava, C.; Parrini,

E.; Mei, D.; et al. HCN1 mutation spectrum: from neonatal epileptic encephalopathy to benign generalized epilepsy and beyond. *Brain : a journal of neurology* **2018**, *141*, 3160-3178, doi:10.1093/brain/awy263.

86. Ngoh, A.; McTague, A.; Wentzensen, I.M.; Meyer, E.; Applegate, C.; Kossoff, E.H.; Batista, D.A.; Wang, T.; Kurian, M.A. Severe infantile epileptic encephalopathy due to mutations in PLCB1: expansion of the genotypic and phenotypic disease spectrum. *Developmental medicine and child neurology* **2014**, *56*, 1124-1128, doi:10.1111/dmcn.12450.

87. Singh, S.; Sethi, I.; Francheschetti, S.; Riggio, C.; Avanzini, G.; Yamakawa, K.; Delgado-Escueta, A.V.; Ganesh, S. Novel NHLRC1 mutations and genotype-phenotype correlations in patients with Lafora's progressive myoclonic epilepsy. *Journal of medical genetics* **2006**, *43*, e48, doi:10.1136/jmg.2005.039479.

88. Yamagata, A.; Miyazaki, Y.; Yokoi, N.; Shigematsu, H.; Sato, Y.; Goto-Ito, S.; Maeda, A.; Goto, T.; Sanbo, M.; Hirabayashi, M.; et al. Structural basis of epilepsy-related ligand-receptor complex LGI1-ADAM22. *Nature communications* **2018**, *9*, 1546, doi:10.1038/s41467-018-03947-w.

89. Prox, J.; Bernreuther, C.; Altmeppen, H.; Grendel, J.; Glatzel, M.; D'Hooge, R.; Stroobants, S.; Ahmed, T.; Balschun, D.; Willem, M.; et al. Postnatal disruption of the disintegrin/metalloproteinase ADAM10 in brain causes epileptic seizures, learning deficits, altered spine morphology, and defective synaptic functions. *The Journal of neuroscience : the official journal of the Society for Neuroscience* **2013**, *33*, 12915-12928, 12928a, doi:10.1523/jneurosci.5910-12.2013.

90. Dimassi, S.; Labalme, A.; Ville, D.; Calender, A.; Mignot, C.; Boutry-Kryza, N.; de Bellescize, J.; Rivier-Ringenbach, C.; Bourel-Ponchel, E.; Cheillan, D.; et al. Whole-exome sequencing improves the diagnosis yield in sporadic infantile spasm syndrome. *Clinical genetics* **2016**, *89*, 198-204, doi:10.1111/cge.12636.

91. Peron, A.; Spaccini, L.; Norris, J.; Bova, S.M.; Selicorni, A.; Weber, G.; Wood, T.; Schwartz, C.E.; Mastrangelo, M. Snyder-Robinson syndrome: a novel nonsense mutation in spermine synthase and expansion of the phenotype. *American journal of medical genetics. Part A* **2013**, *161a*, 2316-2320, doi:10.1002/ajmg.a.36116.

92. Mills, P.B.; Camuzeaux, S.S.; Footitt, E.J.; Mills, K.A.; Gissen, P.; Fisher, L.; Das, K.B.; Varadkar, S.M.; Zuberi, S.; McWilliam, R.; et al. Epilepsy due to PNPO mutations: genotype, environment and treatment affect presentation and outcome. *Brain : a journal of neurology* **2014**, *137*, 1350-1360, doi:10.1093/brain/awu051.

93. Hernandez, C.C.; Gurba, K.N.; Hu, N.; Macdonald, R.L. The GABRA6 mutation, R46W, associated with childhood absence epilepsy, alters 6β22 and 6β2 GABA(A) receptor channel gating and expression. *The Journal of physiology* **2011**, *589*, 5857-5878, doi:10.1113/jphysiol.2011.218883.

94. Mucha, B.E.; Banka, S.; Ajeawung, N.F.; Molidperee, S.; Chen, G.G.; Koenig, M.K.; Adejumo, R.B.; Till, M.; Harbord, M.; Perrier, R.; et al. A new microdeletion syndrome involving TBC1D24, ATP6V0C, and PDPK1 causes epilepsy, microcephaly, and developmental delay. *Genetics in medicine : official journal of the American College of Medical Genetics* **2019**, *21*, 1058-1064, doi:10.1038/s41436-018-0290-3.

95. Gloria-Bottini, F.; Lucarelli, P.; Saccucci, P.; Cozzoli, E.; Cerminara, C.; Curatolo, P.; Bottini, E. Genetic polymorphism and idiopathic generalized epilepsy. Evidence of interaction between haptoglobin and ACP1 systems. *Neuropediatrics* **2008**, *39*, 357-358, doi:10.1055/s-0029-

1202834.
96. Kapoor, R.R.; Flanagan, S.E.; Fulton, P.; Chakrapani, A.; Chadefaux, B.; Ben-Omran, T.; Banerjee, I.; Shield, J.P.; Ellard, S.; Hussain, K. Hyperinsulinism-hyperammonaemia syndrome: novel mutations in the GLUD1 gene and genotype-phenotype correlations. *European journal of endocrinology* **2009**, *161*, 731-735, doi:10.1530/eje-09-0615.
97. Qin, R.; Cao, S.; Lyu, T.; Qi, C.; Zhang, W.; Wang, Y. CDYL Deficiency Disrupts Neuronal Migration and Increases Susceptibility to Epilepsy. *Cell reports* **2017**, *18*, 380-390, doi:10.1016/j.celrep.2016.12.043.
98. Jorge, B.S.; Campbell, C.M.; Miller, A.R.; Rutter, E.D.; Gurnett, C.A.; Vanoye, C.G.; George, A.L., Jr.; Kearney, J.A. Voltage-gated potassium channel KCNV2 (Kv8.2) contributes to epilepsy susceptibility. *Proceedings of the National Academy of Sciences of the United States of America* **2011**, *108*, 5443-5448, doi:10.1073/pnas.1017539108.
99. Barro-Soria, R.; Rebolledo, S.; Liin, S.I.; Perez, M.E.; Sampson, K.J.; Kass, R.S.; Larsson, H.P. KCNE1 divides the voltage sensor movement in KCNQ1/KCNE1 channels into two steps. *Nature communications* **2014**, *5*, 3750, doi:10.1038/ncomms4750.
100. Lindy, A.S.; Stosser, M.B.; Butler, E.; Downtain-Pickersgill, C.; Shanmugham, A.; Retterer, K.; Brandt, T.; Richard, G.; McKnight, D.A. Diagnostic outcomes for genetic testing of 70 genes in 8565 patients with epilepsy and neurodevelopmental disorders. *Epilepsia* **2018**, *59*, 1062-1071, doi:10.1111/epi.14074.
101. Tanti, M.; Cairns, D.; Mirza, N.; McCann, E.; Young, C. Is NIPA1-associated hereditary spastic paraplegia always 'pure'? Further evidence of motor neurone disease and epilepsy as rare manifestations. *Neurogenetics* **2020**, *21*, 305-308, doi:10.1007/s10048-020-00619-0.
102. Vlaskamp, D.R.M.; Shaw, B.J.; Burgess, R.; Mei, D.; Montomoli, M.; Xie, H.; Myers, C.T.; Bennett, M.F.; XiangWei, W.; Williams, D.; et al. SYNGAP1 encephalopathy: A distinctive generalized developmental and epileptic encephalopathy. *Neurology* **2019**, *92*, e96-e107, doi:10.1212/wnl.0000000000006729.
103. Switon, K.; Kotulska, K.; Janusz-Kaminska, A.; Zmorzynska, J.; Jaworski, J. Molecular neurobiology of mTOR. *Neuroscience* **2017**, *341*, 112-153, doi:10.1016/j.neuroscience.2016.11.017.
104. Sander, T.; Bockenkamp, B.; Hildmann, T.; Blasczyk, R.; Kretz, R.; Wienker, T.F.; Volz, A.; Schmitz, B.; Beck-Mannagetta, G.; Riess, O.; et al. Refined mapping of the epilepsy susceptibility locus EJM1 on chromosome 6. *Neurology* **1997**, *49*, 842-847, doi:10.1212/wnl.49.3.842.
105. Taske, N.L.; Williamson, M.P.; Makoff, A.; Bate, L.; Curtis, D.; Kerr, M.; Kjeldsen, M.J.; Pang, K.A.; Sundqvist, A.; Friis, M.L.; et al. Evaluation of the positional candidate gene CHRNA7 at the juvenile myoclonic epilepsy locus (EJM2) on chromosome 15q13-14. *Epilepsy Res* **2002**, *49*, 157-172, doi:10.1016/s0920-1211(02)00027-x.
106. Nebel, R.A.; Zhao, D.; Pedrosa, E.; Kirschen, J.; Lachman, H.M.; Zheng, D.; Abrahams, B.S. Reduced CYFIP1 in Human Neural Progenitors Results in Dysregulation of Schizophrenia and Epilepsy Gene Networks. *PloS one* **2016**, *11*, e0148039, doi:10.1371/journal.pone.0148039.
107. Costain, G.; Cordeiro, D.; Matviychuk, D.; Mercimek-Andrews, S. Clinical Application of Targeted Next-Generation Sequencing Panels and Whole Exome Sequencing in Childhood Epilepsy. *Neuroscience* **2019**, *418*, 291-310, doi:10.1016/j.neuroscience.2019.08.016.
108. Symonds, J.D.; Zuberi, S.M.; Stewart, K.; McLellan, A.; O'Regan, M.; MacLeod, S.; Jollands, A.; Joss, S.; Kirkpatrick, M.; Brunklaus, A.; et al. Incidence and phenotypes of childhood-onset

genetic epilepsies: a prospective population-based national cohort. *Brain : a journal of neurology* **2019**, *142*, 2303-2318, doi:10.1093/brain/awz195.

109. Mercimek-Mahmutoglu, S.; Patel, J.; Cordeiro, D.; Hewson, S.; Callen, D.; Donner, E.J.; Hahn, C.D.; Kannu, P.; Kobayashi, J.; Minassian, B.A.; et al. Diagnostic yield of genetic testing in epileptic encephalopathy in childhood. *Epilepsia* **2015**, *56*, 707-716, doi:10.1111/epi.12954.

110. Stosser, M.B.; Lindy, A.S.; Butler, E.; Retterer, K.; Piccirillo-Stosser, C.M.; Richard, G.; McKnight, D.A. High frequency of mosaic pathogenic variants in genes causing epilepsy-related neurodevelopmental disorders. *Genetics in medicine : official journal of the American College of Medical Genetics* **2018**, *20*, 403-410, doi:10.1038/gim.2017.114.

111. Ortega-Moreno, L.; Giráldez, B.G.; Soto-Insuga, V.; Losada-Del Pozo, R.; Rodrigo-Moreno, M.; Alarcón-Morcillo, C.; Sánchez-Martín, G.; Díaz-Gómez, E.; Guerrero-López, R.; Serratosa, J.M. Molecular diagnosis of patients with epilepsy and developmental delay using a customized panel of epilepsy genes. *PloS one* **2017**, *12*, e0188978, doi:10.1371/journal.pone.0188978.

112. Tsai, M.H.; Chan, C.K.; Chang, Y.C.; Lin, C.H.; Liou, C.W.; Chang, W.N.; Ng, C.C.; Lim, K.S.; Hwang, D.Y. Molecular Genetic Characterization of Patients With Focal Epilepsy Using a Customized Targeted Resequencing Gene Panel. *Frontiers in neurology* **2018**, *9*, 515, doi:10.3389/fneur.2018.00515.

113. Johannesen, K.M.; Nikanorova, N.; Marjanovic, D.; Pavbro, A.; Larsen, L.H.G.; Rubboli, G.; Møller, R.S. Utility of genetic testing for therapeutic decision-making in adults with epilepsy. *Epilepsia* **2020**, *61*, 1234-1239, doi:10.1111/epi.16533.

114. Altıokka-Uzun, G.; Özdemir, Ö.; Uğur-İşeri, S.; Bebek, N.; Gürses, C.; Özbek, U.; Baykan, B. Investigation of SLC2A1 gene variants in genetic generalized epilepsy patients with eyelid myoclonia. *Epileptic disorders : international epilepsy journal with videotape* **2018**, *20*, 396-400, doi:10.1684/epd.2018.0998.

115. Mullen, S.A.; Berkovic, S.F. Genetic generalized epilepsies. *Epilepsia* **2018**, *59*, 1148-1153, doi:10.1111/epi.14042.

116. Yang, H.; Song, Z.; Yang, G.P.; Zhang, B.K.; Chen, M.; Wu, T.; Guo, R. The ALDH2 rs671 polymorphism affects post-stroke epilepsy susceptibility and plasma 4-HNE levels. *PloS one* **2014**, *9*, e109634, doi:10.1371/journal.pone.0109634.

117. Marin-Valencia, I.; Novarino, G.; Johansen, A.; Rosti, B.; Issa, M.Y.; Musaev, D.; Bhat, G.; Scott, E.; Silhavy, J.L.; Stanley, V.; et al. A homozygous founder mutation in TRAPPC6B associates with a neurodevelopmental disorder characterised by microcephaly, epilepsy and autistic features. *Journal of medical genetics* **2018**, *55*, 48-54, doi:10.1136/jmedgenet-2017-104627.

118. Mahadevan, R.; Bhoyar, R.C.; Viswanathan, N.; Rajagopal, R.E.; Essaki, B.; Suroliya, V.; Chelladurai, R.; Sankaralingam, S.; Shanmugam, G.; Vayanakkan, S.; et al. Genomic analysis of patients in a South Indian Community with autosomal dominant cortical tremor, myoclonus and epilepsy suggests a founder repeat expansion mutation in the SAMD12 gene. *Brain communications* **2021**, *3*, fcaa214, doi:10.1093/braincomms/fcaa214.

119. Cen, Z.; Chen, Y.; Yang, D.; Zhu, Q.; Chen, S.; Chen, X.; Wang, B.; Xie, F.; Ouyang, Z.; Jiang, Z.; et al. Intronic (TTTGA)(n) insertion in SAMD12 also causes familial cortical myoclonic tremor with epilepsy. *Movement disorders : official journal of the Movement Disorder Society* **2019**, *34*, 1571-1576, doi:10.1002/mds.27832.

120. Terasaki, A.; Nakamura, M.; Urata, Y.; Hiwatashi, H.; Yokoyama, I.; Yasuda, T.; Onuma, T.; Wada, K.; Kaneko, S.; Kan, R.; et al. DNA analysis of benign adult familial myoclonic epilepsy reveals

associations between the pathogenic TTTCA repeat insertion in SAMD12 and the nonpathogenic TTTTA repeat expansion in TNRC6A. *Journal of human genetics* **2020**, doi:10.1038/s10038-020-00855-0.
121. Li, M.; Maljevic, S.; Phillips, A.M.; Petrovski, S.; Hildebrand, M.S.; Burgess, R.; Mount, T.; Zara, F.; Striano, P.; Schubert, J.; et al. Gain-of-function HCN2 variants in genetic epilepsy. *Human mutation* **2018**, *39*, 202-209, doi:10.1002/humu.23357.
122. Wu, S.Z.; Ye, H.; Yang, X.G.; Lu, Z.L.; Qu, Q.; Qu, J. Case-control pharmacogenetic study of HCN1/HCN2 variants and genetic generalized epilepsies. *Clinical and experimental pharmacology & physiology* **2018**, *45*, 226-233, doi:10.1111/1440-1681.12877.
123. Dibbens, L.M.; Reid, C.A.; Hodgson, B.; Thomas, E.A.; Phillips, A.M.; Gazina, E.; Cromer, B.A.; Clarke, A.L.; Baram, T.Z.; Scheffer, I.E.; et al. Augmented currents of an HCN2 variant in patients with febrile seizure syndromes. *Annals of neurology* **2010**, *67*, 542-546, doi:10.1002/ana.21909.
124. Tang, B.; Sander, T.; Craven, K.B.; Hempelmann, A.; Escayg, A. Mutation analysis of the hyperpolarization-activated cyclic nucleotide-gated channels HCN1 and HCN2 in idiopathic generalized epilepsy. *Neurobiology of disease* **2008**, *29*, 59-70, doi:10.1016/j.nbd.2007.08.006.
125. Ishii, A.; Zhang, B.; Kaneko, S.; Hirose, S. Positive association between benign familial infantile convulsions and LGI4. *Brain & development* **2010**, *32*, 538-543, doi:10.1016/j.braindev.2009.09.006.
126. Gu, W.; Sander, T.; Becker, T.; Steinlein, O.K. Genotypic association of exonic LGI4 polymorphisms and childhood absence epilepsy. *Neurogenetics* **2004**, *5*, 41-44, doi:10.1007/s10048-003-0158-8.
127. Carecchio, M.; Mencacci, N.E. Emerging Monogenic Complex Hyperkinetic Disorders. *Current neurology and neuroscience reports* **2017**, *17*, 97, doi:10.1007/s11910-017-0806-2.
128. Bakhchane, A.; Charif, M.; Salime, S.; Boulouiz, R.; Nahili, H.; Roky, R.; Lenaers, G.; Barakat, A. Recessive TBC1D24 Mutations Are Frequent in Moroccan Non-Syndromic Hearing Loss Pedigrees. *PloS one* **2015**, *10*, e0138072, doi:10.1371/journal.pone.0138072.
129. Lemke, J.R.; Riesch, E.; Scheurenbrand, T.; Schubach, M.; Wilhelm, C.; Steiner, I.; Hansen, J.; Courage, C.; Gallati, S.; Bürki, S.; et al. Targeted next generation sequencing as a diagnostic tool in epileptic disorders. *Epilepsia* **2012**, *53*, 1387-1398, doi:10.1111/j.1528-1167.2012.03516.x.
130. Li, X.; Zhang, J.; Wu, X.; Yan, H.; Zhang, Y.; He, R.H.; Tang, Y.J.; He, Y.J.; Tan, D.; Mao, X.Y.; et al. Polymorphisms of ABAT, SCN2A and ALDH5A1 may affect valproic acid responses in the treatment of epilepsy in Chinese. *Pharmacogenomics* **2016**, *17*, 2007-2014, doi:10.2217/pgs-2016-0093.
131. Besse, A.; Petersen, A.K.; Hunter, J.V.; Appadurai, V.; Lalani, S.R.; Bonnen, P.E. Personalized medicine approach confirms a milder case of ABAT deficiency. *Mol Brain* **2016**, *9*, 93, doi:10.1186/s13041-016-0273-8.
132. Ragno, M.; Sanguigni, S.; Manca, A.; Pianese, L.; Paci, C.; Berbellini, A.; Cozzolino, V.; Gobbato, R.; Peluso, S.; De Michele, G. Parkinsonism in a pair of monozygotic CADASIL twins sharing the R1006C mutation: a transcranial sonography study. *Neurological sciences : official journal of the Italian Neurological Society and of the Italian Society of Clinical Neurophysiology* **2016**, *37*, 875-881, doi:10.1007/s10072-016-2497-x.
133. Vauthier, V.; Jaillard, S.; Journel, H.; Dubourg, C.; Jockers, R.; Dam, J. Homozygous deletion of an 80 kb region comprising part of DNAJC6 and LEPR genes on chromosome 1P31.3 is associated with early onset obesity, mental retardation and epilepsy. *Molecular genetics and*

metabolism **2012**, *106*, 345-350, doi:10.1016/j.ymgme.2012.04.026.
134. Yu, X.; Yang, L.; Li, J.; Li, W.; Li, D.; Wang, R.; Wu, K.; Chen, W.; Zhang, Y.; Qiu, Z.; et al. De Novo and Inherited SETD1A Variants in Early-onset Epilepsy. *Neuroscience bulletin* **2019**, *35*, 1045-1057, doi:10.1007/s12264-019-00400-w.
135. Kummeling, J.; Stremmelaar, D.E.; Raun, N.; Reijnders, M.R.F.; Willemsen, M.H.; Ruiterkamp-Versteeg, M.; Schepens, M.; Man, C.C.O.; Gilissen, C.; Cho, M.T.; et al. Characterization of SETD1A haploinsufficiency in humans and Drosophila defines a novel neurodevelopmental syndrome. *Molecular psychiatry* **2021**, *26*, 2013-2024, doi:10.1038/s41380-020-0725-5.
136. Mei, D.; Cetica, V.; Marini, C.; Guerrini, R. Dravet syndrome as part of the clinical and genetic spectrum of sodium channel epilepsies and encephalopathies. *Epilepsia* **2019**, *60 Suppl 3*, S2-s7, doi:10.1111/epi.16054.
137. Borlot, F.; de Almeida, B.I.; Combe, S.L.; Andrade, D.M.; Filloux, F.M.; Myers, K.A. Clinical utility of multigene panel testing in adults with epilepsy and intellectual disability. *Epilepsia* **2019**, *60*, 1661-1669, doi:10.1111/epi.16273.
138. Rozycka, A.; Steinborn, B.; Trzeciak, W.H. The 1674+11C>T polymorphism of CHRNA4 is associated with juvenile myoclonic epilepsy. *Seizure* **2009**, *18*, 601-603, doi:10.1016/j.seizure.2009.06.007.
139. Chou, I.C.; Lee, C.C.; Huang, C.C.; Wu, J.Y.; Tsai, J.J.; Tsai, C.H.; Tsai, F.J. Association of the neuronal nicotinic acetylcholine receptor subunit alpha4 polymorphisms with febrile convulsions. *Epilepsia* **2003**, *44*, 1089-1093, doi:10.1046/j.1528-1157.2003.t01-1-44702.x.
140. Norberg, A.; Forsgren, L.; Holmberg, D.; Holmberg, M. Exclusion of the juvenile myoclonic epilepsy gene EFHC1 as the cause of migraine on chromosome 6, but association to two rare polymorphisms in MEP1A and RHAG. *Neuroscience letters* **2006**, *396*, 137-142, doi:10.1016/j.neulet.2005.11.039.
141. Suzuki, T.; Delgado-Escueta, A.V.; Aguan, K.; Alonso, M.E.; Shi, J.; Hara, Y.; Nishida, M.; Numata, T.; Medina, M.T.; Takeuchi, T.; et al. Mutations in EFHC1 cause juvenile myoclonic epilepsy. *Nature genetics* **2004**, *36*, 842-849, doi:10.1038/ng1393.
142. Chen, Y.; Wu, L.; Fang, Y.; He, Z.; Peng, B.; Shen, Y.; Xu, Q. A novel mutation of the nicotinic acetylcholine receptor gene CHRNA4 in sporadic nocturnal frontal lobe epilepsy. *Epilepsy Res* **2009**, *83*, 152-156, doi:10.1016/j.eplepsyres.2008.10.009.
143. Chen, Z.; Wang, L.; Wang, C.; Chen, Q.; Zhai, Q.; Guo, Y.; Zhang, Y. Mutational analysis of CHRNB2, CHRNA2 and CHRNA4 genes in Chinese population with autosomal dominant nocturnal frontal lobe epilepsy. *International journal of clinical and experimental medicine* **2015**, *8*, 9063-9070.
144. Gu, W.; Bertrand, D.; Steinlein, O.K. A major role of the nicotinic acetylcholine receptor gene CHRNA2 in autosomal dominant nocturnal frontal lobe epilepsy (ADNFLE) is unlikely. *Neuroscience letters* **2007**, *422*, 74-76, doi:10.1016/j.neulet.2007.06.006.
145. Uusimaa, J.; Gowda, V.; McShane, A.; Smith, C.; Evans, J.; Shrier, A.; Narasimhan, M.; O'Rourke, A.; Rajabally, Y.; Hedderly, T.; et al. Prospective study of POLG mutations presenting in children with intractable epilepsy: prevalence and clinical features. *Epilepsia* **2013**, *54*, 1002-1011, doi:10.1111/epi.12115.
146. Møller, R.S.; Weber, Y.G.; Klitten, L.L.; Trucks, H.; Muhle, H.; Kunz, W.S.; Mefford, H.C.; Franke, A.; Kautza, M.; Wolf, P.; et al. Exon-disrupting deletions of NRXN1 in idiopathic generalized epilepsy. *Epilepsia* **2013**, *54*, 256-264, doi:10.1111/epi.12078.

147. Friedman, J.I.; Vrijenhoek, T.; Markx, S.; Janssen, I.M.; van der Vliet, W.A.; Faas, B.H.; Knoers, N.V.; Cahn, W.; Kahn, R.S.; Edelmann, L.; et al. CNTNAP2 gene dosage variation is associated with schizophrenia and epilepsy. *Molecular psychiatry* **2008**, *13*, 261-266, doi:10.1038/sj.mp.4002049.
148. Mefford, H.C.; Muhle, H.; Ostertag, P.; von Spiczak, S.; Buysse, K.; Baker, C.; Franke, A.; Malafosse, A.; Genton, P.; Thomas, P.; et al. Genome-wide copy number variation in epilepsy: novel susceptibility loci in idiopathic generalized and focal epilepsies. *PLoS genetics* **2010**, *6*, e1000962, doi:10.1371/journal.pgen.1000962.
149. Singh, S.; Ganesh, S. Lafora progressive myoclonus epilepsy: a meta-analysis of reported mutations in the first decade following the discovery of the EPM2A and NHLRC1 genes. *Human mutation* **2009**, *30*, 715-723, doi:10.1002/humu.20954.
150. Sherr, E.H. The ARX story (epilepsy, mental retardation, autism, and cerebral malformations): one gene leads to many phenotypes. *Current opinion in pediatrics* **2003**, *15*, 567-571, doi:10.1097/00008480-200312000-00004.
151. Hirose, S.; Mitsudome, A. X-linked mental retardation and epilepsy: pathogenetic significance of ARX mutations. *Brain & development* **2003**, *25*, 161-165, doi:10.1016/s0387-7604(02)00169-9.
152. Guo, Y.; Baum, L.W.; Sham, P.C.; Wong, V.; Ng, P.W.; Lui, C.H.; Sin, N.C.; Tsoi, T.H.; Tang, C.S.; Kwan, J.S.; et al. Two-stage genome-wide association study identifies variants in CAMSAP1L1 as susceptibility loci for epilepsy in Chinese. *Human molecular genetics* **2012**, *21*, 1184-1189, doi:10.1093/hmg/ddr550.
153. Xiong, S.; Wang, Y.; Li, H.; Zhang, X. Interaction among GRIK2 gene on epilepsy susceptibility in Chinese children. *Acta neurologica Scandinavica* **2019**, *139*, 540-545, doi:10.1111/ane.13089.
154. Zweier, M.; Gregor, A.; Zweier, C.; Engels, H.; Sticht, H.; Wohlleber, E.; Bijlsma, E.K.; Holder, S.E.; Zenker, M.; Rossier, E.; et al. Mutations in MEF2C from the 5q14.3q15 microdeletion syndrome region are a frequent cause of severe mental retardation and diminish MECP2 and CDKL5 expression. *Human mutation* **2010**, *31*, 722-733, doi:10.1002/humu.21253.
155. Syrbe, S.; Hedrich, U.B.S.; Riesch, E.; Djémié, T.; Müller, S.; Møller, R.S.; Maher, B.; Hernandez-Hernandez, L.; Synofzik, M.; Caglayan, H.S.; et al. De novo loss- or gain-of-function mutations in KCNA2 cause epileptic encephalopathy. *Nature genetics* **2015**, *47*, 393-399, doi:10.1038/ng.3239.
156. Neubauer, B.A.; Waldegger, S.; Heinzinger, J.; Hahn, A.; Kurlemann, G.; Fiedler, B.; Eberhard, F.; Muhle, H.; Stephani, U.; Garkisch, S.; et al. KCNQ2 and KCNQ3 mutations contribute to different idiopathic epilepsy syndromes. *Neurology* **2008**, *71*, 177-183, doi:10.1212/01.wnl.0000317090.92185.ec.
157. Tincheva, S.; Todorov, T.; Todorova, A.; Georgieva, R.; Stamatov, D.; Yordanova, I.; Kadiyska, T.; Georgieva, B.; Bojidarova, M.; Tacheva, G.; et al. First cases of pyridoxine-dependent epilepsy in Bulgaria: novel mutation in the ALDH7A1 gene. *Neurological sciences : official journal of the Italian Neurological Society and of the Italian Society of Clinical Neurophysiology* **2015**, *36*, 2209-2212, doi:10.1007/s10072-015-2338-3.
158. Salpietro, V.; Dixon, C.L.; Guo, H.; Bello, O.D.; Vandrovcova, J.; Efthymiou, S.; Maroofian, R.; Heimer, G.; Burglen, L.; Valence, S.; et al. AMPA receptor GluA2 subunit defects are a cause of neurodevelopmental disorders. *Nature communications* **2019**, *10*, 3094, doi:10.1038/s41467-019-10910-w.

159. Carvill, G.L.; Regan, B.M.; Yendle, S.C.; O'Roak, B.J.; Lozovaya, N.; Bruneau, N.; Burnashev, N.; Khan, A.; Cook, J.; Geraghty, E.; et al. GRIN2A mutations cause epilepsy-aphasia spectrum disorders. *Nature genetics* **2013**, *45*, 1073-1076, doi:10.1038/ng.2727.

160. Mumoli, L.; Tarantino, P.; Michelucci, R.; Bianchi, A.; Labate, A.; Franceschetti, S.; Marini, C.; Striano, P.; Gagliardi, M.; Ferlazzo, E.; et al. No evidence of a role for cystatin B gene in juvenile myoclonic epilepsy. *Epilepsia* **2015**, *56*, e40-43, doi:10.1111/epi.12944.

161. Di Matteo, F.; Pipicelli, F.; Kyrousi, C.; Tovecci, I.; Penna, E.; Crispino, M.; Chambery, A.; Russo, R.; Ayo-Martin, A.C.; Giordano, M.; et al. Cystatin B is essential for proliferation and interneuron migration in individuals with EPM1 epilepsy. *EMBO molecular medicine* **2020**, *12*(6):e11419, doi:10.15252/emmm.201911419.

162. Segal, E.; Pedro, H.; Valdez-Gonzalez, K.; Parisotto, S.; Gliksman, F.; Thompson, S.; Sabri, J.; Fertig, E. Diagnostic Yield of Epilepsy Panels in Children With Medication-Refractory Epilepsy. *Pediatric neurology* **2016**, *64*, 66-71, doi:10.1016/j.pediatrneurol.2016.06.019.

163. Busolin, G.; Malacrida, S.; Bisulli, F.; Striano, P.; Di Bonaventura, C.; Egeo, G.; Pasini, E.; Cianci, V.; Ferlazzo, E.; Bianchi, A.; et al. Association of intronic variants of the KCNAB1 gene with lateral temporal epilepsy. *Epilepsy Res* **2011**, *94*, 110-116, doi:10.1016/j.eplepsyres.2011.01.010.

164. Cavalleri, G.L.; Weale, M.E.; Shianna, K.V.; Singh, R.; Lynch, J.M.; Grinton, B.; Szoeke, C.; Murphy, K.; Kinirons, P.; O'Rourke, D.; et al. Multicentre search for genetic susceptibility loci in sporadic epilepsy syndrome and seizure types: a case-control study. *The Lancet. Neurology* **2007**, *6*, 970-980, doi:10.1016/s1474-4422(07)70247-8.

165. Neuhofer, C.M.; Funke, R.; Wilken, B.; Knaus, A.; Altmüller, J.; Nürnberg, P.; Li, Y.; Wollnik, B.; Burfeind, P.; Pauli, S. A Novel Mutation in PIGA Associated with Multiple Congenital Anomalies-Hypotonia-Seizure Syndrome 2 (MCAHS2) in a Boy with a Combination of Severe Epilepsy and Gingival Hyperplasia. *Molecular syndromology* **2020**, *11*, 30-37, doi:10.1159/000505797.

166. Wang, S.; Wang, D.; Cai, X.; Wu, Q.; Han, Y. Identification of the ZEB2 gene as a potential target for epilepsy therapy and the association between rs10496964 and ZEB2 expression. *The Journal of international medical research* **2020**, *48*(12):300060520980527, doi:10.1177/0300060520980527.

167. Symonds, J.D.; Joss, S.; Metcalfe, K.A.; Somarathi, S.; Cruden, J.; Devlin, A.M.; Donaldson, A.; DiDonato, N.; Fitzpatrick, D.; Kaiser, F.J.; et al. Heterozygous truncation mutations of the SMC1A gene cause a severe early onset epilepsy with cluster seizures in females: Detailed phenotyping of 10 new cases. *Epilepsia* **2017**, *58*, 565-575, doi:10.1111/epi.13669.

168. Tanaka, M.; Bailey, J.N.; Bai, D.; Ishikawa-Brush, Y.; Delgado-Escueta, A.V.; Olsen, R.W. Effects on promoter activity of common SNPs in 5' region of GABRB3 exon 1A. *Epilepsia* **2012**, *53*, 1450-1456, doi:10.1111/j.1528-1167.2012.03572.x.

169. Ohmori, I.; Ouchida, M.; Miki, T.; Mimaki, N.; Kiyonaka, S.; Nishiki, T.; Tomizawa, K.; Mori, Y.; Matsui, H. A CACNB4 mutation shows that altered Ca(v)2.1 function may be a genetic modifier of severe myoclonic epilepsy in infancy. *Neurobiology of disease* **2008**, *32*, 349-354, doi:10.1016/j.nbd.2008.07.017.

170. Balciuniene, J.; DeChene, E.T.; Akgumus, G.; Romasko, E.J.; Cao, K.; Dubbs, H.A.; Mulchandani, S.; Spinner, N.B.; Conlin, L.K.; Marsh, E.D.; et al. Use of a Dynamic Genetic Testing Approach for Childhood-Onset Epilepsy. *JAMA network open* **2019**, *2*(4):e192129, doi:10.1001/jamanetworkopen.2019.2129.

171. Hino-Fukuyo, N.; Kikuchi, A.; Arai-Ichinoi, N.; Niihori, T.; Sato, R.; Suzuki, T.; Kudo, H.; Sato, Y.; Nakayama, T.; Kakisaka, Y.; et al. Genomic analysis identifies candidate pathogenic variants in 9 of 18 patients with unexplained West syndrome. *Human genetics* **2015**, *134*, 649-658, doi:10.1007/s00439-015-1553-6.
172. Wallace, R.H.; Freeman, J.L.; Shouri, M.R.; Izzillo, P.A.; Rosenfeld, J.V.; Mulley, J.C.; Harvey, A.S.; Berkovic, S.F. Somatic mutations in GLI3 can cause hypothalamic hamartoma and gelastic seizures. *Neurology* **2008**, *70*, 653-655, doi:10.1212/01.wnl.0000284607.12906.c5.
173. Lorenz, S.; Heils, A.; Kasper, J.M.; Sander, T. Allelic association of a truncation mutation of the KCNMB3 gene with idiopathic generalized epilepsy. *American journal of medical genetics. Part B, Neuropsychiatric genetics : the official publication of the International Society of Psychiatric Genetics* **2007**, *144b*, 10-13, doi:10.1002/ajmg.b.30369.
174. Wang, Y.; Du, X.; Bin, R.; Yu, S.; Xia, Z.; Zheng, G.; Zhong, J.; Zhang, Y.; Jiang, Y.H.; Wang, Y. Genetic Variants Identified from Epilepsy of Unknown Etiology in Chinese Children by Targeted Exome Sequencing. *Scientific reports* **2017**, *7*, 40319, doi:10.1038/srep40319.
175. Schorling, D.C.; Rost, S.; Lefeber, D.J.; Brady, L.; Müller, C.R.; Korinthenberg, R.; Tarnopolsky, M.; Bönnemann, C.G.; Rodenburg, R.J.; Bugiani, M.; et al. Early and lethal neurodegeneration with myasthenic and myopathic features: A new ALG14-CDG. *Neurology* **2017**, *89*, 657-664, doi:10.1212/wnl.0000000000004234.
176. Mao, X.; Bruneau, N.; Gao, Q.; Becq, H.; Jia, Z.; Xi, H.; Shu, L.; Wang, H.; Szepetowski, P.; Aniksztejn, L. The Epilepsy of Infancy With Migrating Focal Seizures: Identification of de novo Mutations of the KCNT2 Gene That Exert Inhibitory Effects on the Corresponding Heteromeric K(Na)1.1/K(Na)1.2 Potassium Channel. *Frontiers in cellular neuroscience* **2020**, *14*, 1, doi:10.3389/fncel.2020.00001.
177. Segel, R.; Aran, A.; Gulsuner, S.; Nakamura, H.; Rosen, T.; Walsh, T.; Denda, H.; Zeligson, S.; Eto, K.; Beeri, R.; et al. A defect in GPI synthesis as a suggested mechanism for the role of ARV1 in intellectual disability and seizures. *Neurogenetics* **2020**, *21*, 259-267, doi:10.1007/s10048-020-00615-4.
178. Perenthaler, E.; Nikoncuk, A.; Yousefi, S.; Berdowski, W.M.; Alsagob, M.; Capo, I.; van der Linde, H.C.; van den Berg, P.; Jacobs, E.H.; Putar, D.; et al. Loss of UGP2 in brain leads to a severe epileptic encephalopathy, emphasizing that bi-allelic isoform-specific start-loss mutations of essential genes can cause genetic diseases. *Acta neuropathologica* **2020**, *139*, 415-442, doi:10.1007/s00401-019-02109-6.
179. Gilsoul, M.; Grisar, T.; Delgado-Escueta, A.V.; de Nijs, L.; Lakaye, B. Subtle Brain Developmental Abnormalities in the Pathogenesis of Juvenile Myoclonic Epilepsy. *Frontiers in cellular neuroscience* **2019**, *13*, 433, doi:10.3389/fncel.2019.00433.
180. Alfaiz, A.A.; Müller, V.; Boutry-Kryza, N.; Ville, D.; Guex, N.; de Bellescize, J.; Rivier, C.; Labalme, A.; des Portes, V.; Edery, P.; et al. West syndrome caused by homozygous variant in the evolutionary conserved gene encoding the mitochondrial elongation factor GUF1. *European journal of human genetics : EJHG* **2016**, *24*, 1001-1008, doi:10.1038/ejhg.2015.227.
181. Turnbull, J.; Girard, J.M.; Lohi, H.; Chan, E.M.; Wang, P.; Tiberia, E.; Omer, S.; Ahmed, M.; Bennett, C.; Chakrabarty, A.; et al. Early-onset Lafora body disease. *Brain : a journal of neurology* **2012**, *135*, 2684-2698, doi:10.1093/brain/aws205.
182. Guella, I.; McKenzie, M.B.; Evans, D.M.; Buerki, S.E.; Toyota, E.B.; Van Allen, M.I.; Suri, M.; Elmslie, F.; Simon, M.E.H.; van Gassen, K.L.I.; et al. De Novo Mutations in YWHAG Cause Early-

Onset Epilepsy. *American journal of human genetics* **2017**, *101*, 300-310, doi:10.1016/j.ajhg.2017.07.004.

183. Elsea, S.H.; Solyom, A.; Martin, K.; Harmatz, P.; Mitchell, J.; Lampe, C.; Grant, C.; Selim, L.; Mungan, N.O.; Guelbert, N.; et al. ASAH1 pathogenic variants associated with acid ceramidase deficiency: Farber disease and spinal muscular atrophy with progressive myoclonic epilepsy. *Human mutation* **2020**, *41*, 1469-1487, doi:10.1002/humu.24056.

184. Alsahli, S.; Al-Twaijri, W.; Al Mutairi, F. Confirming the pathogenicity of NECAP1 in early onset epileptic encephalopathy. *Epilepsia open* **2018**, *3*, 524-527, doi:10.1002/epi4.12263.

185. Johnstone, D.L.; Nguyen, T.T.M.; Zambonin, J.; Kernohan, K.D.; St-Denis, A.; Baratang, N.V.; Hartley, T.; Geraghty, M.T.; Richer, J.; Majewski, J.; et al. Early infantile epileptic encephalopathy due to biallelic pathogenic variants in PIGQ: Report of seven new subjects and review of the literature. *Journal of inherited metabolic disease* **2020**, *43*, 1321-1332, doi:10.1002/jimd.12278.

186. Yang, Q.Z.; Spelbrink, E.M.; Nye, K.L.; Hsu, E.R.; Porter, B.E. Epilepsy and EEG Phenotype of SLC13A5 Citrate Transporter Disorder. *Child neurology open* **2020**, *7*:2329048x20931361, doi:10.1177/2329048x20931361.

187. Jepson, J.E.C.; Praschberger, R.; Krishnakumar, S.S. Mechanisms of Neurological Dysfunction in GOSR2 Progressive Myoclonus Epilepsy, a Golgi SNAREopathy. *Neuroscience* **2019**, *420*, 41-49, doi:10.1016/j.neuroscience.2019.03.057.

188. Soleimanipour, F.; Razmara, E.; Rahbarizadeh, F.; Fallahi, E.; Khodaeian, M.; Tavasoli, A.R.; Garshasbi, M. A novel missense variant in the LMNB2 gene causes progressive myoclonus epilepsy. *Acta neurologica Belgica* **2022**, *122*, 659-667, doi:10.1007/s13760-021-01650-0.

189. Bar, C.; Barcia, G.; Jennesson, M.; Le Guyader, G.; Schneider, A.; Mignot, C.; Lesca, G.; Breuillard, D.; Montomoli, M.; Keren, B.; et al. Expanding the genetic and phenotypic relevance of KCNB1 variants in developmental and epileptic encephalopathies: 27 new patients and overview of the literature. *Human mutation* **2020**, *41*, 69-80, doi:10.1002/humu.23915.

190. Krenn, M.; Knaus, A.; Westphal, D.S.; Wortmann, S.B.; Polster, T.; Woermann, F.G.; Karenfort, M.; Mayatepek, E.; Meitinger, T.; Wagner, M.; et al. Biallelic mutations in PIGP cause developmental and epileptic encephalopathy. *Annals of clinical and translational neurology* **2019**, *6*, 968-973, doi:10.1002/acn3.768.

191. Ranta, S.; Savukoski, M.; Santavuori, P.; Haltia, M. Studies of homogenous populations: CLN5 and CLN8. *Advances in genetics* **2001**, *45*, 123-140, doi:10.1016/s0065-2660(01)45007-3.

192. Willemsen, M.H.; Goel, H.; Verhoeven, J.S.; Braakman, H.M.H.; de Leeuw, N.; Freeth, A.; Minassian, B.A. Epilepsy phenotype in individuals with chromosomal duplication encompassing FGF12. *Epilepsia open* **2020**, *5*, 301-306, doi:10.1002/epi4.12396.

193. Mizuguchi, T.; Nakashima, M.; Kato, M.; Okamoto, N.; Kurahashi, H.; Ekhilevitch, N.; Shiina, M.; Nishimura, G.; Shibata, T.; Matsuo, M.; et al. Loss-of-function and gain-of-function mutations in PPP3CA cause two distinct disorders. *Human molecular genetics* **2018**, *27*, 1421-1433, doi:10.1093/hmg/ddy052.

194. Lewis, H.; Samanta, D.; Örsell, J.L.; Bosanko, K.A.; Rowell, A.; Jones, M.; Dale, R.C.; Taravath, S.; Hahn, C.D.; Krishnakumar, D.; et al. Epilepsy and Electroencephalographic Abnormalities in SATB2-Associated Syndrome. *Pediatric neurology* **2020**, *112*, 94-100, doi:10.1016/j.pediatrneurol.2020.04.006.

195. Fatima, A.; Hoeber, J.; Schuster, J.; Koshimizu, E.; Maya-Gonzalez, C.; Keren, B.; Mignot, C.; Akram, T.; Ali, Z.; Miyatake, S.; et al. Monoallelic and bi-allelic variants in NCDN cause

neurodevelopmental delay, intellectual disability, and epilepsy. *American journal of human genetics* **2021**, *108*, 739-748, doi:10.1016/j.ajhg.2021.02.015.
196. Chaudhuri, T.; Chintalapati, J.; Hosur, M.V. Identification of 3'-UTR single nucleotide variants and prediction of select protein imbalance in mesial temporal lobe epilepsy patients. *PloS one* **2021**, *16*(6):e0252475, doi:10.1371/journal.pone.0252475.
197. Marafi, D.; Fatih, J.M.; Kaiyrzhanov, R.; Ferla, M.P.; Gijavanekar, C.; Al-Maraghi, A.; Liu, N.; Sites, E.; Alsaif, H.S.; Al-Owain, M.; et al. Biallelic variants in SLC38A3 encoding a glutamine transporter cause epileptic encephalopathy. *Brain : a journal of neurology* **2022**, *145*, 909-924, doi:10.1093/brain/awab369.
198. De Franco, E.; Lytrivi, M.; Ibrahim, H.; Montaser, H.; Wakeling, M.N.; Fantuzzi, F.; Patel, K.; Demarez, C.; Cai, Y.; Igoillo-Esteve, M.; et al. YIPF5 mutations cause neonatal diabetes and microcephaly through endoplasmic reticulum stress. *The Journal of clinical investigation* **2020**, *130*, 6338-6353, doi:10.1172/jci141455.
199. Lionel, A.C.; Monfared, N.; Scherer, S.W.; Marshall, C.R.; Mercimek-Mahmutoglu, S. MED23-associated refractory epilepsy successfully treated with the ketogenic diet. *American journal of medical genetics. Part A* **2016**, *170*, 2421-2425, doi:10.1002/ajmg.a.37802.
200. Ohba, C.; Shiina, M.; Tohyama, J.; Haginoya, K.; Lerman-Sagie, T.; Okamoto, N.; Blumkin, L.; Lev, D.; Mukaida, S.; Nozaki, F.; et al. GRIN1 mutations cause encephalopathy with infantile-onset epilepsy, and hyperkinetic and stereotyped movement disorders. *Epilepsia* **2015**, *56*, 841-848, doi:10.1111/epi.12987.
201. Itai, T.; Hamanaka, K.; Sasaki, K.; Wagner, M.; Kotzaeridou, U.; Brösse, I.; Ries, M.; Kobayashi, Y.; Tohyama, J.; Kato, M.; et al. De novo variants in CELF2 that disrupt the nuclear localization signal cause developmental and epileptic encephalopathy. *Human mutation* **2021**, *42*, 66-76, doi:10.1002/humu.24130.
202. Schwarz, N.; Seiffert, S.; Pendziwiat, M.; Rademacher, A.V.; Brünger, T.; Hedrich, U.B.S.; Augustijn, P.B.; Baier, H.; Bayat, A.; Bisulli, F.; et al. Spectrum of Phenotypic, Genetic, and Functional Characteristics in Patients With Epilepsy With KCNC2 Pathogenic Variants. *Neurology* **2022**, *98*, e2046-e2059, doi:10.1212/wnl.0000000000200660.
203. Campostrini, G.; DiFrancesco, J.C.; Castellotti, B.; Milanesi, R.; Gnecchi-Ruscone, T.; Bonzanni, M.; Bucchi, A.; Baruscotti, M.; Ferrarese, C.; Franceschetti, S.; et al. A Loss-of-Function HCN4 Mutation Associated With Familial Benign Myoclonic Epilepsy in Infancy Causes Increased Neuronal Excitability. *Frontiers in molecular neuroscience* **2018**, *11*, 269, doi:10.3389/fnmol.2018.00269.
204. Efthymiou, S.; Dutra-Clarke, M.; Maroofian, R.; Kaiyrzhanov, R.; Scala, M.; Reza Alvi, J.; Sultan, T.; Christoforou, M.; Tuyet Mai Nguyen, T.; Mankad, K.; et al. Expanding the phenotype of PIGS-associated early onset epileptic developmental encephalopathy. *Epilepsia* **2021**, *62*, e35-e41, doi:10.1111/epi.16801.
205. Bott, L.C.; Forouhan, M.; Lieto, M.; Sala, A.J.; Ellerington, R.; Johnson, J.O.; Speciale, A.A.; Criscuolo, C.; Filla, A.; Chitayat, D.; et al. Variants in ATP6V0A1 cause progressive myoclonus epilepsy and developmental and epileptic encephalopathy. *Brain communications* **2021**, *3*, fcab245, doi:10.1093/braincomms/fcab245.
206. Paulhus, K.; Ammerman, L.; Glasscock, E. Clinical Spectrum of KCNA1 Mutations: New Insights into Episodic Ataxia and Epilepsy Comorbidity. *International journal of molecular sciences* **2020**, *21*(8):2802, doi:10.3390/ijms21082802.

207. Leng, X.; Zhang, T.; Guan, Y.; Tang, M. Genotype and phenotype analysis of epilepsy caused by ADGRV1 mutations in Chinese children. *Seizure* **2022**, *103*, 108-114, doi:https://doi.org/10.1016/j.seizure.2022.11.005.
208. Poeta, L.; Fusco, F.; Drongitis, D.; Shoubridge, C.; Manganelli, G.; Filosa, S.; Paciolla, M.; Courtney, M.; Collombat, P.; Lioi, M.B.; et al. A regulatory path associated with X-linked intellectual disability and epilepsy links KDM5C to the polyalanine expansions in ARX. *American journal of human genetics* **2013**, *92*, 114-125, doi:10.1016/j.ajhg.2012.11.008.
209. Puffenberger, E.G.; Jinks, R.N.; Sougnez, C.; Cibulskis, K.; Willert, R.A.; Achilly, N.P.; Cassidy, R.P.; Fiorentini, C.J.; Heiken, K.F.; Lawrence, J.J.; et al. Genetic mapping and exome sequencing identify variants associated with five novel diseases. *PloS one* **2012**, *7*(1):e28936, doi:10.1371/journal.pone.0028936.
210. Broeks, M.H.; Shamseldin, H.E.; Alhashem, A.; Hashem, M.; Abdulwahab, F.; Alshedi, T.; Alobaid, I.; Zwartkruis, F.; Westland, D.; Fuchs, S.; et al. MDH1 deficiency is a metabolic disorder of the malate-aspartate shuttle associated with early onset severe encephalopathy. *Human genetics* **2019**, *138*, 1247-1257, doi:10.1007/s00439-019-02063-z.
211. Corbett, M.A.; Kroes, T.; Veneziano, L.; Bennett, M.F.; Florian, R.; Schneider, A.L.; Coppola, A.; Licchetta, L.; Franceschetti, S.; Suppa, A.; et al. Intronic ATTTC repeat expansions in STARD7 in familial adult myoclonic epilepsy linked to chromosome 2. *Nature communications* **2019**, *10*, 4920, doi:10.1038/s41467-019-12671-y.
212. Todd, B.P.; Bassuk, A.G. A de novo mutation in PRICKLE1 associated with myoclonic epilepsy and autism spectrum disorder. *Journal of neurogenetics* **2018**, *32*, 313-315, doi:10.1080/01677063.2018.1473862.
213. Combi, R.; Dalprà, L.; Malcovati, M.; Oldani, A.; Tenchini, M.L.; Ferini-Strambi, L. Evidence for a fourth locus for autosomal dominant nocturnal frontal lobe epilepsy. *Brain research bulletin* **2004**, *63*, 353-359, doi:10.1016/j.brainresbull.2003.12.007.
214. Feng, H.; Sjögren, B.; Karaj, B.; Shaw, V.; Gezer, A.; Neubig, R.R. Movement disorder in GNAO1 encephalopathy associated with gain-of-function mutations. *Neurology* **2017**, *89*, 762-770, doi:10.1212/wnl.0000000000004262.
215. Mazzola, L.; Oliver, K.L.; Labalme, A.; Baykan, B.; Muona, M.; Joensuu, T.H.; Courage, C.; Chatron, N.; Borsani, G.; Alix, E.; et al. Progressive Myoclonus Epilepsy Caused by a Homozygous Splicing Variant of SLC7A6OS. *Annals of neurology* **2021**, *89*, 402-407, doi:10.1002/ana.25941.
216. Abdel-Salam, G.M.; Schaffer, A.E.; Zaki, M.S.; Dixon-Salazar, T.; Mostafa, I.S.; Afifi, H.H.; Gleeson, J.G. A homozygous IER3IP1 mutation causes microcephaly with simplified gyral pattern, epilepsy, and permanent neonatal diabetes syndrome (MEDS). *American journal of medical genetics. Part A* **2012**, *158a*, 2788-2796, doi:10.1002/ajmg.a.35583.
217. Manole, A.; Efthymiou, S.; O'Connor, E.; Mendes, M.I.; Jennings, M.; Maroofian, R.; Davagnanam, I.; Mankad, K.; Lopez, M.R.; Salpietro, V.; et al. De Novo and Bi-allelic Pathogenic Variants in NARS1 Cause Neurodevelopmental Delay Due to Toxic Gain-of-Function and Partial Loss-of-Function Effects. *American journal of human genetics* **2020**, *107*, 311-324, doi:10.1016/j.ajhg.2020.06.016.
218. Godeiro Junior, C.d.O.; Vale, T.C.; Afonso, C.O.d.M.; Kok, F.; Pedroso, J.L.; Barsottini, O.G. Progressive Myoclonic Epilepsy Type 8 Due to CERS1 Deficiency: A Novel Mutation with Prominent Ataxia. *Movement Disorders Clinical Practice* **2018**, *5*, 330-332,

doi:https://doi.org/10.1002/mdc3.12610.
219. XiangWei, W.; Kannan, V.; Xu, Y.; Kosobucki, G.J.; Schulien, A.J.; Kusumoto, H.; Moufawad El Achkar, C.; Bhattacharya, S.; Lesca, G.; Nguyen, S.; et al. Heterogeneous clinical and functional features of GRIN2D-related developmental and epileptic encephalopathy. *Brain : a journal of neurology* **2019**, *142*, 3009-3027, doi:10.1093/brain/awz232.
220. Puranam, R.S.; He, X.P.; Yao, L.; Le, T.; Jang, W.; Rehder, C.W.; Lewis, D.V.; McNamara, J.O. Disruption of Fgf13 causes synaptic excitatory-inhibitory imbalance and genetic epilepsy and febrile seizures plus. *The Journal of neuroscience : the official journal of the Society for Neuroscience* **2015**, *35*, 8866-8881, doi:10.1523/jneurosci.3470-14.2015.
221. Huo, J.; Ren, S.; Gao, P.; Wan, D.; Rong, S.; Li, X.; Liu, S.; Xu, S.; Sun, K.; Guo, B.; et al. ALG13 participates in epileptogenesis via regulation of GABA(A) receptors in mouse models. *Cell death discovery* **2020**, *6*, 87, doi:10.1038/s41420-020-00319-6.
222. Gao, P.; Wang, F.; Huo, J.; Wan, D.; Zhang, J.; Niu, J.; Wu, J.; Yu, B.; Sun, T. ALG13 Deficiency Associated with Increased Seizure Susceptibility and Severity. *Neuroscience* **2019**, *409*, 204-221, doi:10.1016/j.neuroscience.2019.03.009.
223. Mulhern, M.S.; Stumpel, C.; Stong, N.; Brunner, H.G.; Bier, L.; Lippa, N.; Riviello, J.; Rouhl, R.P.W.; Kempers, M.; Pfundt, R.; et al. NBEA: Developmental disease gene with early generalized epilepsy phenotypes. *Annals of neurology* **2018**, *84*, 788-795, doi:10.1002/ana.25350.
224. Alevy, J.; Burger, C.A.; Albrecht, N.E.; Jiang, D.; Samuel, M.A. Progressive myoclonic epilepsy-associated gene Kctd7 regulates retinal neurovascular patterning and function. *Neurochemistry international* **2019**, *129*, 104486, doi:10.1016/j.neuint.2019.104486.
225. Everett, K.V.; Chioza, B.; Aicardi, J.; Aschauer, H.; Brouwer, O.; Callenbach, P.; Covanis, A.; Dulac, O.; Eeg-Olofsson, O.; Feucht, M.; et al. Linkage and association analysis of CACNG3 in childhood absence epilepsy. *European journal of human genetics : EJHG* **2007**, *15*, 463-472, doi:10.1038/sj.ejhg.5201783.
226. Panjwani, N.; Wilson, M.D.; Addis, L.; Crosbie, J.; Wirrell, E.; Auvin, S.; Caraballo, R.H.; Kinali, M.; McCormick, D.; Oren, C.; et al. A microRNA-328 binding site in PAX6 is associated with centrotemporal spikes of rolandic epilepsy. *Annals of clinical and translational neurology* **2016**, *3*, 512-522, doi:10.1002/acn3.320.
227. Vlaskamp, D.R.M.; Callenbach, P.M.C.; Rump, P.; Giannini, L.A.A.; Dijkhuizen, T.; Brouwer, O.F.; van Ravenswaaij-Arts, C.M.A. Copy number variation in a hospital-based cohort of children with epilepsy. *Epilepsia open* **2017**, *2*, 244-254, doi:10.1002/epi4.12057.
228. Teng, F.Y.; Tang, B.L. Nogo signaling and non-physical injury-induced nervous system pathology. *Journal of neuroscience research* **2005**, *79*, 273-278, doi:10.1002/jnr.20361.
229. Thomas, R.A.; Ambalavanan, A.; Rouleau, G.A.; Barker, P.A. Identification of genetic variants of LGI1 and RTN4R (NgR1) linked to schizophrenia that are defective in NgR1-LGI1 signaling. *Molecular genetics & genomic medicine* **2016**, *4*, 447-456, doi:10.1002/mgg3.215.
230. Hata, Y.; Yoshida, K.; Kinoshita, K.; Nishida, N. Epilepsy-related sudden unexpected death: targeted molecular analysis of inherited heart disease genes using next-generation DNA sequencing. *Brain pathology (Zurich, Switzerland)* **2017**, *27*, 292-304, doi:10.1111/bpa.12390.
231. Soh, M.S.; Bagnall, R.D.; Bennett, M.F.; Bleakley, L.E.; Mohamed Syazwan, E.S.; Phillips, A.M.; Chiam, M.D.F.; McKenzie, C.E.; Hildebrand, M.; Crompton, D.; et al. Loss-of-function variants in K(v) 11.1 cardiac channels as a biomarker for SUDEP. *Annals of clinical and translational neurology* **2021**, *8*, 1422-1432, doi:10.1002/acn3.51381.

232. Hamdan, F.F.; Piton, A.; Gauthier, J.; Lortie, A.; Dubeau, F.; Dobrzeniecka, S.; Spiegelman, D.; Noreau, A.; Pellerin, S.; Côté, M.; et al. De novo STXBP1 mutations in mental retardation and nonsyndromic epilepsy. *Annals of neurology* **2009**, *65*, 748-753, doi:10.1002/ana.21625.
233. Harper, C.B.; Small, C.; Davenport, E.C.; Low, D.W.; Smillie, K.J.; Martínez-Mármol, R.; Meunier, F.A.; Cousin, M.A. An Epilepsy-Associated SV2A Mutation Disrupts Synaptotagmin-1 Expression and Activity-Dependent Trafficking. *The Journal of neuroscience : the official journal of the Society for Neuroscience* **2020**, *40*, 4586-4595, doi:10.1523/jneurosci.0210-20.2020.
234. Zhou, P.; He, N.; Zhang, J.W.; Lin, Z.J.; Wang, J.; Yan, L.M.; Meng, H.; Tang, B.; Li, B.M.; Liu, X.R.; et al. Novel mutations and phenotypes of epilepsy-associated genes in epileptic encephalopathies. *Genes, brain, and behavior* **2018**, *17*(8):e12456, doi:10.1111/gbb.12456.
235. Lal, D.; Pernhorst, K.; Klein, K.M.; Reif, P.; Tozzi, R.; Toliat, M.R.; Winterer, G.; Neubauer, B.; Nü rnberg, P.; Rosenow, F.; et al. Extending the phenotypic spectrum of RBFOX1 deletions: Sporadic focal epilepsy. *Epilepsia* **2015**, *56*, e129-133, doi:10.1111/epi.13076.
236. Winawer, M.R.; Griffin, N.G.; Samanamud, J.; Baugh, E.H.; Rathakrishnan, D.; Ramalingam, S.; Zagzag, D.; Schevon, C.A.; Dugan, P.; Hegde, M.; et al. Somatic SLC35A2 variants in the brain are associated with intractable neocortical epilepsy. *Annals of neurology* **2018**, *83*, 1133-1146, doi:10.1002/ana.25243.
237. Zhang, B.; Chen, M.; Yang, H.; Wu, T.; Song, C.; Guo, R. Evidence for involvement of the CD40/CD40L system in post-stroke epilepsy. *Neuroscience letters* **2014**, *567*, 6-10, doi:10.1016/j.neulet.2014.03.003.
238. Markus, F.; Angelini, C.; Trimouille, A.; Rudolf, G.; Lesca, G.; Goizet, C.; Lasseaux, E.; Arveiler, B.; van Slegtenhorst, M.; Brooks, A.S.; et al. Rare variants in the GABA(A) receptor subunit ε identified in patients with a wide spectrum of epileptic phenotypes. *Molecular genetics & genomic medicine* **2020**, *8*(9):e1388, doi:10.1002/mgg3.1388.
239. Henske, E.P.; Jóźwiak, S.; Kingswood, J.C.; Sampson, J.R.; Thiele, E.A. Tuberous sclerosis complex. *Nature reviews. Disease primers* **2016**, *2*, 16035, doi:10.1038/nrdp.2016.35.
240. Al-Yahyaee, S.; Al-Gazali, L.I.; De Jonghe, P.; Al-Barwany, H.; Al-Kindi, M.; De Vriendt, E.; Chand, P.; Koul, R.; Jacob, P.C.; Gururaj, A.; et al. A novel locus for hereditary spastic paraplegia with thin corpus callosum and epilepsy. *Neurology* **2006**, *66*, 1230-1234, doi:10.1212/01.wnl.0000208501.52849.dd.
241. Pensato, V.; Castellotti, B.; Gellera, C.; Pareyson, D.; Ciano, C.; Nanetti, L.; Salsano, E.; Piscosquito, G.; Sarto, E.; Eoli, M.; et al. Overlapping phenotypes in complex spastic paraplegias SPG11, SPG15, SPG35 and SPG48. *Brain : a journal of neurology* **2014**, *137*, 1907-1920, doi:10.1093/brain/awu121.
242. Gasser, M.; Boonsimma, P.; Netbaramee, W.; Wechapinan, T.; Srichomthomg, C.; Ittiwut, C.; Krenn, M.; Zimprich, F.; Milenkovic, I.; Abicht, A.; et al. ATP1A3-related epilepsy: Report of seven cases and literature-based analysis of treatment response. *Journal of clinical neuroscience : official journal of the Neurosurgical Society of Australasia* **2020**, *72*, 31-38, doi:10.1016/j.jocn.2020.01.041.
243. Licchetta, L.; Ferri, L.; La Morgia, C.; Zenesini, C.; Caporali, L.; Lucia Valentino, M.; Minardi, R.; Fulitano, D.; Di Vito, L.; Mostacci, B.; et al. Epilepsy in MT-ATP6 - related mils/NARP: correlation of elettroclinical features with heteroplasmy. *Annals of clinical and translational neurology* **2021**, *8*, 704-710, doi:10.1002/acn3.51259.
244. Karan, K.R.; Satishchandra, P.; Sinha, S.; Anand, A. Rare SLC1A1 variants in hot water epilepsy.

Human genetics **2017**, *136*, 693-703, doi:10.1007/s00439-017-1778-7.
245. Joshi, S.; Roden, W.H.; Kapur, J.; Jansen, L.A. Reduced neurosteroid potentiation of GABA(A) receptors in epilepsy and depolarized hippocampal neurons. *Annals of clinical and translational neurology* **2020**, *7*, 527-542, doi:10.1002/acn3.51023.
246. Olson, H.E.; Kelly, M.; LaCoursiere, C.M.; Pinsky, R.; Tambunan, D.; Shain, C.; Ramgopal, S.; Takeoka, M.; Libenson, M.H.; Julich, K.; et al. Genetics and genotype-phenotype correlations in early onset epileptic encephalopathy with burst suppression. *Annals of neurology* **2017**, *81*, 419-429, doi:10.1002/ana.24883.
247. Wilkie, H.; Osei-Lah, A.; Chioza, B.; Nashef, L.; McCormick, D.; Asherson, P.; Makoff, A.J. Association of mu-opioid receptor subunit gene and idiopathic generalized epilepsy. *Neurology* **2002**, *59*, 724-728, doi:10.1212/wnl.59.5.724.
248. DiBacco, M.L.; Pop, A.; Salomons, G.S.; Hanson, E.; Roullet, J.B.; Gibson, K.M.; Pearl, P.L. Novel ALDH5A1 variants and genotype: Phenotype correlation in SSADH deficiency. *Neurology* **2020**, *95*, e2675-e2682, doi:10.1212/wnl.0000000000010730.
249. Chen, L.; Zhu, L.; Lu, D.; Wu, Z.; Han, Y.; Xu, P.; Chang, L.; Wu, Q. Interleukin 4 Affects Epilepsy by Regulating Glial Cells: Potential and Possible Mechanism. *Frontiers in molecular neuroscience* **2020**, *13*, 554547, doi:10.3389/fnmol.2020.554547.
250. Zhang, S.; Kwan, P.; Baum, L. The potential role of CAMSAP1L1 in symptomatic epilepsy. *Neuroscience letters* **2013**, *556*, 146-151, doi:10.1016/j.neulet.2013.10.020.
251. Fu, C.Y.; Chen, S.J.; Cai, N.H.; Liu, Z.H.; Zhang, M.; Wang, P.C.; Zhao, J.N. Increased risk of post-stroke epilepsy in Chinese patients with a TRPM6 polymorphism. *Neurological research* **2019**, *41*, 378-383, doi:10.1080/01616412.2019.1568755.
252. Katano, M.; Numata, T.; Aguan, K.; Hara, Y.; Kiyonaka, S.; Yamamoto, S.; Miki, T.; Sawamura, S.; Suzuki, T.; Yamakawa, K.; et al. The juvenile myoclonic epilepsy-related protein EFHC1 interacts with the redox-sensitive TRPM2 channel linked to cell death. *Cell calcium* **2012**, *51*, 179-185, doi:10.1016/j.ceca.2011.12.011.
253. Tao, H.; Si, L.; Zhou, X.; Liu, Z.; Ma, Z.; Zhou, H.; Zhong, W.; Cui, L.; Zhang, S.; Li, Y.; et al. Role of glyoxalase I gene polymorphisms in late-onset epilepsy and drug-resistant epilepsy. *Journal of the neurological sciences* **2016**, *363*, 200-206, doi:10.1016/j.jns.2016.01.052.
254. Liang, Y.; Zhou, Z.; Wang, H.; Cheng, X.; Zhong, S.; Zhao, C. Association of apolipoprotein E genotypes with epilepsy risk: A systematic review and meta-analysis. *Epilepsy & behavior : E&B* **2019**, *98*, 27-35, doi:10.1016/j.yebeh.2019.06.015.
255. Adachi, M.; Abe, Y.; Aoki, Y.; Matsubara, Y. Epilepsy in RAS/MAPK syndrome: two cases of cardio-facio-cutaneous syndrome with epileptic encephalopathy and a literature review. *Seizure* **2012**, *21*, 55-60, doi:10.1016/j.seizure.2011.07.013.
256. Li, P.; Fu, X.; Smith, N.A.; Ziobro, J.; Curiel, J.; Tenga, M.J.; Martin, B.; Freedman, S.; Cea-Del Rio, C.A.; Oboti, L.; et al. Loss of CLOCK Results in Dysfunction of Brain Circuits Underlying Focal Epilepsy. *Neuron* **2017**, *96*, 387-401.e386, doi:10.1016/j.neuron.2017.09.044.
257. de Vries, E.E.; van den Munckhof, B.; Braun, K.P.; van Royen-Kerkhof, A.; de Jager, W.; Jansen, F.E. Inflammatory mediators in human epilepsy: A systematic review and meta-analysis. *Neuroscience and biobehavioral reviews* **2016**, *63*, 177-190, doi:10.1016/j.neubiorev.2016.02.007.
258. Bungenberg, J.; Surano, N.; Grote, A.; Surges, R.; Pernhorst, K.; Hofmann, A.; Schoch, S.; Helmstaedter, C.; Becker, A.J. Gene expression variance in hippocampal tissue of temporal lobe

epilepsy patients corresponds to differential memory performance. *Neurobiology of disease* **2016**, *86*, 121-130, doi:10.1016/j.nbd.2015.11.011.

259. Wong, J.K.L.; Gui, H.; Kwok, M.; Ng, P.W.; Lui, C.H.T.; Baum, L.; Sham, P.C.; Kwan, P.; Cherny, S.S. Rare variants and de novo variants in mesial temporal lobe epilepsy with hippocampal sclerosis. *Neurology. Genetics* **2018**, *4*(3):e245, doi:10.1212/nxg.0000000000000245.

260. Bragatti, J.A.; Bandeira, I.C.; de Carvalho, A.M.; Abujamra, A.L.; Leistner-Segal, S.; Bianchin, M.M. Tryptophan hydroxylase 2 (TPH2) gene polymorphisms and psychiatric comorbidities in temporal lobe epilepsy. *Epilepsy & behavior : E&B* **2014**, *32*, 59-63, doi:10.1016/j.yebeh.2014.01.007.

261. Chen, D.; Lu, Y.; Yu, W.; Luo, J.; Xiao, Z.; Xiao, F.; Wang, X. Clinical value of decreased superoxide dismutase 1 in patients with epilepsy. *Seizure* **2012**, *21*, 508-511, doi:10.1016/j.seizure.2012.05.003.

262. Ulbrich, L.; Cozzolino, M.; Marini, E.S.; Amori, I.; De Jaco, A.; Carrì, M.T.; Augusti-Tocco, G. Cystatin B and SOD1: protein–protein interaction and possible relation to neurodegeneration. *Cellular and molecular neurobiology* **2014**, *34*, 205-213, doi:10.1007/s10571-013-0004-y.

263. Pekmezci, M.; Villanueva-Meyer, J.E.; Goode, B.; Van Ziffle, J.; Onodera, C.; Grenert, J.P.; Bastian, B.C.; Chamyan, G.; Maher, O.M.; Khatib, Z.; et al. The genetic landscape of ganglioglioma. *Acta neuropathologica communications* **2018**, *6*, 47, doi:10.1186/s40478-018-0551-z.

264. Zhu, W.Y.; Jiang, P.; He, X.; Cao, L.J.; Zhang, L.H.; Dang, R.L.; Tang, M.M.; Xue, Y.; Li, H.D. Contribution of NRG1 Gene Polymorphisms in Temporal Lobe Epilepsy. *Journal of child neurology* **2016**, *31*, 271-276, doi:10.1177/0883073815589757.

265. Chioza, B.; Osei-Lah, A.; Wilkie, H.; Nashef, L.; McCormick, D.; Asherson, P.; Makoff, A.J. Suggestive evidence for association of two potassium channel genes with different idiopathic generalised epilepsy syndromes. *Epilepsy Res* **2002**, *52*, 107-116, doi:10.1016/s0920-1211(02)00195-x.

266. Zhang, Y.X.; Shen, C.H.; Guo, Y.; Zheng, Y.; Zhu, J.M.; Ding, Y.; Tang, Y.L.; Wang, S.; Ding, M.P. BRAF V600E mutation in epilepsy-associated glioneuronal tumors: Prevalence and correlation with clinical features in a Chinese population. *Seizure* **2017**, *45*, 102-106, doi:10.1016/j.seizure.2016.12.004.

267. Zhang, Y.; Xu, F.; Tan, Y.; Hu, J.; Wang, H. [Abnormal expression of PEX10 gene may be related to epilepsy associated with 1p36 copy number variations]. *Zhonghua yi xue yi chuan xue za zhi = Zhonghua yixue yichuanxue zazhi = Chinese journal of medical genetics* **2015**, *32*, 6-10, doi:10.3760/cma.j.issn.1003-9406.2015.01.002.

268. Carrozzo, R.; Verrigni, D.; Rasmussen, M.; de Coo, R.; Amartino, H.; Bianchi, M.; Buhas, D.; Mesli, S.; Naess, K.; Born, A.P.; et al. Succinate-CoA ligase deficiency due to mutations in SUCLA2 and SUCLG1: phenotype and genotype correlations in 71 patients. *Journal of inherited metabolic disease* **2016**, *39*, 243-252, doi:10.1007/s10545-015-9894-9.

269. Rozycka, A.; Dorszewska, J.; Steinborn, B.; Lianeri, M.; Winczewska-Wiktor, A.; Sniezawska, A.; Wisniewska, K.; Jagodzinski, P.P. Association study of the 2-bp deletion polymorphism in exon 6 of the CHRFAM7A gene with idiopathic generalized epilepsy. *DNA and cell biology* **2013**, *32*, 640-647, doi:10.1089/dna.2012.1880.

270. Yang, L.; You, C.; Qiu, S.; Yang, X.; Li, Y.; Liu, F.; Zhang, D.; Niu, Y.; Xu, L.; Xu, N.; et al. Novel and de novo point and large microdeletion mutation in PRRT2-related epilepsy. *Brain and behavior* **2020**, *10*, e01597, doi:10.1002/brb3.1597.

271. Tao, H.; Zhou, X.; Xie, Q.; Ma, Z.; Sun, F.; Cui, L.; Cai, Y.; Ma, G.; Fu, J.; Liu, Z.; et al. SRR intronic variation inhibits expression of its neighbouring SMG6 gene and protects against temporal lobe epilepsy. *Journal of cellular and molecular medicine* **2018**, *22*, 1883-1893, doi:10.1111/jcmm.13473.
272. Che, F.; Fu, Q.; Li, X.; Gao, N.; Qi, F.; Sun, Z.; Du, Y.; Li, M. Association of insulin receptor H1085H C>T, insulin receptor substrate 1 G972R and insulin receptor substrate 2 1057G/A polymorphisms with refractory temporal lobe epilepsy in Han Chinese. *Seizure* **2015**, *25*, 178-180, doi:10.1016/j.seizure.2014.09.014.
273. Borgatti, R.; Piccinelli, P.; Passoni, D.; Romeo, A.; Viri, M.; Musumeci, S.A.; Elia, M.; Cogliati, T.; Valseriati, D.; Grasso, R.; et al. Peripheral markers of the gamma-aminobutyric acid (GABA)ergic system in Angelman's syndrome. *Journal of child neurology* **2003**, *18*, 21-25, doi:10.1177/08830738030180010801.
274. Xi, B.; Chen, J.; Yang, L.; Wang, W.; Fu, M.; Wang, C. GABBR1 gene polymorphism(G1465A)isassociated with temporal lobe epilepsy. *Epilepsy Res* **2011**, *96*, 58-63, doi:10.1016/j.eplepsyres.2011.04.014.
275. Crèvecoeur, J.; Kaminski, R.M.; Rogister, B.; Foerch, P.; Vandenplas, C.; Neveux, M.; Mazzuferi, M.; Kroonen, J.; Poulet, C.; Martin, D.; et al. Expression pattern of synaptic vesicle protein 2 (SV2) isoforms in patients with temporal lobe epilepsy and hippocampal sclerosis. *Neuropathology and applied neurobiology* **2014**, *40*, 191-204, doi:10.1111/nan.12054.
276. Bayat, M.; Bayat, A. Neurological manifestations of neurofibromatosis: a review. *Neurological sciences : official journal of the Italian Neurological Society and of the Italian Society of Clinical Neurophysiology* **2020**, *41*, 2685-2690, doi:10.1007/s10072-020-04400-x.
277. Prabowo, A.S.; van Scheppingen, J.; Iyer, A.M.; Anink, J.J.; Spliet, W.G.; van Rijen, P.C.; Schouten-van Meeteren, A.Y.; Aronica, E. Differential expression and clinical significance of three inflammation-related microRNAs in gangliogliomas. *Journal of neuroinflammation* **2015**, *12*, 97, doi:10.1186/s12974-015-0315-7.
278. Spielmann, M.; Reichelt, G.; Hertzberg, C.; Trimborn, M.; Mundlos, S.; Horn, D.; Klopocki, E. Homozygous deletion of chromosome 15q13.3 including CHRNA7 causes severe mental retardation, seizures, muscular hypotonia, and the loss of KLF13 and TRPM1 potentially cause macrocytosis and congenital retinal dysfunction in siblings. *European journal of medical genetics* **2011**, *54*, e441-445, doi:10.1016/j.ejmg.2011.04.004.
279. Ercegovac, M.; Jovic, N.; Sokic, D.; Savic-Radojevic, A.; Coric, V.; Radic, T.; Nikolic, D.; Kecmanovic, M.; Matic, M.; Simic, T.; et al. GSTA1, GSTM1, GSTP1 and GSTT1 polymorphisms in progressive myoclonus epilepsy: A Serbian case-control study. *Seizure* **2015**, *32*, 30-36, doi:10.1016/j.seizure.2015.08.010.
280. Pathak, S.; Miller, J.; Morris, E.C.; Stewart, W.C.L.; Greenberg, D.A. DNA methylation of the BRD2 promoter is associated with juvenile myoclonic epilepsy in Caucasians. *Epilepsia* **2018**, *59*, 1011-1019, doi:10.1111/epi.14058.
281. Yilmaz, M.; Edgunlu, T.G.; Yilmaz, N.; Cetin, E.S.; Celik, S.K.; Emir, G.K.; Sözen, A. Genetic variants of synaptic vesicle and presynaptic plasma membrane proteins in idiopathic generalized epilepsy. *Journal of receptor and signal transduction research* **2014**, *34*, 38-43, doi:10.3109/10799893.2013.848893.
282. Baghel, R.; Grover, S.; Kaur, H.; Jajodia, A.; Parween, S.; Sinha, J.; Srivastava, A.; Srivastava, A.K.; Bala, K.; Chandna, P.; et al. Synergistic association of STX1A and VAMP2 with cryptogenic

epilepsy in North Indian population. *Brain and behavior* **2016**, *6*(7):e00490, doi:10.1002/brb3.490.
283. Stockhammer, F.; Misch, M.; Helms, H.J.; Lengler, U.; Prall, F.; von Deimling, A.; Hartmann, C. IDH1/2 mutations in WHO grade II astrocytomas associated with localization and seizure as the initial symptom. *Seizure* **2012**, *21*, 194-197, doi:10.1016/j.seizure.2011.12.007.
284. Hedenmalm, K.; Güzey, C.; Dahl, M.L.; Yue, Q.Y.; Spigset, O. Risk factors for extrapyramidal symptoms during treatment with selective serotonin reuptake inhibitors, including cytochrome P-450 enzyme, and serotonin and dopamine transporter and receptor polymorphisms. *Journal of clinical psychopharmacology* **2006**, *26*, 192-197, doi:10.1097/01.jcp.0000203200.96205.34.
285. Alcantara, J.A.; Vincentis, S.; Kerr, D.S.; Dos Santos, B.; Alessi, R.; van der Linden, H., Jr.; Chaim, T.; Serpa, M.H.; Busatto, G.F.; Gattaz, W.F.; et al. Association study of functional polymorphisms of dopaminergic pathway in epilepsy-related factors of temporal lobe epilepsy in Brazilian population. *European journal of neurology* **2018**, *25*, 895-901, doi:10.1111/ene.13631.
286. Genetic determinants of common epilepsies: a meta-analysis of genome-wide association studies. *The Lancet. Neurology* **2014**, *13*, 893-903, doi:10.1016/s1474-4422(14)70171-1.
287. Lal, D.; Ruppert, A.K.; Trucks, H.; Schulz, H.; de Kovel, C.G.; Kasteleijn-Nolst Trenité, D.; Sonsma, A.C.; Koeleman, B.P.; Lindhout, D.; Weber, Y.G.; et al. Burden analysis of rare microdeletions suggests a strong impact of neurodevelopmental genes in genetic generalised epilepsies. *PLoS genetics* **2015**, *11*(5):e1005226, doi:10.1371/journal.pgen.1005226.
288. Bonati, M.T.; Asselta, R.; Duga, S.; Ferini-Strambi, L.; Oldani, A.; Zucconi, M.; Malcovati, M.; Dalprà, L.; Tenchini, M.L. Refined mapping of CHRNA3/A5/B4 gene cluster and its implications in ADNFLE. *Neuroreport* **2000**, *11*, 2097-2101, doi:10.1097/00001756-200007140-00008.
289. Albuz, B.; Ozdemir, O.; Silan, F. The high frequency of chromosomal copy number variations and candidate genes in epilepsy patients. *Clinical neurology and neurosurgery* **2021**, *202*, 106487, doi:10.1016/j.clineuro.2021.106487.
290. Lukic, A.; Uphill, J.; Brown, C.A.; Beck, J.; Poulter, M.; Campbell, T.; Adamson, G.; Hummerich, H.; Whitfield, J.; Ponto, C.; et al. Rare structural genetic variation in human prion diseases. *Neurobiology of aging* **2015**, *36*, 2004.e2001-2008, doi:10.1016/j.neurobiolaging.2015.01.011.
291. Escalante-Santiago, D.; Feria-Romero, I.A.; Ribas-Aparicio, R.M.; Rayo-Mares, D.; Fagiolino, P.; Vázquez, M.; Escamilla-Núñez, C.; Grijalva-Otero, I.; López-García, M.A.; Orozco-Suárez, S. MDR-1 and MRP2 Gene Polymorphisms in Mexican Epileptic Pediatric Patients with Complex Partial Seizures. *Frontiers in neurology* **2014**, *5*, 184, doi:10.3389/fneur.2014.00184.
292. Layouni, S.; Buresi, C.; Thomas, P.; Malafosse, A.; Dogui, M. BRD2 and TAP-1 genes and juvenile myoclonic epilepsy. *Neurological sciences : official journal of the Italian Neurological Society and of the Italian Society of Clinical Neurophysiology* **2010**, *31*, 53-56, doi:10.1007/s10072-009-0190-z.
293. Emsley, H.C.; Appleton, R.E.; Whitmore, C.L.; Jury, F.; Lamb, J.A.; Martin, J.E.; Ollier, W.E.; de la Morandière, K.P.; Southern, K.W.; Allan, S.M. Variations in inflammation-related genes may be associated with childhood febrile seizure susceptibility. *Seizure* **2014**, *23*, 457-461, doi:10.1016/j.seizure.2014.03.006.
294. Zhou, L.; Zhang, M.; Long, H.; Long, L.; Xie, Y.; Liu, Z.; Kang, J.; Chen, Q.; Feng, L.; Xiao, B. Absence of association between major vault protein (MVP) gene polymorphisms and drug resistance in Chinese Han patients with partial epilepsy. *Journal of the neurological sciences* **2015**, *358*, 362-366, doi:10.1016/j.jns.2015.09.363.

295. Balan, S.; Radhab, S.K.; Sathyan, S.; Vijai, J.; Banerjee, M.; Radhakrishnan, K. Major vault protein (MVP) gene polymorphisms and drug resistance in mesial temporal lobe epilepsy with hippocampal sclerosis. *Gene* **2013**, *526*, 449-453, doi:10.1016/j.gene.2013.05.067.
296. Kong, A.N.; Fong, C.Y.; Ng, C.C.; Mohamed, A.R.; Khoo, T.B.; Ng, R.L.; Jalaludin, M.Y.; Nadarajaw, T. Association of common genetic variants with vitamin D status in Malaysian children with epilepsy. *Seizure* **2020**, *79*, 103-111, doi:10.1016/j.seizure.2020.05.009.
297. Nagai, Y.; Goldstein, L.H.; Critchley, H.D.; Fenwick, P.B. Influence of sympathetic autonomic arousal on cortical arousal: implications for a therapeutic behavioural intervention in epilepsy. *Epilepsy Res* **2004**, *58*, 185-193, doi:10.1016/j.eplepsyres.2004.02.004.
298. Ayoub, M.A.; Angelicheva, D.; Vile, D.; Chandler, D.; Morar, B.; Cavanaugh, J.A.; Visscher, P.M.; Jablensky, A.; Pfleger, K.D.; Kalaydjieva, L. Deleterious GRM1 mutations in schizophrenia. *PloS one* **2012**, *7*(3):e32849, doi:10.1371/journal.pone.0032849.
299. Sniezawska, A.; Dorszewska, J.; Rozycka, A.; Przedpelska-Ober, E.; Lianeri, M.; Jagodzinski, P.P.; Kozubski, W. MTHFR, MTR, and MTHFD1 gene polymorphisms compared to homocysteine and asymmetric dimethylarginine concentrations and their metabolites in epileptic patients treated with antiepileptic drugs. *Seizure* **2011**, *20*, 533-540, doi:10.1016/j.seizure.2011.04.001.
300. Schuch, J.B.; Muller, D.; Endres, R.G.; Bosa, C.A.; Longo, D.; Schuler-Faccini, L.; Ranzan, J.; Becker, M.M.; dos Santos Riesgo, R.; Roman, T. The role of β3 integrin gene variants in Autism Spectrum Disorders--diagnosis and symptomatology. *Gene* **2014**, *553*, 24-30, doi:10.1016/j.gene.2014.09.058.
301. Goldman, A.M.; Glasscock, E.; Yoo, J.; Chen, T.T.; Klassen, T.L.; Noebels, J.L. Arrhythmia in heart and brain: KCNQ1 mutations link epilepsy and sudden unexplained death. *Science translational medicine* **2009**, *1*, 2ra6, doi:10.1126/scitranslmed.3000289.
302. García Gozalo, M.; Bermejo Arnedo, I.; de Vera McMullan, P. KCNQ1 gene mutation and epilepsy in patient with long QT syndrome. *Medicina clinica* **2021**, *157*, 456-457, doi:10.1016/j.medcli.2020.09.008.
303. Li, X.; Liu, N.; Bai, R. Variant frequencies of KCNQ1, KCNH2, and SCN5A in a Chinese inherited arrhythmia cohort and other disease cohorts undergoing genetic testing. *Annals of human genetics* **2020**, *84*, 161-168, doi:10.1111/ahg.12359.
304. Mizuguchi, T.; Nakashima, M.; Kato, M.; Yamada, K.; Okanishi, T.; Ekhilevitch, N.; Mandel, H.; Eran, A.; Toyono, M.; Sawaishi, Y.; et al. PARS2 and NARS2 mutations in infantile-onset neurodegenerative disorder. *Journal of human genetics* **2017**, *62*, 525-529, doi:10.1038/jhg.2016.163.
305. Yin, X.; Tang, B.; Mao, X.; Peng, J.; Zeng, S.; Wang, Y.; Jiang, H.; Li, N. The genotypic and phenotypic spectrum of PARS2-related infantile-onset encephalopathy. *Journal of human genetics* **2018**, *63*, 971-980, doi:10.1038/s10038-018-0478-z.
306. Chatron, N.; Møller, R.S.; Champaigne, N.L.; Schneider, A.L.; Kuechler, A.; Labalme, A.; Simonet, T.; Baggett, L.; Bardel, C.; Kamsteeg, E.J.; et al. The epilepsy phenotypic spectrum associated with a recurrent CUX2 variant. *Annals of neurology* **2018**, *83*, 926-934, doi:10.1002/ana.25222.
307. Choi, J.; Kim, S.Y.; Kim, H.; Lim, B.C.; Hwang, H.; Chae, J.H.; Kim, K.J.; Oh, S.; Kim, E.Y.; Shin, J.S. Serum α-synuclein and IL-1β are increased and correlated with measures of disease severity in children with epilepsy: potential prognostic biomarkers? *BMC neurology* **2020**, *20*, 85, doi:10.1186/s12883-020-01662-y.
308. Poniatowski Ł, A.; Cudna, A.; Kurczych, K.; Bronisz, E.; Kurkowska-Jastrzębska, I. Kinetics of

serum brain-derived neurotrophic factor (BDNF) concentration levels in epileptic patients after generalized tonic-clonic seizures. *Epilepsy Res* **2021**, *173*, 106612, doi:10.1016/j.eplepsyres.2021.106612.
309. Doherty, C.; Hogue, O.; Floden, D.P.; Altemus, J.B.; Najm, I.M.; Eng, C.; Busch, R.M. BDNF and COMT, but not APOE, alleles are associated with psychiatric symptoms in refractory epilepsy. *Epilepsy & behavior : E&B* **2019**, *94*, 131-136, doi:10.1016/j.yebeh.2019.02.032.
310. Gkampeta, A.; Fidani, L.; Clarimón, J.; Kalinderi, K.; Katopodi, T.; Zafeiriou, D.; Pavlou, E. Association of brain-derived neurotrophic factor (BDNF) and elongator protein complex 4 (ELP4) polymorphisms with benign epilepsy with centrotemporal spikes in a Greek population. *Epilepsy Res* **2014**, *108*, 1734-1739, doi:10.1016/j.eplepsyres.2014.09.005.
311. Alcantara, J.A.; Vincentiis, S.; Santos, B.; Kerr, D.; de Paula, V.; Alessi, R.; Linden, H.; Chaim, T.; Serpa, M.; Busatto, G.; et al. BDNF Val66Met polymorphism is not related with temporal lobe epilepsy caused by hippocampal sclerosis in Brazilian population. *Seizure* **2018**, *60*, 159-162, doi:10.1016/j.seizure.2018.07.004.
312. Jiang, C.; Li, L.; Wu, M.; Hao, M.; Feng, J. Association of KCNJ10 variants and the susceptibility to clinical epilepsy. *Clinical neurology and neurosurgery* **2021**, *200*, 106340, doi:10.1016/j.clineuro.2020.106340.
313. Guo, Y.; Yan, K.P.; Qu, Q.; Qu, J.; Chen, Z.G.; Song, T.; Luo, X.Y.; Sun, Z.Y.; Bi, C.L.; Liu, J.F. Common variants of KCNJ10 are associated with susceptibility and anti-epileptic drug resistance in Chinese genetic generalized epilepsies. *PloS one* **2015**, *10*(4):e0124896, doi:10.1371/journal.pone.0124896.
314. Liubinas, S.V.; D'Abaco, G.M.; Moffat, B.M.; Gonzales, M.; Feleppa, F.; Nowell, C.J.; Gorelik, A.; Drummond, K.J.; O'Brien, T.J.; Kaye, A.H.; et al. IDH1 mutation is associated with seizures and protoplasmic subtype in patients with low-grade gliomas. *Epilepsia* **2014**, *55*, 1438-1443, doi:10.1111/epi.12662.
315. Kamaşak, T.; Dilber, B.; Yaman, S.; Durgut, B.D.; Kurt, T.; Çoban, E.; Arslan, E.A.; Şahin, S.; Karahan, S.C.; Cansu, A. HMGB-1, TLR4, IL-1R1, TNF-α, and IL-1β: novel epilepsy markers? *Epileptic disorders : international epilepsy journal with videotape* **2020**, *22*, 183-193, doi:10.1684/epd.2020.1155.
316. Glauser, T.A.; Holland, K.; O'Brien, V.P.; Keddache, M.; Martin, L.J.; Clark, P.O.; Cnaan, A.; Dlugos, D.; Hirtz, D.G.; Shinnar, S.; et al. Pharmacogenetics of antiepileptic drug efficacy in childhood absence epilepsy. *Annals of neurology* **2017**, *81*, 444-453, doi:10.1002/ana.24886.
317. Calhoun, J.D.; Hawkins, N.A.; Zachwieja, N.J.; Kearney, J.A. Cacna1g is a genetic modifier of epilepsy caused by mutation of voltage-gated sodium channel Scn2a. *Epilepsia* **2016**, *57*, e103-107, doi:10.1111/epi.13390.
318. Schijns, O.E.; Bisschop, J.; Rijkers, K.; Dings, J.; Vanherle, S.; Lindsey, P.; Smeets, H.J.; Hoogland, G. GAT-1 (rs2697153) and GAT-3 (rs2272400) polymorphisms are associated with febrile seizures and temporal lobe epilepsy. *Epileptic disorders : international epilepsy journal with videotape* **2020**, *22*, 176-182, doi:10.1684/epd.2020.1154.
319. Li, H.; Wang, X.; Zhou, Y.; Ni, G.; Su, Q.; Chen, Z.; Chen, Z.; Li, J.; Chen, X.; Hou, X.; et al. Association of LEPR and ANKK1 Gene Polymorphisms with Weight Gain in Epilepsy Patients Receiving Valproic Acid. *The international journal of neuropsychopharmacology* **2015**, *18*, pyv021, doi:10.1093/ijnp/pyv021.
320. Vincentiis, S.; Alcantara, J.; Rzezak, P.; Kerr, D.; Dos Santos, B.; Alessi, R.; van der Linden, H.;

Arruda, F.; Chaim-Avancini, T.; Serpa, M.; et al. Higher transcription alleles of the MAOA-uVNTR polymorphism are associated with higher seizure frequency in temporal lobe epilepsy. *Epilepsy Res* **2019**, *149*, 26-29, doi:10.1016/j.eplepsyres.2018.11.003.

321. Saitoh, M.; Kobayashi, K.; Ohmori, I.; Tanaka, Y.; Tanaka, K.; Inoue, T.; Horino, A.; Ohmura, K.; Kumakura, A.; Takei, Y.; et al. Cytokine-related and sodium channel polymorphism as candidate predisposing factors for childhood encephalopathy FIRES/AERRPS. *Journal of the neurological sciences* **2016**, *368*, 272-276, doi:10.1016/j.jns.2016.07.040.

322. Bartnik, M.; Szczepanik, E.; Derwińska, K.; Wiśniowiecka-Kowalnik, B.; Gambin, T.; Sykulski, M.; Ziemkiewicz, K.; Kędzior, M.; Gos, M.; Hoffman-Zacharska, D.; et al. Application of array comparative genomic hybridization in 102 patients with epilepsy and additional neurodevelopmental disorders. *American journal of medical genetics. Part B, Neuropsychiatric genetics : the official publication of the International Society of Psychiatric Genetics* **2012**, *159b*, 760-771, doi:10.1002/ajmg.b.32081.

323. Arend, J.; Kegler, A.; Caprara, A.L.F.; Gabbi, P.; Pascotini, E.T.; de Freitas, L.A.V.; Duarte, M.; Broetto, N.; Furian, A.F.; Oliveira, M.S.; et al. MnSOD Ala16Val polymorphism in cognitive dysfunction in patients with epilepsy: A relationship with oxidative and inflammatory markers. *Epilepsy & behavior : E&B* **2020**, *112*, 107346, doi:10.1016/j.yebeh.2020.107346.

324. Helbig, K.L.; Farwell Hagman, K.D.; Shinde, D.N.; Mroske, C.; Powis, Z.; Li, S.; Tang, S.; Helbig, I. Diagnostic exome sequencing provides a molecular diagnosis for a significant proportion of patients with epilepsy. *Genetics in medicine : official journal of the American College of Medical Genetics* **2016**, *18*, 898-905, doi:10.1038/gim.2015.186.

325. Tzschach, A.; Grasshoff, U.; Beck-Woedl, S.; Dufke, C.; Bauer, C.; Kehrer, M.; Evers, C.; Moog, U.; Oehl-Jaschkowitz, B.; Di Donato, N.; et al. Next-generation sequencing in X-linked intellectual disability. *European journal of human genetics : EJHG* **2015**, *23*, 1513-1518, doi:10.1038/ejhg.2015.5.

326. Steffens, M.; Leu, C.; Ruppert, A.K.; Zara, F.; Striano, P.; Robbiano, A.; Capovilla, G.; Tinuper, P.; Gambardella, A.; Bianchi, A.; et al. Genome-wide association analysis of genetic generalized epilepsies implicates susceptibility loci at 1q43, 2p16.1, 2q22.3 and 17q21.32. *Human molecular genetics* **2012**, *21*, 5359-5372, doi:10.1093/hmg/dds373.

327. Baker, K.; Astle, D.E.; Scerif, G.; Barnes, J.; Smith, J.; Moffat, G.; Gillard, J.; Baldeweg, T.; Raymond, F.L. Epilepsy, cognitive deficits and neuroanatomy in males with ZDHHC9 mutations. *Annals of clinical and translational neurology* **2015**, *2*, 559-569, doi:10.1002/acn3.196.

328. Baum, L.; Haerian, B.S.; Ng, H.K.; Wong, V.C.; Ng, P.W.; Lui, C.H.; Sin, N.C.; Zhang, C.; Tomlinson, B.; Wong, G.W.; et al. Case-control association study of polymorphisms in the voltage-gated sodium channel genes SCN1A, SCN2A, SCN3A, SCN1B, and SCN2B and epilepsy. *Human genetics* **2014**, *133*, 651-659, doi:10.1007/s00439-013-1405-1.

329. Møller, R.S.; Heron, S.E.; Larsen, L.H.; Lim, C.X.; Ricos, M.G.; Bayly, M.A.; van Kempen, M.J.; Klinkenberg, S.; Andrews, I.; Kelley, K.; et al. Mutations in KCNT1 cause a spectrum of focal epilepsies. *Epilepsia* **2015**, *56*, e114-120, doi:10.1111/epi.13071.

330. Jiang, Y.; Zhang, Y.; Zhang, P.; Sang, T.; Zhang, F.; Ji, T.; Huang, Q.; Xie, H.; Du, R.; Cai, B.; et al. NIPA2 located in 15q11.2 is mutated in patients with childhood absence epilepsy. *Human genetics* **2012**, *131*, 1217-1224, doi:10.1007/s00439-012-1149-3.

331. Zhan, A.; Xu, X.; Chen, L.; Wang, X.; Yanfeng, X.; Dan, W.; Zhan, Y.; Shi, Q. Decreased expression of Gab2 in patients with temporal lobe epilepsy and pilocarpine-induced rat model. *Synapse*

(New York, N.Y.) **2014**, *68*, 168-177, doi:10.1002/syn.21725.
332. Teocchi, M.A.; D'Souza-Li, L. Apoptosis through Death Receptors in Temporal Lobe Epilepsy-Associated Hippocampal Sclerosis. *Mediators of inflammation* **2016**, *2016*, 8290562, doi:10.1155/2016/8290562.
333. Myers, K.A.; Nasioulas, S.; Boys, A.; McMahon, J.M.; Slater, H.; Lockhart, P.; Sart, D.D.; Scheffer, I.E. ADGRV1 is implicated in myoclonic epilepsy. *Epilepsia* **2018**, *59*, 381-388, doi:10.1111/epi.13980.
334. Kegler, A.; Cardoso, A.S.; Caprara, A.L.F.; Pascotini, E.T.; Arend, J.; Gabbi, P.; Duarte, M.; da Cruz, I.B.M.; Furian, A.F.; Oliveira, M.S.; et al. Involvement of MnSOD Ala16Val polymorphism in epilepsy: A relationship with seizure type, inflammation, and metabolic syndrome. *Gene* **2019**, *711*, 143924, doi:10.1016/j.gene.2019.06.014.
335. Haerian, B.S.; Sha'ari, H.M.; Tan, H.J.; Fong, C.Y.; Wong, S.W.; Ong, L.C.; Raymond, A.A.; Tan, C.T.; Mohamed, Z. RORA gene rs12912233 and rs880626 polymorphisms and their interaction with SCN1A rs3812718 in the risk of epilepsy: a case-control study in Malaysia. *Genomics* **2015**, *105*, 229-236, doi:10.1016/j.ygeno.2015.02.001.
336. Kang, K.W.; Kim, W.; Cho, Y.W.; Lee, S.K.; Jung, K.Y.; Shin, W.; Kim, D.W.; Kim, W.J.; Lee, H.W.; Kim, W.; et al. Genetic characteristics of non-familial epilepsy. *PeerJ* **2019**, *7*, e8278, doi:10.7717/peerj.8278.
337. Sheilabi, M.A.; Takeshita, L.Y.; Sims, E.J.; Falciani, F.; Princivalle, A.P. The Sodium Channel B4-Subunits are Dysregulated in Temporal Lobe Epilepsy Drug-Resistant Patients. *International journal of molecular sciences* **2020**, *21*(8):2955, doi:10.3390/ijms21082955.
338. Parisi, P.; Oliva, A.; Coll Vidal, M.; Partemi, S.; Campuzano, O.; Iglesias, A.; Pisani, D.; Pascali, V.L.; Paolino, M.C.; Villa, M.P.; et al. Coexistence of epilepsy and Brugada syndrome in a family with SCN5A mutation. *Epilepsy Res* **2013**, *105*, 415-418, doi:10.1016/j.eplepsyres.2013.02.024.
339. Gorter, J.A.; Zurolo, E.; Iyer, A.; Fluiter, K.; van Vliet, E.A.; Baayen, J.C.; Aronica, E. Induction of sodium channel Na(x) (SCN7A) expression in rat and human hippocampus in temporal lobe epilepsy. *Epilepsia* **2010**, *51*, 1791-1800, doi:10.1111/j.1528-1167.2010.02678.x.
340. Johansen, A.; Rosti, R.O.; Musaev, D.; Sticca, E.; Harripaul, R.; Zaki, M.; Çağlayan, A.O.; Azam, M.; Sultan, T.; Froukh, T.; et al. Mutations in MBOAT7, Encoding Lysophosphatidylinositol Acyltransferase I, Lead to Intellectual Disability Accompanied by Epilepsy and Autistic Features. *American journal of human genetics* **2016**, *99*, 912-916, doi:10.1016/j.ajhg.2016.07.019.
341. Faheem, M.; Naseer, M.I.; Chaudhary, A.G.; Kumosani, T.A.; Rasool, M.; Algahtani, H.A.; Bibi, F.; Kamal, M.A.; Al-Qahtani, M.H. Array-comparative genomic hybridization analysis of a cohort of Saudi patients with epilepsy. *CNS & neurological disorders drug targets* **2015**, *14*, 468-475, doi:10.2174/1871527314666150429111737.
342. Jamali, S.; Salzmann, A.; Perroud, N.; Ponsole-Lenfant, M.; Cillario, J.; Roll, P.; Roeckel-Trevisiol, N.; Crespel, A.; Balzar, J.; Schlachter, K.; et al. Functional variant in complement C3 gene promoter and genetic susceptibility to temporal lobe epilepsy and febrile seizures. *PloS one* **2010**, *5*(9):e12740, doi:10.1371/journal.pone.0012740.
343. Ultra-Rare Genetic Variation in the Epilepsies: A Whole-Exome Sequencing Study of 17,606 Individuals. *American journal of human genetics* **2019**, *105*, 267-282, doi:10.1016/j.ajhg.2019.05.020.
344. Bando, S.Y.; Alegro, M.C.; Amaro, E., Jr.; Silva, A.V.; Castro, L.H.; Wen, H.T.; Lima Lde, A.; Brentani, H.; Moreira-Filho, C.A. Hippocampal CA3 transcriptome signature correlates with initial

precipitating injury in refractory mesial temporal lobe epilepsy. *PloS one* **2011**, *6*(10):e26268, doi:10.1371/journal.pone.0026268.

345. Duga, S.; Soldà, G.; Asselta, R.; Bonati, M.T.; Dalprà, L.; Malcovati, M.; Tenchini, M.L. Characterization of the genomic structure of the human neuronal nicotinic acetylcholine receptor CHRNA5/A3/B4 gene cluster and identification of novel intragenic polymorphisms. *Journal of human genetics* **2001**, *46*, 640-648, doi:10.1007/s100380170015.

346. Chen, T.T.; Klassen, T.L.; Goldman, A.M.; Marini, C.; Guerrini, R.; Noebels, J.L. Novel brain expression of ClC-1 chloride channels and enrichment of CLCN1 variants in epilepsy. *Neurology* **2013**, *80*, 1078-1085, doi:10.1212/WNL.0b013e31828868e7.

347. Allen, A.S.; Berkovic, S.F.; Cossette, P.; Delanty, N.; Dlugos, D.; Eichler, E.E.; Epstein, M.P.; Glauser, T.; Goldstein, D.B.; Han, Y.; et al. De novo mutations in epileptic encephalopathies. *Nature* **2013**, *501*, 217-221, doi:10.1038/nature12439.

348. Valmiki, R.R.; Venkatesalu, S.; Chacko, A.G.; Prabhu, K.; Thomas, M.M.; Mathew, V.; Yoganathan, S.; Muthusamy, K.; Chacko, G.; Vanjare, H.A.; et al. Phosphoproteomic analysis reveals Akt isoform-specific regulation of cytoskeleton proteins in human temporal lobe epilepsy with hippocampal sclerosis. *Neurochemistry international* **2020**, *134*, 104654, doi:10.1016/j.neuint.2019.104654.

349. Niturad, C.E.; Lev, D.; Kalscheuer, V.M.; Charzewska, A.; Schubert, J.; Lerman-Sagie, T.; Kroes, H.Y.; Oegema, R.; Traverso, M.; Specchio, N.; et al. Rare GABRA3 variants are associated with epileptic seizures, encephalopathy and dysmorphic features. *Brain : a journal of neurology* **2017**, *140*, 2879-2894, doi:10.1093/brain/awx236.

350. Li, Y.Q.; Xue, T.; Wang, L.; Xu, Z.C.; Xi, Z.Q.; Yuan, J.; Wang, X.F.; Chen, Y.M.; Zhang, M.; Yao, L. Up-regulation of epithelial membrane protein-1 in the temporal neocortex of patients with intractable epilepsy. *Neurochemical research* **2009**, *34*, 1594-1602, doi:10.1007/s11064-009-9948-1.

351. Bennett, J.; Gilkes, C.; Klassen, K.; Kerr, M.; Khan, A. Two Siblings With Valproate-Related Hyperammonemia and Novel Mutations in Glutamine Synthetase (GLUL) Treated With Carglumic Acid. *Child neurology open* **2020**, *7*, 2329048x20967880, doi:10.1177/2329048x20967880.

352. Inoue, K.; Takahashi, T.; Yamamoto, Y.; Suzuki, E.; Takahashi, Y.; Imai, K.; Inoue, Y.; Hirai, K.; Tsuji, D.; Itoh, K. Influence of glutamine synthetase gene polymorphisms on the development of hyperammonemia during valproic acid-based therapy. *Seizure* **2015**, *33*, 76-80, doi:10.1016/j.seizure.2015.10.015.

353. El-Hodhod, M.A.; Tomoum, H.Y.; Abd Al-Aziz, M.M.; Samaan, S.M. Serum Fas and Bcl-2 in patients with epilepsy. *Acta neurologica Scandinavica* **2006**, *113*, 315-321, doi:10.1111/j.1600-0404.2006.00592.x.

354. Manna, I.; Labate, A.; Mumoli, L.; Palamara, G.; Ferlazzo, E.; Aguglia, U.; Quattrone, A.; Gambardella, A. A functional genetic variation of the 5-HTR2A receptor affects age at onset in patients with temporal lobe epilepsy. *Annals of human genetics* **2012**, *76*, 277-282, doi:10.1111/j.1469-1809.2012.00713.x.

355. Jamali, S.; Bartolomei, F.; Robaglia-Schlupp, A.; Massacrier, A.; Peragut, J.C.; Régis, J.; Dufour, H.; Ravid, R.; Roll, P.; Pereira, S.; et al. Large-scale expression study of human mesial temporal lobe epilepsy: evidence for dysregulation of the neurotransmission and complement systems in the entorhinal cortex. *Brain : a journal of neurology* **2006**, *129*, 625-641,

doi:10.1093/brain/awl001.
356. Liu, Z.; Yin, X.; Liu, L.; Tao, H.; Zhou, H.; Ma, G.; Cui, L.; Li, Y.; Zhang, S.; Xu, Z.; et al. Association of KEAP1 and NFE2L2 polymorphisms with temporal lobe epilepsy and drug resistant epilepsy. *Gene* **2015**, *571*, 231-236, doi:10.1016/j.gene.2015.06.055.
357. Heuser, K.; Nagelhus, E.A.; Taubøll, E.; Indahl, U.; Berg, P.R.; Lien, S.; Nakken, S.; Gjerstad, L.; Ottersen, O.P. Variants of the genes encoding AQP4 and Kir4.1 are associated with subgroups of patients with temporal lobe epilepsy. *Epilepsy Res* **2010**, *88*, 55-64, doi:10.1016/j.eplepsyres.2009.09.023.
358. Zhu, H.; Zhang, M.; Fu, Y.; Long, H.; Xiao, W.; Feng, L.; Xiao, B.; Zhou, L. Effects of AQP4 and KCNJ10 Gene Polymorphisms on Drug Resistance and Seizure Susceptibility in Chinese Han Patients with Focal Epilepsy. *Neuropsychiatric disease and treatment* **2020**, *16*, 119-129, doi:10.2147/ndt.s231352.
359. Yalçin, O.; Baykan, B.; Ağan, K.; Yapici, Z.; Yalçin, D.; Dizdarer, G.; Türkdoğan, D.; Ozkara, C.; Unalp, A.; Uludüz, D.; et al. An association analysis at 2q36 reveals a new candidate susceptibility gene for juvenile absence epilepsy and/or absence seizures associated with generalized tonic-clonic seizures. *Epilepsia* **2011**, *52*, 975-983, doi:10.1111/j.1528-1167.2010.02970.x.
360. Chung, H.L.; Mao, X.; Wang, H.; Park, Y.J.; Marcogliese, P.C.; Rosenfeld, J.A.; Burrage, L.C.; Liu, P.; Murdock, D.R.; Yamamoto, S.; et al. De Novo Variants in CDK19 Are Associated with a Syndrome Involving Intellectual Disability and Epileptic Encephalopathy. *American journal of human genetics* **2020**, *106*, 717-725, doi:10.1016/j.ajhg.2020.04.001.
361. Belhedi, N.; Perroud, N.; Karege, F.; Vessaz, M.; Malafosse, A.; Salzmann, A. Increased CPA6 promoter methylation in focal epilepsy and in febrile seizures. *Epilepsy Res* **2014**, *108*, 144-148, doi:10.1016/j.eplepsyres.2013.10.007.
362. Murakami, Y.; Nguyen, T.T.M.; Baratang, N.; Raju, P.K.; Knaus, A.; Ellard, S.; Jones, G.; Lace, B.; Rousseau, J.; Ajeawung, N.F.; et al. Mutations in PIGB Cause an Inherited GPI Biosynthesis Defect with an Axonal Neuropathy and Metabolic Abnormality in Severe Cases. *American journal of human genetics* **2019**, *105*, 384-394, doi:10.1016/j.ajhg.2019.05.019.
363. He, J.; Zhou, W.; Shi, J.; Zhang, B.; Wang, H. A Chinese patient with epilepsy and WWOX compound heterozygous mutations. *Epileptic disorders : international epilepsy journal with videotape* **2020**, *22*, 120-124, doi:10.1684/epd.2020.1131.
364. Koga, Y.; Tsuchimoto, D.; Hayashi, Y.; Abolhassani, N.; Yoneshima, Y.; Sakumi, K.; Nakanishi, H.; Toyokuni, S.; Nakabeppu, Y. Neural stem cell-specific ITPA deficiency causes neural depolarization and epilepsy. *JCI insight* **2020**, *5*, doi:10.1172/jci.insight.140229.
365. Luan, G.; Gao, Q.; Zhai, F.; Chen, Y.; Li, T. Upregulation of HMGB1, toll-like receptor and RAGE in human Rasmussen's encephalitis. *Epilepsy Res* **2016**, *123*, 36-49, doi:10.1016/j.eplepsyres.2016.03.005.
366. van Karnebeek, C.D.M.; Ramos, R.J.; Wen, X.Y.; Tarailo-Graovac, M.; Gleeson, J.G.; Skrypnyk, C.; Brand-Arzamendi, K.; Karbassi, F.; Issa, M.Y.; van der Lee, R.; et al. Bi-allelic GOT2 Mutations Cause a Treatable Malate-Aspartate Shuttle-Related Encephalopathy. *American journal of human genetics* **2019**, *105*, 534-548, doi:10.1016/j.ajhg.2019.07.015.
367. Gartner, V.; Markello, T.C.; Macnamara, E.; De Biase, A.; Thurm, A.; Joseph, L.; Beggs, A.; Schmahmann, J.D.; Berry, G.T.; Anselm, I.; et al. Novel variants in SPTAN1 without epilepsy: An expansion of the phenotype. *American journal of medical genetics. Part A* **2018**, *176*, 2768-

2776, doi:10.1002/ajmg.a.40628.
368. Tohyama, J.; Nakashima, M.; Nabatame, S.; Gaik-Siew, C.; Miyata, R.; Rener-Primec, Z.; Kato, M.; Matsumoto, N.; Saitsu, H. SPTAN1 encephalopathy: distinct phenotypes and genotypes. *Journal of human genetics* **2015**, *60*, 167-173, doi:10.1038/jhg.2015.5.
369. Meguid, N.A.; Samir, H.; Bjørklund, G.; Anwar, M.; Hashish, A.; Koura, F.; Chirumbolo, S.; Hashem, S.; El-Bana, M.A.; Hashem, H.S. Altered S100 Calcium-Binding Protein B and Matrix Metallopeptidase 9 as Biomarkers of Mesial Temporal Lobe Epilepsy with Hippocampus Sclerosis. *Journal of molecular neuroscience : MN* **2018**, *66*, 482-491, doi:10.1007/s12031-018-1164-5.
370. Wu, C.; Zhang, G.; Chen, L.; Kim, S.; Yu, J.; Hu, G.; Chen, J.; Huang, Y.; Zheng, G.; Huang, S. The Role of NLRP3 and IL-1β in Refractory Epilepsy Brain Injury. *Frontiers in neurology* **2019**, *10*, 1418, doi:10.3389/fneur.2019.01418.
371. Li, Y.; Wu, L.; Yu, M.; Yang, F.; Wu, B.; Lu, S.; Tu, M.; Xu, H. HIF-1α is Critical for the Activation of Notch Signaling in Neurogenesis During Acute Epilepsy. *Neuroscience* **2018**, *394*, 206-219, doi:10.1016/j.neuroscience.2018.10.037.
372. Mahmoud, A.A.; Abdelmagid, T.; AlGhofely, M.; Hamed, A.M.; Al Sharif, R. Epilepsy in patients with insulin-dependent diabetes and relation to glutamic acid decarboxylase 65. *Neurosciences (Riyadh, Saudi Arabia)* **2020**, *25*, 200-204, doi:10.17712/nsj.2020.3.20190057.
373. Fan, X.; Chen, Y.; Li, W.; Xia, H.; Liu, B.; Guo, H.; Yang, Y.; Xu, C.; Xie, S.; Xu, X. Genetic Polymorphism of ADORA2A Is Associated With the Risk of Epilepsy and Predisposition to Neurologic Comorbidity in Chinese Southern Children. *Front Neurosci* **2020**, *14*, 590605, doi:10.3389/fnins.2020.590605.
374. Suchkova, I.O.; Borisova, E.V.; Patkin, E.L. Length Polymorphism and Methylation Status of UPS29 Minisatellite of the ACAP3 Gene as Molecular Biomarker of Epilepsy. Sex Differences in Seizure Types and Symptoms. *International journal of molecular sciences* **2020**, *21*, doi:10.3390/ijms21239206.
375. Feyissa, A.M.; Carrano, A.; Wang, X.; Allen, M.; Ertekin-Taner, N.; Dickson, D.W.; Jentoft, M.E.; Rosenfeld, S.S.; Tatum, W.O.; Ritaccio, A.L.; et al. Analysis of intraoperative human brain tissue transcriptome reveals putative risk genes and altered molecular pathways in glioma-related seizures. *Epilepsy Res* **2021**, *173*, 106618, doi:10.1016/j.eplepsyres.2021.106618.
376. Zheng, Z.; Yan, Y.; Guo, Q.; Wang, L.; Han, X.; Liu, S. Genetic Interaction of H19 and TGFBR1 Polymorphisms with Risk of Epilepsy in a Chinese Population. *Pharmacogenomics and personalized medicine* **2021**, *14*, 77-86, doi:10.2147/pgpm.s279664.
377. Cristina de Brito Toscano, E.; Leandro Marciano Vieira, É.; Boni Rocha Dias, B.; Vidigal Caliari, M.; Paula Gonçalves, A.; Varela Giannetti, A.; Maurício Siqueira, J.; Kimie Suemoto, C.; Elaine Paraizo Leite, R.; Nitrini, R.; et al. NLRP3 and NLRP1 inflammasomes are up-regulated in patients with mesial temporal lobe epilepsy and may contribute to overexpression of caspase-1 and IL-β in sclerotic hippocampi. *Brain research* **2021**, *1752*, 147230, doi:10.1016/j.brainres.2020.147230.
378. Hu, X.; Tang, J.; Hua, Y.; Wang, Y.; Huang, J. Evaluation of candidate genes in a Chinese cohort of atypical Rolandic epilepsy. *Epileptic disorders : international epilepsy journal with videotape* **2021**, *23*, 623-632, doi:10.1684/epd.2021.1308.
379. Sazci, G.; Sazci, B.; Sazci, A.; Idrisoglu, H.A. Association of Nicotinamide-N-Methyltransferase Gene rs694539 Variant with Epilepsy. *Molecular neurobiology* **2016**, *53*, 4197-4200,

doi:10.1007/s12035-015-9364-2.
380. Stogmann, E.; Reinthaler, E.; Eltawil, S.; El Etribi, M.A.; Hemeda, M.; El Nahhas, N.; Gaber, A.M.; Fouad, A.; Edris, S.; Benet-Pages, A.; et al. Autosomal recessive cortical myoclonic tremor and epilepsy: association with a mutation in the potassium channel associated gene CNTN2. *Brain : a journal of neurology* **2013**, *136*, 1155-1160, doi:10.1093/brain/awt068.
381. Sugimoto, Y.; Morita, R.; Amano, K.; Shah, P.U.; Pascual-Castroviejo, I.; Khan, S.; Delgado-Escueta, A.V.; Yamakawa, K. T-STAR gene: fine mapping in the candidate region for childhood absence epilepsy on 8q24 and mutational analysis in patients. *Epilepsy Res* **2001**, *46*, 139-144, doi:10.1016/s0920-1211(01)00274-1.
382. Reinthaler, E.M.; Lal, D.; Jurkowski, W.; Feucht, M.; Steinböck, H.; Gruber-Sedlmayr, U.; Ronen, G.M.; Geldner, J.; Haberlandt, E.; Neophytou, B.; et al. Analysis of ELP4, SRPX2, and interacting genes in typical and atypical rolandic epilepsy. *Epilepsia* **2014**, *55*, e89-93, doi:10.1111/epi.12712.
383. Tsai, M.H.; Muir, A.M.; Wang, W.J.; Kang, Y.N.; Yang, K.C.; Chao, N.H.; Wu, M.F.; Chang, Y.C.; Porter, B.E.; Jansen, L.A.; et al. Pathogenic Variants in CEP85L Cause Sporadic and Familial Posterior Predominant Lissencephaly. *Neuron* **2020**, *106*, 237-245.e238, doi:10.1016/j.neuron.2020.01.027.
384. Alcántara-Ortigoza, M.A.; García-de Teresa, B.; González-Del Angel, A.; Berumen, J.; Guardado-Estrada, M.; Fernández-Hernández, L.; Navarrete-Martínez, J.I.; Maza-Morales, M.; Rius-Domínguez, R. Wide allelic heterogeneity with predominance of large IDS gene complex rearrangements in a sample of Mexican patients with Hunter syndrome. *Clinical genetics* **2016**, *89*, 574-583, doi:10.1111/cge.12738.
385. Chen, L.; Tian, Q.; Zhang, M.; Chen, D.; Gao, X.; Yang, H.; Li, H.; Li, C.; Wen, J.; Li, Y.; et al. CPS1 T1405N polymorphism, HDL cholesterol, homocysteine and renal function are risk factors of VPA induced hyperammonemia among epilepsy patients. *Epilepsy Res* **2019**, *154*, 139-143, doi:10.1016/j.eplepsyres.2019.05.010.
386. Grover, S.; Kukreti, R. A systematic review and meta-analysis of the role of ABCC2 variants on drug response in patients with epilepsy. *Epilepsia* **2013**, *54*, 936-945, doi:10.1111/epi.12132.
387. Smit, D.J.A.; Wright, M.J.; Meyers, J.L.; Martin, N.G.; Ho, Y.Y.W.; Malone, S.M.; Zhang, J.; Burwell, S.J.; Chorlian, D.B.; de Geus, E.J.C.; et al. Genome-wide association analysis links multiple psychiatric liability genes to oscillatory brain activity. *Human brain mapping* **2018**, *39*, 4183-4195, doi:10.1002/hbm.24238.
388. Wang, R.; Lei, T.; Fu, F.; Li, R.; Jing, X.; Yang, X.; Liu, J.; Li, D.; Liao, C. Application of chromosome microarray analysis in patients with unexplained developmental delay/intellectual disability in South China. *Pediatrics and neonatology* **2019**, *60*, 35-42, doi:10.1016/j.pedneo.2018.03.006.
389. Huang, M.C.; Okada, M.; Nakatsu, F.; Oguni, H.; Ito, M.; Morita, K.; Nagafuji, H.; Hirose, S.; Sakaki, Y.; Kaneko, S.; et al. Mutation screening of AP3M2 in Japanese epilepsy patients. *Brain & development* **2007**, *29*, 462-467, doi:10.1016/j.braindev.2006.12.004.
390. Takahashi, Y.; Mogami, Y.; Mine, J.; Imai, K.; Koide, Y.; Matsuda, K.; Akasaka, N.; Konishi, T.; Imamura, A.; Inoue, Y. Genetic variations of immunoregulatory genes associated with Rasmussen syndrome. *Epilepsy Res* **2013**, *107*, 238-243, doi:10.1016/j.eplepsyres.2013.09.004.
391. Baker, K.; Scerif, G.; Astle, D.E.; Fletcher, P.C.; Raymond, F.L. Psychopathology and cognitive performance in individuals with membrane-associated guanylate kinase mutations: a functional network phenotyping study. *Journal of neurodevelopmental disorders* **2015**, *7*, 8,

doi:10.1186/s11689-015-9105-x.
392. Lenzen, K.P.; Heils, A.; Lorenz, S.; Hempelmann, A.; Sander, T. Association analysis of malic enzyme 2 gene polymorphisms with idiopathic generalized epilepsy. *Epilepsia* **2005**, *46*, 1637-1641, doi:10.1111/j.1528-1167.2005.00270.x.
393. Chouchi, M.; Klaa, H.; Ben-Youssef Turki, I.; Hila, L. ABCB1 Polymorphisms and Drug-Resistant Epilepsy in a Tunisian Population. *Disease markers* **2019**, *2019*, 1343650, doi:10.1155/2019/1343650.
394. Silva-Alves, M.S.; Secolin, R.; Carvalho, B.S.; Yasuda, C.L.; Bilevicius, E.; Alvim, M.K.; Santos, R.O.; Maurer-Morelli, C.V.; Cendes, F.; Lopes-Cendes, I. A Prediction Algorithm for Drug Response in Patients with Mesial Temporal Lobe Epilepsy Based on Clinical and Genetic Information. *PloS one* **2017**, *12*(1):e0169214, doi:10.1371/journal.pone.0169214.
395. Yoshimura, H.; Hashimoto, T.; Murata, T.; Fukushima, K.; Sugaya, A.; Nishio, S.Y.; Usami, S. Novel ABHD12 mutations in PHARC patients: the differential diagnosis of deaf-blindness. *The Annals of otology, rhinology, and laryngology* **2015**, *124 Suppl 1*, 77s-83s, doi:10.1177/0003489415574513.
396. Zhou, Y.; Wang, X.; Li, H.; Zhang, J.; Chen, Z.; Xie, W.; Zhang, J.; Li, J.; Zhou, L.; Huang, M. Polymorphisms of ABCG2, ABCB1 and HNF4α are associated with Lamotrigine trough concentrations in epilepsy patients. *Drug metabolism and pharmacokinetics* **2015**, *30*, 282-287, doi:10.1016/j.dmpk.2015.05.002.
397. Zhang, N.; Ouyang, T.H.; Zhou, Q.; Kang, H.C.; Zhu, S.Q. Prodynorphin gene promoter polymorphism and temporal lobe epilepsy: A meta-analysis. *Journal of Huazhong University of Science and Technology. Medical sciences = Hua zhong ke ji da xue xue bao. Yi xue Ying De wen ban = Huazhong keji daxue xuebao. Yixue Yingdewen ban* **2015**, *35*, 635-639, doi:10.1007/s11596-015-1482-6.
398. Jiang, Y.H.; Yuen, R.K.; Jin, X.; Wang, M.; Chen, N.; Wu, X.; Ju, J.; Mei, J.; Shi, Y.; He, M.; et al. Detection of clinically relevant genetic variants in autism spectrum disorder by whole-genome sequencing. *American journal of human genetics* **2013**, *93*, 249-263, doi:10.1016/j.ajhg.2013.06.012.
399. Levinson, D.F.; Duan, J.; Oh, S.; Wang, K.; Sanders, A.R.; Shi, J.; Zhang, N.; Mowry, B.J.; Olincy, A.; Amin, F.; et al. Copy number variants in schizophrenia: confirmation of five previous findings and new evidence for 3q29 microdeletions and VIPR2 duplications. *The American journal of psychiatry* **2011**, *168*, 302-316, doi:10.1176/appi.ajp.2010.10060876.
400. He, X.; Li, Y.; Liu, Z.; Yue, X.; Zhao, P.; Hu, J.; Wu, G.; Mao, B.; Sun, D.; Zhang, H.; et al. The association between CCL2 polymorphisms and drug-resistant epilepsy in Chinese children. *Epileptic disorders : international epilepsy journal with videotape* **2013**, *15*, 272-277, doi:10.1684/epd.2013.0603.
401. Bagnall, R.D.; Crompton, D.E.; Cutmore, C.; Regan, B.M.; Berkovic, S.F.; Scheffer, I.E.; Semsarian, C. Genetic analysis of PHOX2B in sudden unexpected death in epilepsy cases. *Neurology* **2014**, *83*, 1018-1021, doi:10.1212/wnl.0000000000000781.
402. Shangguan, Y.; Xu, X.; Ganbat, B.; Li, Y.; Wang, W.; Yang, Y.; Lu, X.; Du, C.; Tian, X.; Wang, X. CNTNAP4 Impacts Epilepsy Through GABAA Receptors Regulation: Evidence From Temporal Lobe Epilepsy Patients and Mouse Models. *Cerebral cortex (New York, N.Y. : 1991)* **2018**, *28*, 3491-3504, doi:10.1093/cercor/bhx215.
403. Riazanski, V.; Deriy, L.V.; Shevchenko, P.D.; Le, B.; Gomez, E.A.; Nelson, D.J. Presynaptic CLC-3

determines quantal size of inhibitory transmission in the hippocampus. *Nature neuroscience* **2011**, *14*, 487-494, doi:10.1038/nn.2775.

404. Li, K.; Kong, D.S.; Zhang, J.; Wang, X.S.; Ye, X.; Zhao, Y.L. Association between ELP4 rs986527 polymorphism and the occurrence and development of intracranial arachnoid cyst. *Brain and behavior* **2019**, *9*, e01480, doi:10.1002/brb3.1480.

405. Saruwatari, J.; Deguchi, M.; Yoshimori, Y.; Noai, M.; Yoshida, S.; Ogusu, N.; Oniki, K.; Yoshida, S.; Yasui-Furukori, N.; Kaneko, S.; et al. Superoxide dismutase 2 Val16Ala polymorphism is a risk factor for the valproic acid-related elevation of serum aminotransferases. *Epilepsy Res* **2012**, *99*, 183-186, doi:10.1016/j.eplepsyres.2011.10.033.

406. Kwan, P.; Wong, V.; Ng, P.W.; Lui, C.H.; Sin, N.C.; Wong, K.S.; Baum, L. Gene-wide tagging study of the association between ABCC2, ABCC5 and ABCG2 genetic polymorphisms and multidrug resistance in epilepsy. *Pharmacogenomics* **2011**, *12*, 319-325, doi:10.2217/pgs.10.183.

407. Nellist, M.; Schot, R.; Hoogeveen-Westerveld, M.; Neuteboom, R.F.; van der Louw, E.J.; Lequin, M.H.; Bindels-de Heus, K.; Sibbles, B.J.; de Coo, R.; Brooks, A.; et al. Germline activating AKT3 mutation associated with megalencephaly, polymicrogyria, epilepsy and hypoglycemia. *Molecular genetics and metabolism* **2015**, *114*, 467-473, doi:10.1016/j.ymgme.2014.11.018.

408. Haberlandt, E.; Rauchenzauner, M.; Morass, M.; Wondrak, P.; Scholl-Bürgi, S.; Rostásy, K.; Karall, D. Matrix-metalloproteinases and proinflammatory cytokines in children with febrile convulsions and epilepsy--cause or consequence? *Epilepsy Res* **2013**, *105*, 225-228, doi:10.1016/j.eplepsyres.2012.12.003.

409. Diociaiuti, A.; Martinelli, D.; Nicita, F.; Cesario, C.; Pisaneschi, E.; Macchiaiolo, M.; Rossi, S.; Condorelli, A.G.; Zambruno, G.; El Hachem, M. Two Italian Patients with ELOVL4-Related Neuro-Ichthyosis: Expanding the Genotypic and Phenotypic Spectrum and Ultrastructural Characterization. *Genes* **2021**, *12*(3):343 doi:10.3390/genes12030343.

410. Xian, W.; Tao, H.; Zhao, J.; Fu, J.; Zhong, W.; Chen, Y.; Zhou, H.; Li, K.; Pan, S. Association Between Clusterin Gene Polymorphisms and Epilepsy in a Han Chinese Population. *Genetic testing and molecular biomarkers* **2017**, *21*, 692-697, doi:10.1089/gtmb.2017.0032.

411. Zhu, X.; Yun, W.; Sun, X.; Qiu, F.; Zhao, L.; Guo, Y. Effects of major transporter and metabolizing enzyme gene polymorphisms on carbamazepine metabolism in Chinese patients with epilepsy. *Pharmacogenomics* **2014**, *15*, 1867-1879, doi:10.2217/pgs.14.142.

412. Lien, E.; Andersen, G.; Bao, Y.; Gordish-Dressman, H.; Skranes, J.S.; Blackman, J.A.; Vik, T. Genes determining the severity of cerebral palsy: the role of single nucleotide polymorphisms on the amount and structure of apolipoprotein E. *Acta paediatrica (Oslo, Norway : 1992)* **2015**, *104*, 701-706, doi:10.1111/apa.12983.

413. Kim, J.E.; Ko, A.R.; Hyun, H.W.; Min, S.J.; Kang, T.C. P2RX7-MAPK1/2-SP1 axis inhibits MTOR independent HSPB1-mediated astroglial autophagy. *Cell death & disease* **2018**, *9*, 546, doi:10.1038/s41419-018-0586-x.

414. Fricke-Galindo, I.; Jung-Cook, H.; A, L.L.; López-López, M. Pharmacogenetics of adverse reactions to antiepileptic drugs. *Neurologia (Engl Ed)* **2018**, *33*, 165-176, doi:10.1016/j.nrl.2015.03.005.

415. McCormack, M.; Gui, H.; Ingason, A.; Speed, D.; Wright, G.E.B.; Zhang, E.J.; Secolin, R.; Yasuda, C.; Kwok, M.; Wolking, S.; et al. Genetic variation in CFH predicts phenytoin-induced maculopapular exanthema in European-descent patients. *Neurology* **2018**, *90*, e332-e341, doi:10.1212/wnl.0000000000004853.

416. Hata, Y.; Hirono, K.; Yamaguchi, Y.; Ichida, F.; Oku, Y.; Nishida, N. Minimal inflammatory foci of unknown etiology may be a tentative sign of early stage inherited cardiomyopathy. *Modern pathology : an official journal of the United States and Canadian Academy of Pathology, Inc* **2019**, *32*, 1281-1290, doi:10.1038/s41379-019-0274-0.
417. Manna, I.; Labate, A.; Mumoli, L.; Ferlazzo, E.; Aguglia, U.; Quattrone, A.; Gambardella, A. Failure to confirm association of a polymorphism in KCNMB4 gene with mesial temporal lobe epilepsy. *Epilepsy Res* **2013**, *106*, 284-287, doi:10.1016/j.eplepsyres.2013.03.014.
418. Liu, Z.; Fang, F.; Ding, C.; Wu, H.; Lyu, J.; Wu, Y. [SUCLA2-related encephalomyopathic mitochondrial DNA depletion syndrome: a case report and review of literature]. *Zhonghua er ke za zhi = Chinese journal of pediatrics* **2014**, *52*, 817-821.
419. Cukier, H.N.; Lee, J.M.; Ma, D.; Young, J.I.; Mayo, V.; Butler, B.L.; Ramsook, S.S.; Rantus, J.A.; Abrams, A.J.; Whitehead, P.L.; et al. The expanding role of MBD genes in autism: identification of a MECP2 duplication and novel alterations in MBD5, MBD6, and SETDB1. *Autism research : official journal of the International Society for Autism Research* **2012**, *5*, 385-397, doi:10.1002/aur.1251.
420. Manna, I.; Labate, A.; Mumoli, L.; Ferlazzo, E.; Aguglia, U.; Quattrone, A.; Gambardella, A. No evidence for a role of the coding variant of the Toll-like receptor 4 gene in temporal lobe epilepsy. *Seizure* **2013**, *22*, 791-793, doi:10.1016/j.seizure.2013.05.012.
421. Boussadia, B.; Ghosh, C.; Plaud, C.; Pascussi, J.M.; de Bock, F.; Rousset, M.C.; Janigro, D.; Marchi, N. Effect of status epilepticus and antiepileptic drugs on CYP2E1 brain expression. *Neuroscience* **2014**, *281*, 124-134, doi:10.1016/j.neuroscience.2014.09.055.
422. Yoon, H.Y.; Ahn, M.H.; Yee, J.; Lee, N.; Han, J.M.; Gwak, H.S. Influence of CYP2C9 and CYP2A6 on plasma concentrations of valproic acid: a meta-analysis. *European journal of clinical pharmacology* **2020**, *76*, 1053-1058, doi:10.1007/s00228-020-02872-6.
423. Mesdjian, E.; Sérée, E.; Charvet, B.; Mirrione, A.; Bourgarel-Rey, V.; Desobry, A.; Barra, Y. Metabolism of carbamazepine by CYP3A6: a model for in vitro drug interactions studies. *Life sciences* **1999**, *64*, 827-835, doi:10.1016/s0024-3205(99)00004-1.
424. Hung, C.C.; Jen Tai, J.; Kao, P.J.; Lin, M.S.; Liou, H.H. Association of polymorphisms in NR1I2 and ABCB1 genes with epilepsy treatment responses. *Pharmacogenomics* **2007**, *8*, 1151-1158, doi:10.2217/14622416.8.9.1151.
425. Amanat, S.; Gallego-Martinez, A.; Sollini, J.; Perez-Carpena, P.; Espinosa-Sanchez, J.M.; Aran, I.; Soto-Varela, A.; Batuecas-Caletrio, A.; Canlon, B.; May, P.; et al. Burden of rare variants in synaptic genes in patients with severe tinnitus: An exome based extreme phenotype study. *EBioMedicine* **2021**, *66*, 103309, doi:10.1016/j.ebiom.2021.103309.
426. Aksenova, M.G.; Burd, S.G.; Kachalin, E.; Avakian, G.N.; Badalian, O.L.; Savenkov, A.A.; Tertyshnik, O.; Dorofeeva, M.; Belousova, E.D.; Gusev, E.I. [An association between the FABP2 gene polymorphism and efficacy of valproates]. *Zhurnal nevrologii i psikhiatrii imeni S.S. Korsakova* **2007**, *107*, 42-45.
427. Rahman, M.T.; Ghosh, C.; Hossain, M.; Linfield, D.; Rezaee, F.; Janigro, D.; Marchi, N.; van Boxel-Dezaire, A.H.H. IFN-γ, IL-17A, or zonulin rapidly increase the permeability of the blood-brain and small intestinal epithelial barriers: Relevance for neuro-inflammatory diseases. *Biochemical and biophysical research communications* **2018**, *507*, 274-279, doi:10.1016/j.bbrc.2018.11.021.
428. Stein, J.L.; Medland, S.E.; Vasquez, A.A.; Hibar, D.P.; Senstad, R.E.; Winkler, A.M.; Toro, R.; Appel,

K.; Bartecek, R.; Bergmann, Ø.; et al. Identification of common variants associated with human hippocampal and intracranial volumes. *Nature genetics* **2012**, *44*, 552-561, doi:10.1038/ng.2250.

429. Lv, N.; Qu, J.; Long, H.; Zhou, L.; Cao, Y.; Long, L.; Liu, Z.; Xiao, B. Association study between polymorphisms in the CACNA1A, CACNA1C, and CACNA1H genes and drug-resistant epilepsy in the Chinese Han population. *Seizure* **2015**, *30*, 64-69, doi:10.1016/j.seizure.2015.05.013.

430. Pinggera, A.; Mackenroth, L.; Rump, A.; Schallner, J.; Beleggia, F.; Wollnik, B.; Striessnig, J. New gain-of-function mutation shows CACNA1D as recurrently mutated gene in autism spectrum disorders and epilepsy. *Human molecular genetics* **2017**, *26*, 2923-2932, doi:10.1093/hmg/ddx175.

431. Speed, D.; Hoggart, C.; Petrovski, S.; Tachmazidou, I.; Coffey, A.; Jorgensen, A.; Eleftherohorinou, H.; De Iorio, M.; Todaro, M.; De, T.; et al. A genome-wide association study and biological pathway analysis of epilepsy prognosis in a prospective cohort of newly treated epilepsy. *Human molecular genetics* **2014**, *23*, 247-258, doi:10.1093/hmg/ddt403.

432. Haerian, B.S.; Lim, K.S.; Tan, H.J.; Wong, C.P.; Wong, S.W.; Tan, C.T.; Raymond, A.A.; Mohamed, Z. Lack of association between synapsin II (SYN2) gene polymorphism and susceptibility epilepsy: a case-control study and meta-analysis. *Synapse (New York, N.Y.)* **2011**, *65*, 1073-1079, doi:10.1002/syn.20939.

433. Cantarín-Extremera, V.; Jiménez-Legido, M.; Duat-Rodríguez, A.; García-Fernández, M.; Ortiz-Cabrera, N.V.; Ruiz-Falcó-Rojas, M.L.; González-Gutiérrez-Solana, L. Tocilizumab in pediatric refractory status epilepticus and acute epilepsy: Experience in two patients. *Journal of neuroimmunology* **2020**, *340*, 577142, doi:10.1016/j.jneuroim.2019.577142.

434. Zhou, W.Z.; Zhang, J.; Li, Z.; Lin, X.; Li, J.; Wang, S.; Yang, C.; Wu, Q.; Ye, A.Y.; Wang, M.; et al. Targeted resequencing of 358 candidate genes for autism spectrum disorder in a Chinese cohort reveals diagnostic potential and genotype-phenotype correlations. *Human mutation* **2019**, *40*, 801-815, doi:10.1002/humu.23724.

435. Jiang, P.; Zhu, W.Y.; He, X.; Tang, M.M.; Dang, R.L.; Li, H.D.; Xue, Y.; Zhang, L.H.; Wu, Y.Q.; Cao, L.J. Association between Vitamin D Receptor Gene Polymorphisms with Childhood Temporal Lobe Epilepsy. *International journal of environmental research and public health* **2015**, *12*, 13913-13922, doi:10.3390/ijerph121113913.

436. Munisamy, M.; Tripathi, M.; Behari, M.; Raghavan, S.; Jain, D.C.; Ramanujam, B.; Arumugam, K.; Rajakannan, T.; Mallayasamy, S.R.; Subbiah, V. The effect of uridine diphosphate glucuronosyltransferase (UGT)1A6 genetic polymorphism on valproic acid pharmacokinetics in Indian patients with epilepsy: a pharmacogenetic approach. *Molecular diagnosis & therapy* **2013**, *17*, 319-326, doi:10.1007/s40291-013-0041-8.

437. Landa, P.; Differ, A.M.; Rajput, K.; Jenkins, L.; Bitner-Glindzicz, M. Lack of significant association between mutations of KCNJ10 or FOXI1 and SLC26A4 mutations in Pendred syndrome/enlarged vestibular aqueducts. *BMC medical genetics* **2013**, *14*, 85, doi:10.1186/1471-2350-14-85.

438. Du, Z.; Xu, H.; Zhao, P.; Wang, J.; Xu, Q.; Liu, M. Influence of UGT2B7 and UGT1A6 polymorphisms on plasma concentration to dose ratio of valproic acid in Chinese epileptic children. *Xenobiotica; the fate of foreign compounds in biological systems* **2021**, *51*, 859-864, doi:10.1080/00498254.2021.1931554.

439. Conti, S.; Condò, M.; Posar, A.; Mari, F.; Resta, N.; Renieri, A.; Neri, I.; Patrizi, A.; Parmeggiani, A. Phosphatase and tensin homolog (PTEN) gene mutations and autism: literature review and

a case report of a patient with Cowden syndrome, autistic disorder, and epilepsy. *Journal of child neurology* **2012**, *27*, 392-397, doi:10.1177/0883073811420296.
440. Labate, A.; Manna, I.; Gambardella, A.; Le Piane, E.; La Russa, A.; Condino, F.; Cittadella, R.; Aguglia, U.; Quattrone, A. Association between the M129V variant allele of PRNP gene and mild temporal lobe epilepsy in women. *Neuroscience letters* **2007**, *421*, 1-4, doi:10.1016/j.neulet.2006.10.020.
441. Valadão, M.N.; Coimbra, E.R.; Landemberger, M.C.; Velasco, T.R.; Terra, V.C.; Wichert-Ana, L.; Alexandre, V., Jr.; Araújo, D., Jr.; Guarnieri, R.; Martins, V.R.; et al. Prnp gene and cerebellum volume in patients with refractory mesial temporal lobe epilepsy. *Neurological sciences : official journal of the Italian Neurological Society and of the Italian Society of Clinical Neurophysiology* **2014**, *35*, 239-244, doi:10.1007/s10072-013-1494-6.
442. Ortega-Vázquez, A.; Fricke-Galindo, I.; Dorado, P.; Jung-Cook, H.; Martínez-Juárez, I.E.; Monroy-Jaramillo, N.; Rojas-Tomé, I.S.; Peñas-Lledó, E.; Llerena, A.; López-López, M. Influence of genetic variants and antiepileptic drug co-treatment on lamotrigine plasma concentration in Mexican Mestizo patients with epilepsy. *The pharmacogenomics journal* **2020**, *20*, 845-856, doi:10.1038/s41397-020-0173-2.
443. Karatoprak, E.; Sozen, G.; Yılmaz, K.; Ozer, I. Interictal epileptiform discharges on electroencephalography in children with methylenetetrahydrofolate reductase (MTHFR) polymorphisms. *Neurological sciences : official journal of the Italian Neurological Society and of the Italian Society of Clinical Neurophysiology* **2020**, *41*, 631-636, doi:10.1007/s10072-019-04119-4.
444. Al-Eitan, L.N.; Al-Dalala, I.M.; Elshammari, A.K.; Khreisat, W.H.; Nimiri, A.F.; Alnaamneh, A.H.; Aljamal, H.A.; Alghamdi, M.A. Genetic Association of Epilepsy and Anti-Epileptic Drugs Treatment in Jordanian Patients. *Pharmacogenomics and personalized medicine* **2020**, *13*, 503-510, doi:10.2147/pgpm.s273125.
445. Langeh, U.; Chawla, P.; Gupta, G.D.; Singh, S. A Novel Approach to Refractory Epilepsy by Targeting Pgp Peripherally and Centrally: Therapeutic Targets and Future Perspectives. *CNS & neurological disorders drug targets* **2020**, *19*, 741-749, doi:10.2174/1871527319999200819093109.
446. Lv, R.J.; He, J.S.; Fu, Y.H.; Shao, X.Q.; Wu, L.W.; Lu, Q.; Jin, L.R.; Liu, H. A polymorphism in CALHM1 is associated with temporal lobe epilepsy. *Epilepsy & behavior : E&B* **2011**, *20*, 681-685, doi:10.1016/j.yebeh.2011.02.007.
447. Li, X.; Wang, Y.; Gu, J.; Meng, Q.; Gao, Y.; Zhao, H.; Yin, Z. No association between polymorphisms in the calcium homeostasis modulator 1 gene and mesial temporal lobe epilepsy risk in a Chinese population. *Seizure* **2014**, *23*, 231-233, doi:10.1016/j.seizure.2013.11.010.
448. Horta, W.G.; Paradela, E.; Figueiredo, A.; Meira, I.D.; Pereira, V.C.; Rego, C.C.; Oliveira, R.; Andraus, M.E.; de Lacerda, G.C.; Moura, P.; et al. Genetic association study of the HLA class II alleles DRB1, DQA1, and DQB1 in patients with pharmacoresistant temporal lobe epilepsy associated with mesial hippocampal sclerosis. *Seizure* **2015**, *31*, 7-11, doi:10.1016/j.seizure.2015.06.005.
449. Zhao, T.; Li, H.J.; Wang, T.T.; Bahatibieke, M.; Jia, L.; Wang, F.; Liu, W.L.; Ji, Y.; Sun, L.; Sun, Y.; et al. Association between HLA genotype and antiseizure medications (ASMs)-induced maculopapular eruption among epilepsy patients in Xinjiang, China. *Epilepsy Res* **2020**, *165*,

106391, doi:10.1016/j.eplepsyres.2020.106391.

450. Yoneda, M.; Tanno, Y.; Horai, S.; Ozawa, T.; Miyatake, T.; Tsuji, S. A common mitochondrial DNA mutation in the t-RNA(Lys) of patients with myoclonus epilepsy associated with ragged-red fibers. *Biochemistry international* **1990**, *21*, 789-796.

451. Blok, M.J.; Spruijt, L.; de Coo, I.F.; Schoonderwoerd, K.; Hendrickx, A.; Smeets, H.J. Mutations in the ND5 subunit of complex I of the mitochondrial DNA are a frequent cause of oxidative phosphorylation disease. *Journal of medical genetics* **2007**, *44*, e74, doi:10.1136/jmg.2006.045716.

452. Li, Y.; Wang, J.; Jiang, C.; Zheng, G.; Lu, X.; Guo, H. Association of the genetic polymorphisms in pre-microRNAs with risk of childhood epilepsy in a Chinese population. *Seizure* **2016**, *40*, 21-26, doi:10.1016/j.seizure.2016.04.011.

453. Heron, S.E.; Ong, Y.S.; Yendle, S.C.; McMahon, J.M.; Berkovic, S.F.; Scheffer, I.E.; Dibbens, L.M. Mutations in PRRT2 are not a common cause of infantile epileptic encephalopathies. *Epilepsia* **2013**, *54*, e86-89, doi:10.1111/epi.12167.

454. Marotta, R.; Chin, J.; Quigley, A.; Katsabanis, S.; Kapsa, R.; Byrne, E.; Collins, S. Diagnostic screening of mitochondrial DNA mutations in Australian adults 1990-2001. *Internal medicine journal* **2004**, *34*, 10-19, doi:10.1111/j.1444-0903.2004.t01-3-.x.

455. Vanlerberghe, C.; Petit, F.; Malan, V.; Vincent-Delorme, C.; Bouquillon, S.; Boute, O.; Holder-Espinasse, M.; Delobel, B.; Duban, B.; Vallee, L.; et al. 15q11.2 microdeletion (BP1-BP2) and developmental delay, behaviour issues, epilepsy and congenital heart disease: a series of 52 patients. *European journal of medical genetics* **2015**, *58*, 140-147, doi:10.1016/j.ejmg.2015.01.002.

456. Fry, A.E.; Rees, E.; Thompson, R.; Mantripragada, K.; Blake, P.; Jones, G.; Morgan, S.; Jose, S.; Mugalaasi, H.; Archer, H.; et al. Pathogenic copy number variants and SCN1A mutations in patients with intellectual disability and childhood-onset epilepsy. *BMC medical genetics* **2016**, *17*, 34, doi:10.1186/s12881-016-0294-2.

457. He, X.J.; Jian, L.Y.; He, X.L.; Tang, M.; Wu, Y.; Xu, Y.Y.; Sun, X.J.; Zhao, L.M. Association of ABCB1, CYP3A4, EPHX1, FAS, SCN1A, MICA, and BAG6 polymorphisms with the risk of carbamazepine-induced Stevens-Johnson syndrome/toxic epidermal necrolysis in Chinese Han patients with epilepsy. *Epilepsia* **2014**, *55*, 1301-1306, doi:10.1111/epi.12655.

458. Suzuki, T.; Koike, Y.; Ashikawa, K.; Otomo, N.; Takahashi, A.; Aoi, T.; Kamatani, N.; Nakamura, Y.; Kubo, M.; Kamatani, Y.; et al. Genome-wide association study of epilepsy in a Japanese population identified an associated region at chromosome 12q24. *Epilepsia* **2021**, *62*, 1391-1400, doi:10.1111/epi.16911.

459. Cario, H.; Smith, D.E.; Blom, H.; Blau, N.; Bode, H.; Holzmann, K.; Pannicke, U.; Hopfner, K.P.; Rump, E.M.; Ayric, Z.; et al. Dihydrofolate reductase deficiency due to a homozygous DHFR mutation causes megaloblastic anemia and cerebral folate deficiency leading to severe neurologic disease. *American journal of human genetics* **2011**, *88*, 226-231, doi:10.1016/j.ajhg.2011.01.007.

460. Reimers, A.; Østby, L.; Stuen, I.; Sundby, E. Expression of UDP-glucuronosyltransferase 1A4 in human placenta at term. *European journal of drug metabolism and pharmacokinetics* **2011**, *35*, 79-82, doi:10.1007/s13318-010-0021-x.

461. Ghosh, C.; Hossain, M.; Puvenna, V.; Martinez-Gonzalez, J.; Alexopolous, A.; Janigro, D.; Marchi, N. Expression and functional relevance of UGT1A4 in a cohort of human drug-resistant epileptic

brains. *Epilepsia* **2013**, *54*, 1562-1570, doi:10.1111/epi.12318.

462. Lu, Y.; Fang, Y.; Wu, X.; Ma, C.; Wang, Y.; Xu, L. Effects of UGT1A9 genetic polymorphisms on monohydroxylated derivative of oxcarbazepine concentrations and oxcarbazepine monotherapeutic efficacy in Chinese patients with epilepsy. *European journal of clinical pharmacology* **2017**, *73*, 307-315, doi:10.1007/s00228-016-2157-3.

463. Chu, X.M.; Zhang, L.F.; Wang, G.J.; Zhang, S.N.; Zhou, J.H.; Hao, H.P. Influence of UDP-glucuronosyltransferase polymorphisms on valproic acid pharmacokinetics in Chinese epilepsy patients. *European journal of clinical pharmacology* **2012**, *68*, 1395-1401, doi:10.1007/s00228-012-1277-7.

464. Mei, S.; Feng, W.; Zhu, L.; Li, X.; Yu, Y.; Yang, W.; Gao, B.; Wu, X.; Fang, F.; Zhao, Z. Effect of CYP2C19, UGT1A8, and UGT2B7 on valproic acid clearance in children with epilepsy: a population pharmacokinetic model. *European journal of clinical pharmacology* **2018**, *74*, 1029-1036, doi:10.1007/s00228-018-2440-6.

465. Lin, L.; Zhang, Y.; Pan, H.; Wang, J.; Qi, Y.; Ma, Y. Clinical and genetic characteristics and prenatal diagnosis of patients presented GDD/ID with rare monogenic causes. *Orphanet journal of rare diseases* **2020**, *15*, 317, doi:10.1186/s13023-020-01599-y.

466. Córdoba, M.; Consalvo, D.; Moron, D.G.; Kochen, S.; Kauffman, M.A. SLC6A4 gene variants and temporal lobe epilepsy susceptibility: a meta-analysis. *Molecular biology reports* **2012**, *39*, 10615-10619, doi:10.1007/s11033-012-1949-5.

467. Kauffman, M.A.; Consalvo, D.; Gonzalez-Morón, D.; Aguirre, F.; D'Alessio, L.; Kochen, S. Serotonin transporter gene variation and refractory mesial temporal epilepsy with hippocampal sclerosis. *Epilepsy Res* **2009**, *85*, 231-234, doi:10.1016/j.eplepsyres.2009.03.010.

468. Singh, N.A.; Pappas, C.; Dahle, E.J.; Claes, L.R.; Pruess, T.H.; De Jonghe, P.; Thompson, J.; Dixon, M.; Gurnett, C.; Peiffer, A.; et al. A role of SCN9A in human epilepsies, as a cause of febrile seizures and as a potential modifier of Dravet syndrome. *PLoS genetics* **2009**, *5*(9):e1000649, doi:10.1371/journal.pgen.1000649.

469. Fasham, J.; Leslie, J.S.; Harrison, J.W.; Deline, J.; Williams, K.B.; Kuhl, A.; Scott Schwoerer, J.; Cross, H.E.; Crosby, A.H.; Baple, E.L. No association between SCN9A and monogenic human epilepsy disorders. *PLoS genetics* **2020**, *16*, e1009161, doi:10.1371/journal.pgen.1009161.

470. Muhle, H.; von Spiczak, S.; Gaus, V.; Kara, S.; Helbig, I.; Hampe, J.; Franke, A.; Weber, Y.; Lerche, H.; Kleefuss-Lie, A.A.; et al. Role of GRM4 in idiopathic generalized epilepsies analysed by genetic association and sequence analysis. *Epilepsy Res* **2010**, *89*, 319-326, doi:10.1016/j.eplepsyres.2010.02.004.

471. Izzi, C.; Barbon, A.; Toliat, M.R.; Heils, A.; Becker, C.; Nürnberg, P.; Sander, T.; Barlati, S. Candidate gene analysis of the human metabotropic glutamate receptor type 4 (GRM4) in patients with juvenile myoclonic epilepsy. *American journal of medical genetics. Part B, Neuropsychiatric genetics : the official publication of the International Society of Psychiatric Genetics* **2003**, *123b*, 59-63, doi:10.1002/ajmg.b.20024.

472. Al-Eitan, L.N.; Al-Dalalah, I.M.; Aljamal, H.A. Effects of GRM4, SCN2A and SCN3B polymorphisms on antiepileptic drugs responsiveness and epilepsy susceptibility. *Saudi pharmaceutical journal : SPJ : the official publication of the Saudi Pharmaceutical Society* **2019**, *27*, 731-737, doi:10.1016/j.jsps.2019.04.009.

473. Semmler, A.; Moskau-Hartmann, S.; Stoffel-Wagner, B.; Elger, C.; Linnebank, M. Homocysteine plasma levels in patients treated with antiepileptic drugs depend on folate and vitamin B12

serum levels, but not on genetic variants of homocysteine metabolism. *Clinical chemistry and laboratory medicine* **2013**, *51*, 665-669, doi:10.1515/cclm-2012-0580.
474. Delev, D.; Pavlova, A.; Grote, A.; Boström, A.; Höllig, A.; Schramm, J.; Fimmers, R.; Oldenburg, J.; Simon, M. NOTCH4 gene polymorphisms as potential risk factors for brain arteriovenous malformation development and hemorrhagic presentation. *Journal of neurosurgery* **2017**, *126*, 1552-1559, doi:10.3171/2016.3.jns151731.
475. Han, P.; Wang, X.F.; Wang, L. [Adenosine triphosphate-binding gene messenger ribonucleic acid expression in brains of drug-resistant epileptics]. *Zhonghua yi xue za zhi* **2011**, *91*, 2314-2318.
476. Wen, Z.P.; Fan, S.S.; Du, C.; Yin, T.; Zhou, B.T.; Peng, Z.F.; Xie, Y.Y.; Zhang, W.; Chen, Y.; Tang, J.; et al. Influence of acylpeptide hydrolase polymorphisms on valproic acid level in Chinese epilepsy patients. *Pharmacogenomics* **2016**, *17*, 1219-1225, doi:10.2217/pgs-2016-0030.
477. Edvardson, S.; Nicolae, C.M.; Noh, G.J.; Burton, J.E.; Punzi, G.; Shaag, A.; Bischetsrieder, J.; De Grassi, A.; Pierri, C.L.; Elpeleg, O.; et al. Heterozygous RNF13 Gain-of-Function Variants Are Associated with Congenital Microcephaly, Epileptic Encephalopathy, Blindness, and Failure to Thrive. *American journal of human genetics* **2019**, *104*, 179-185, doi:10.1016/j.ajhg.2018.11.018.
478. Esih, K.; Goričar, K.; Dolžan, V.; Rener-Primec, Z. Antioxidant polymorphisms do not influence the risk of epilepsy or its drug resistance after neonatal hypoxic-ischemic brain injury. *Seizure* **2017**, *46*, 38-42, doi:10.1016/j.seizure.2017.01.005.
479. Esih, K.; Goričar, K.; Dolžan, V.; Rener-Primec, Z. The association between antioxidant enzyme polymorphisms and cerebral palsy after perinatal hypoxic-ischaemic encephalopathy. *European journal of paediatric neurology : EJPN : official journal of the European Paediatric Neurology Society* **2016**, *20*, 704-708, doi:10.1016/j.ejpn.2016.05.018.
480. Santos, B.; Marques, T.; Malta, M.; Gameleira, F.; Secolin, R.; Andrade, T.; Gitaí, L.; Gitaí, D. PER2 rs2304672, CLOCK rs1801260, and PER3 rs57875989 polymorphisms are not associated with juvenile myoclonic epilepsy. *Epilepsy & behavior : E&B* **2014**, *36*, 82-85, doi:10.1016/j.yebeh.2014.04.024.
481. Lohoff, F.W.; Ferraro, T.N.; Sander, T.; Zhao, H.; Dahl, J.P.; Berrettini, W.H.; Buono, R.J. No association between common variations in the human alpha 2 subunit gene (ATP1A2) of the sodium-potassium-transporting ATPase and idiopathic generalized epilepsy. *Neuroscience letters* **2005**, *382*, 33-38, doi:10.1016/j.neulet.2005.02.057.
482. Dean, J.C.; Robertson, Z.; Reid, V.; Wang, Q.; Hailey, H.; Moore, S.; Rasalam, A.D.; Turnpenny, P.; Lloyd, D.; Shaw, D.; et al. A high frequency of the MTHFR 677C>T polymorphism in Scottish women with epilepsy: possible role in pathogenesis. *Seizure* **2008**, *17*, 269-275, doi:10.1016/j.seizure.2007.08.003.
483. Daci, A.; Beretta, G.; Vllasaliu, D.; Shala, A.; Govori, V.; Norata, G.D.; Krasniqi, S. Polymorphic Variants of SCN1A and EPHX1 Influence Plasma Carbamazepine Concentration, Metabolism and Pharmacoresistance in a Population of Kosovar Albanian Epileptic Patients. *PloS one* **2015**, *10*(11):e0142408, doi:10.1371/journal.pone.0142408.
484. Chbili, C.; Fathallah, N.; Laouani, A.; Nouira, M.; Hassine, A.; Ben Amor, S.; Ben Ammou, S.; Ben Salem, C.; Saguem, S. Effects of EPHX1 and CYP3A4*22 genetic polymorphisms on carbamazepine metabolism and drug response among Tunisian epileptic patients. *Journal of neurogenetics* **2016**, *30*, 16-21, doi:10.3109/01677063.2016.1155571.
485. Balan, S.; Bharathan, S.P.; Vellichiramal, N.N.; Sathyan, S.; Joseph, V.; Radhakrishnan, K.;

Banerjee, M. Genetic association analysis of ATP binding cassette protein family reveals a novel association of ABCB1 genetic variants with epilepsy risk, but not with drug-resistance. *PloS one* **2014**, *9*, e89253, doi:10.1371/journal.pone.0089253.

486. Al-Eitan, L.N.; Al-Dalalah, I.M.; Mustafa, M.M.; Alghamdi, M.A.; Elshammari, A.K.; Khreisat, W.H.; Al-Quasmi, M.N.; Aljamal, H.A. Genetic polymorphisms of CYP3A5, CHRM2, and ZNF498 and their association with epilepsy susceptibility: a pharmacogenetic and case-control study. *Pharmacogenomics and personalized medicine* **2019**, *12*, 225-233, doi:10.2147/pgpm.s212433.

487. Abo El Fotoh, W.M.; Abd El Naby, S.A.; Habib, M.S.; AA, A.L.; Kasemy, Z.A. The potential implication of SCN1A and CYP3A5 genetic variants on antiepileptic drug resistance among Egyptian epileptic children. *Seizure* **2016**, *41*, 75-80, doi:10.1016/j.seizure.2016.07.005.

488. Kwan, P.; Poon, W.S.; Ng, H.K.; Kang, D.E.; Wong, V.; Ng, P.W.; Lui, C.H.; Sin, N.C.; Wong, K.S.; Baum, L. Multidrug resistance in epilepsy and polymorphisms in the voltage-gated sodium channel genes SCN1A, SCN2A, and SCN3A: correlation among phenotype, genotype, and mRNA expression. *Pharmacogenetics and genomics* **2008**, *18*, 989-998, doi:10.1097/FPC.0b013e3283117d67.

489. Haerian, B.S.; Baum, L.; Kwan, P.; Tan, H.J.; Raymond, A.A.; Mohamed, Z. SCN1A, SCN2A and SCN3A gene polymorphisms and responsiveness to antiepileptic drugs: a multicenter cohort study and meta-analysis. *Pharmacogenomics* **2013**, *14*, 1153-1166, doi:10.2217/pgs.13.104.

490. Heyne, H.O.; Artomov, M.; Battke, F.; Bianchini, C.; Smith, D.R.; Liebmann, N.; Tadigotla, V.; Stanley, C.M.; Lal, D.; Rehm, H.; et al. Targeted gene sequencing in 6994 individuals with neurodevelopmental disorder with epilepsy. *Genetics in medicine : official journal of the American College of Medical Genetics* **2019**, *21*, 2496-2503, doi:10.1038/s41436-019-0531-0.

491. Stogmann, E.; Lichtner, P.; Baumgartner, C.; Schmied, M.; Hotzy, C.; Asmus, F.; Leutmezer, F.; Bonelli, S.; Assem-Hilger, E.; Vass, K.; et al. Mutations in the CLCN2 gene are a rare cause of idiopathic generalized epilepsy syndromes. *Neurogenetics* **2006**, *7*, 265-268, doi:10.1007/s10048-006-0057-x.

492. Djordjevic, N.; Milovanovic, D.D.; Radovanovic, M.; Radosavljevic, I.; Obradovic, S.; Jakovljevic, M.; Milovanovic, D.; Milovanovic, J.R.; Jankovic, S. CYP1A2 genotype affects carbamazepine pharmacokinetics in children with epilepsy. *European journal of clinical pharmacology* **2016**, *72*, 439-445, doi:10.1007/s00228-015-2006-9.

493. Cabral-Pereira, G.; Sánchez-Benito, D.; Díaz-Rodríguez, S.M.; Gonçalves, J.; Sancho, C.; Castellano, O.; Muñoz, L.J.; López, D.E.; Gómez-Nieto, R. Behavioral and Molecular Effects Induced by Cannabidiol and Valproate Administration in the GASH/Sal Model of Acute Audiogenic Seizures. *Frontiers in behavioral neuroscience* **2020**, *14*, 612624, doi:10.3389/fnbeh.2020.612624.

494. Flanagan, S.E.; Edghill, E.L.; Gloyn, A.L.; Ellard, S.; Hattersley, A.T. Mutations in KCNJ11, which encodes Kir6.2, are a common cause of diabetes diagnosed in the first 6 months of life, with the phenotype determined by genotype. *Diabetologia* **2006**, *49*, 1190-1197, doi:10.1007/s00125-006-0246-z.

495. Jang, Y.; Kim, T.J.; Moon, J.; Yang, T.W.; Kim, K.T.; Park, B.S.; Lim, J.A.; Jun, J.S.; Lee, S.T.; Jung, K.H.; et al. HLAs associated with perampanel-induced psychiatric adverse effects in a Korean population. *Scientific reports* **2020**, *10*, 13667, doi:10.1038/s41598-020-70601-1.

496. Depondt, C.; Cock, H.R.; Healy, D.G.; Burley, M.W.; Weinshenker, D.; Wood, N.W.; Goldstein, D.B.; Sisodiya, S.M. The -1021C->T DBH gene variant is not associated with epilepsy or

antiepileptic drug response. *Neurology* **2004**, *63*, 1497-1499, doi:10.1212/01.wnl.0000142092.16719.ad.
497. Helmstaedter, C.; Mihov, Y.; Toliat, M.R.; Thiele, H.; Nuernberg, P.; Schoch, S.; Surges, R.; Elger, C.E.; Kunz, W.S.; Hurlemann, R. Genetic variation in dopaminergic activity is associated with the risk for psychiatric side effects of levetiracetam. *Epilepsia* **2013**, *54*, 36-44, doi:10.1111/j.1528-1167.2012.03603.x.
498. Bhat, M.A.; Guru, S.A.; Mir, R.; Waza, A.A.; Zuberi, M.; Sumi, M.P.; Bodeliwala, S.; Puri, V.; Saxena, A. Association of GABAA Receptor Gene with Epilepsy Syndromes. *Journal of molecular neuroscience : MN* **2018**, *65*, 141-153, doi:10.1007/s12031-018-1081-7.
499. Chen, J.; Su, Q.; Qin, J.; Zhou, Y.; Ruan, H.; Chen, Z.; Chen, Z.; Li, H.; Zhou, Y.; Zhou, S.; et al. Correlation of MCT1 and ABCC2 gene polymorphisms with valproic acid resistance in patients with epilepsy on valproic acid monotherapy. *Drug metabolism and pharmacokinetics* **2019**, *34*, 165-171, doi:10.1016/j.dmpk.2018.01.006.
500. Fricke-Galindo, I.; Ortega-Vázquez, A.; Monroy-Jaramillo, N.; Dorado, P.; Jung-Cook, H.; Peñas-Lledó, E.; A, L.L.; López-López, M. Allele and genotype frequencies of genes relevant to anti-epileptic drug therapy in Mexican-Mestizo healthy volunteers. *Pharmacogenomics* **2016**, *17*, 1913-1930, doi:10.2217/pgs-2016-0078.
501. Kotlarz, D.; Marquardt, B.; Barøy, T.; Lee, W.S.; Konnikova, L.; Hollizeck, S.; Magg, T.; Lehle, A.S.; Walz, C.; Borggraefe, I.; et al. Human TGF-β1 deficiency causes severe inflammatory bowel disease and encephalopathy. *Nature genetics* **2018**, *50*, 344-348, doi:10.1038/s41588-018-0063-6.
502. Dong, X.; Tan, N.B.; Howell, K.B.; Barresi, S.; Freeman, J.L.; Vecchio, D.; Piccione, M.; Radio, F.C.; Calame, D.; Zong, S.; et al. Bi-allelic LoF NRROS Variants Impairing Active TGF-β1 Delivery Cause a Severe Infantile-Onset Neurodegenerative Condition with Intracranial Calcification. *American journal of human genetics* **2020**, *106*, 559-569, doi:10.1016/j.ajhg.2020.02.014.
503. Soares, R.V.; Do, T.M.; Mabondzo, A.; Pons, G.; Chhun, S. Ontogeny of ABC and SLC transporters in the microvessels of developing rat brain. *Fundamental & clinical pharmacology* **2016**, *30*, 107-116, doi:10.1111/fcp.12175.
504. Shah, P.; Demirbilek, H.; Hussain, K. Persistent hyperinsulinaemic hypoglycaemia in infancy. *Seminars in pediatric surgery* **2014**, *23*, 76-82, doi:10.1053/j.sempedsurg.2014.03.005.
505. Haerian, B.S.; Sha'ari, H.M.; Fong, C.Y.; Tan, H.J.; Wong, S.W.; Ong, L.C.; Raymond, A.A.; Tan, C.T.; Mohamed, Z. Contribution of TIMP4 rs3755724 polymorphism to susceptibility to focal epilepsy in Malaysian Chinese. *Journal of neuroimmunology* **2015**, *278*, 137-143, doi:10.1016/j.jneuroim.2014.12.016.
506. Lu, Y.; Yu, W.; Xi, Z.; Xiao, Z.; Kou, X.; Wang, X.F. Mutational analysis of SCN2B, SCN3B and SCN4B in a large Chinese Han family with generalized tonic-clonic seizure. *Neurological sciences : official journal of the Italian Neurological Society and of the Italian Society of Clinical Neurophysiology* **2010**, *31*, 675-677, doi:10.1007/s10072-010-0390-6.
507. Wang, J.; Zhang, Y.; Liang, J.; Pan, H.; Wu, H.; Xu, K.; Liu, X.; Jiang, Y.; Shen, Y.; Wu, X. CACNA1I is not associated with childhood absence epilepsy in the Chinese Han population. *Pediatric neurology* **2006**, *35*, 187-190, doi:10.1016/j.pediatrneurol.2006.03.006.
508. Bai, X.; Xu, C.; Wen, D.; Chen, Y.; Li, H.; Wang, X.; Zhou, L.; Huang, M.; Jin, J. Polymorphisms of peroxisome proliferator-activated receptor γ (PPARγ) and cluster of differentiation 36 (CD36) associated with valproate-induced obesity in epileptic patients. *Psychopharmacology* **2018**,

235, 2665-2673, doi:10.1007/s00213-018-4960-2.
509. Nava, C.; Rupp, J.; Boissel, J.P.; Mignot, C.; Rastetter, A.; Amiet, C.; Jacquette, A.; Dupuits, C.; Bouteiller, D.; Keren, B.; et al. Hypomorphic variants of cationic amino acid transporter 3 in males with autism spectrum disorders. *Amino acids* **2015**, *47*, 2647-2658, doi:10.1007/s00726-015-2057-3.
510. Berrin, T.; Hikmet, Y.; Gülşen, V.; Ferda, B.; Erdal, B.; Ece, O. No relation between EFHC2 gene polymorphism and Idiopathic generalized epilepsy. *African health sciences* **2015**, *15*, 1204-1210, doi:10.4314/ahs.v15i4.20.
511. Gu, W.; Sander, T.; Heils, A.; Lenzen, K.P.; Steinlein, O.K. A new EF-hand containing gene EFHC2 on Xp11.4: tentative evidence for association with juvenile myoclonic epilepsy. *Epilepsy Res* **2005**, *66*, 91-98, doi:10.1016/j.eplepsyres.2005.07.003.
512. Schoeler, N.E.; Leu, C.; White, J.; Plagnol, V.; Ellard, S.; Matarin, M.; Yellen, G.; Thiele, E.A.; Mackay, M.; McMahon, J.M.; et al. Variants in KCNJ11 and BAD do not predict response to ketogenic dietary therapies for epilepsy. *Epilepsy Res* **2015**, *118*, 22-28, doi:10.1016/j.eplepsyres.2015.10.003.
513. Barone, R.; Carrozzi, M.; Parini, R.; Battini, R.; Martinelli, D.; Elia, M.; Spada, M.; Lilliu, F.; Ciana, G.; Burlina, A.; et al. A nationwide survey of PMM2-CDG in Italy: high frequency of a mild neurological variant associated with the L32R mutation. *Journal of neurology* **2015**, *262*, 154-164, doi:10.1007/s00415-014-7549-7.
514. Tropeano, M.; Ahn, J.W.; Dobson, R.J.; Breen, G.; Rucker, J.; Dixit, A.; Pal, D.K.; McGuffin, P.; Farmer, A.; White, P.S.; et al. Male-biased autosomal effect of 16p13.11 copy number variation in neurodevelopmental disorders. *PloS one* **2013**, *8*(4):e61365, doi:10.1371/journal.pone.0061365.
515. Nishimura, Y.V.; Sekine, K.; Chihama, K.; Nakajima, K.; Hoshino, M.; Nabeshima, Y.; Kawauchi, T. Dissecting the factors involved in the locomotion mode of neuronal migration in the developing cerebral cortex. *The Journal of biological chemistry* **2010**, *285*, 5878-5887, doi:10.1074/jbc.M109.033761.
516. Leschziner, G.; Jorgensen, A.L.; Andrew, T.; Pirmohamed, M.; Williamson, P.R.; Marson, A.G.; Coffey, A.J.; Middleditch, C.; Rogers, J.; Bentley, D.R.; et al. Clinical factors and ABCB1 polymorphisms in prediction of antiepileptic drug response: a prospective cohort study. *The Lancet. Neurology* **2006**, *5*, 668-676, doi:10.1016/s1474-4422(06)70500-2.
517. Ritter, A.C.; Kammerer, C.M.; Brooks, M.M.; Conley, Y.P.; Wagner, A.K. Genetic variation in neuronal glutamate transport genes and associations with posttraumatic seizure. *Epilepsia* **2016**, *57*, 984-993, doi:10.1111/epi.13397.
518. Mishra, P.K.; Teale, J.M. Transcriptome analysis of the ependymal barrier during murine neurocysticercosis. *Journal of neuroinflammation* **2012**, *9*, 141, doi:10.1186/1742-2094-9-141.
519. Nishiyama, M.; Nakamichi, N.; Yoshimura, T.; Masuo, Y.; Komori, T.; Ishimoto, T.; Matsuo, J.I.; Kato, Y. Homostachydrine is a Xenobiotic Substrate of OCTN1/SLC22A4 and Potentially Sensitizes Pentylenetetrazole-Induced Seizures in Mice. *Neurochemical research* **2020**, *45*, 2664-2678, doi:10.1007/s11064-020-03118-8.
520. Bizec, C.L.; Nicole, S.; Panagiotakaki, E.; Seta, N.; Vuillaumier-Barrot, S. No Mutation in the SLC2A3 Gene in Cohorts of GLUT1 Deficiency Syndrome-Like Patients Negative for SLC2A1 and in Patients with AHC Negative for ATP1A3. *JIMD reports* **2014**, *12*, 115-120, doi:10.1007/8904_2013_253.

521. Villegas-Martínez, I.; de-Miguel-Elízaga, I.; Carrasco-Torres, R.; Marras, C.; Canteras-Jordana, M.; Yedra-Guzmán, M.J.; Martínez-Villanueva, M.; Tortosa-Conesa, D.; Martín-Fernández, J. The COL1A1 SP1 polymorphism is associated with lower bone mineral density in patients treated with valproic acid. *Pharmacogenetics and genomics* **2016**, *26*, 126-132, doi:10.1097/fpc.0000000000000199.

522. Balcerzyk, A.; Niemiec, P.; Iwanicki, T.; Nowak, T.; Kopyta, I.; Emich-Widera, E.; Pilarska, E.; Pienczk-Ręcławowicz, K.; Kaciński, M.; Wendorff, J.; et al. Upstream Stimulating Factor 1 (USF-1) Gene Polymorphisms and the Risk, Symptoms, and Outcome of Pediatric Ischemic Stroke. *Journal of stroke and cerebrovascular diseases : the official journal of National Stroke Association* **2018**, *27*, 1885-1889, doi:10.1016/j.jstrokecerebrovasdis.2018.02.034.

523. Haerian, B.; Mohd Sha'ari, H.; Fong, C.Y.; Tan, H.; Wong, S.; Choong, O.; Ali, R.; Tan, C.; Mohamed, Z. Contribution of TIMP4 rs3755724 polymorphism to susceptibility to focal epilepsy in Malaysian Chinese. *Journal of neuroimmunology* **2014**, *278*, doi:10.1016/j.jneuroim.2014.12.016.

524. von Spiczak, S.; Muhle, H.; Helbig, I.; de Kovel, C.G.; Hampe, J.; Gaus, V.; Koeleman, B.P.; Lindhout, D.; Schreiber, S.; Sander, T.; et al. Association study of TRPC4 as a candidate gene for generalized epilepsy with photosensitivity. *Neuromolecular medicine* **2010**, *12*, 292-299, doi:10.1007/s12017-010-8122-x.

525. Bose, S.; He, H.; Stauber, T. Neurodegeneration Upon Dysfunction of Endosomal/Lysosomal CLC Chloride Transporters. *Frontiers in cell and developmental biology* **2021**, *9*, 639231, doi:10.3389/fcell.2021.639231.

526. Alrayes, N.; Mohamoud, H.S.; Jelani, M.; Ahmad, S.; Vadgama, N.; Bakur, K.; Simpson, M.; Al-Aama, J.Y.; Nasir, J. Truncating mutation in intracellular phospholipase A_1 gene (DDHD2) in hereditary spastic paraplegia with intellectual disability (SPG54). *BMC research notes* **2015**, *8*, 271, doi:10.1186/s13104-015-1227-4.

527. Marchese, M.; Conti, V.; Valvo, G.; Moro, F.; Muratori, F.; Tancredi, R.; Santorelli, F.M.; Guerrini, R.; Sicca, F. Autism-epilepsy phenotype with macrocephaly suggests PTEN, but not GLIALCAM, genetic screening. *BMC medical genetics* **2014**, *15*, 26, doi:10.1186/1471-2350-15-26.

528. Song, L.L.; Wang, Y.M.; Wu, X.X.; Zhu, L.X.; Pan, F.; Chen, Y.M. Correlation between AT1R gene polymorphism and epilepsy secondary to cerebral infarction. *European review for medical and pharmacological sciences* **2020**, *24*, 6873-6880, doi:10.26355/eurrev_202006_21677.

529. Leger, P.L.; Souville, I.; Boddaert, N.; Elie, C.; Pinard, J.M.; Plouin, P.; Moutard, M.L.; des Portes, V.; Van Esch, H.; Joriot, S.; et al. The location of DCX mutations predicts malformation severity in X-linked lissencephaly. *Neurogenetics* **2008**, *9*, 277-285, doi:10.1007/s10048-008-0141-5.

530. Inoue, K.; Suzuki, E.; Takahashi, T.; Yamamoto, Y.; Yazawa, R.; Takahashi, Y.; Imai, K.; Miyakawa, K.; Inoue, Y.; Tsuji, D.; et al. 4217C>A polymorphism in carbamoyl-phosphate synthase 1 gene may not associate with hyperammonemia development during valproic acid-based therapy. *Epilepsy Res* **2014**, *108*, 1046-1051, doi:10.1016/j.eplepsyres.2014.04.008.

531. Suzuki, T.; Delgado-Escueta, A.V.; Alonso, M.E.; Morita, R.; Okamura, N.; Sugimoto, Y.; Bai, D.; Medina, M.T.; Bailey, J.N.; Rasmussen, A.; et al. Mutation analyses of genes on 6p12-p11 in patients with juvenile myoclonic epilepsy. *Neuroscience letters* **2006**, *405*, 126-131, doi:10.1016/j.neulet.2006.06.038.

532. Porcelli, S.; Balzarro, B.; Lee, S.J.; Han, C.; Patkar, A.A.; Pae, C.U.; Serretti, A. PDE7B, NMBR and EPM2A Variants and Schizophrenia: A Case-Control and Pharmacogenetics Study.

Neuropsychobiology **2016**, *73*, 160-168, doi:10.1159/000445295.
533. Hsieh, P.C.; Wang, C.C.; Tsai, C.L.; Yeh, Y.M.; Lee, Y.S.; Wu, Y.R. POLG R964C and GBA L444P mutations in familial Parkinson's disease: Case report and literature review. *Brain and behavior* **2019**, *9*, e01281, doi:10.1002/brb3.1281.
534. Klassen, T.L.; Bomben, V.C.; Patel, A.; Drabek, J.; Chen, T.T.; Gu, W.; Zhang, F.; Chapman, K.; Lupski, J.R.; Noebels, J.L.; et al. High-resolution molecular genomic autopsy reveals complex sudden unexpected death in epilepsy risk profile. *Epilepsia* **2014**, *55*, e6-12, doi:10.1111/epi.12489.
535. Vafadari, B.; Salamian, A.; Kaczmarek, L. MMP-9 in translation: from molecule to brain physiology, pathology, and therapy. *Journal of neurochemistry* **2016**, *139 Suppl 2*, 91-114, doi:10.1111/jnc.13415.
536. Hengel, H.; Bosso-Lefèvre, C.; Grady, G.; Szenker-Ravi, E.; Li, H.; Pierce, S.; Lebigot, É.; Tan, T.T.; Eio, M.Y.; Narayanan, G.; et al. Loss-of-function mutations in UDP-Glucose 6-Dehydrogenase cause recessive developmental epileptic encephalopathy. *Nature communications* **2020**, *11*, 595, doi:10.1038/s41467-020-14360-7.
537. Gripp, K.W.; Smithson, S.F.; Scurr, I.J.; Baptista, J.; Majumdar, A.; Pierre, G.; Williams, M.; Henderson, L.B.; Wentzensen, I.M.; McLaughlin, H.; et al. Syndromic disorders caused by gain-of-function variants in KCNH1, KCNK4, and KCNN3-a subgroup of K(+) channelopathies. *European journal of human genetics : EJHG* **2021**, *29*, 1384-1395, doi:10.1038/s41431-021-00818-9.
538. Kortüm, F.; Niceta, M.; Magliozzi, M.; Dumic Kubat, K.; Robertson, S.P.; Moresco, A.; Dentici, M.L.; Baban, A.; Leoni, C.; Onesimo, R.; et al. Cantú syndrome versus Zimmermann-Laband syndrome: Report of nine individuals with ABCC9 variants. *European journal of medical genetics* **2020**, *63*, 103996, doi:10.1016/j.ejmg.2020.103996.
539. Chen, W.; Tan, Y.; Ge, Y.; Chen, Y.; Liu, X. The Effects of Levetiracetam on Cerebrospinal Fluid and Plasma NPY and GAL, and on the Components of Stress Response System, hs-CRP, and S100B Protein in Serum of Patients with Refractory Epilepsy. *Cell biochemistry and biophysics* **2015**, *73*, 489-494, doi:10.1007/s12013-015-0683-8.
540. Carroll, L.S.; Massey, T.H.; Wardle, M.; Peall, K.J. Dentatorubral-pallidoluysian Atrophy: An Update. *Tremor and other hyperkinetic movements (New York, N.Y.)* **2018**, *8*, 577, doi:10.7916/d81n9hst.
541. Rodan, L.H.; Qi, W.; Ducker, G.S.; Demirbas, D.; Laine, R.; Yang, E.; Walker, M.A.; Eichler, F.; Rabinowitz, J.D.; Anselm, I.; et al. 5,10-methenyltetrahydrofolate synthetase deficiency causes a neurometabolic disorder associated with microcephaly, epilepsy, and cerebral hypomyelination. *Molecular genetics and metabolism* **2018**, *125*, 118-126, doi:10.1016/j.ymgme.2018.06.006.
542. Schoch, K.; Meng, L.; Szelinger, S.; Bearden, D.R.; Stray-Pedersen, A.; Busk, O.L.; Stong, N.; Liston, E.; Cohn, R.D.; Scaglia, F.; et al. A Recurrent De Novo Variant in NACC1 Causes a Syndrome Characterized by Infantile Epilepsy, Cataracts, and Profound Developmental Delay. *American journal of human genetics* **2017**, *100*, 343-351, doi:10.1016/j.ajhg.2016.12.013.
543. Fadiel, A.; Song, J.; Tivon, D.; Hamza, A.; Cardozo, T.; Naftolin, F. Phenytoin is an estrogen receptor α-selective modulator that interacts with helix 12. *Reproductive sciences (Thousand Oaks, Calif.)* **2015**, *22*, 146-155, doi:10.1177/1933719114549853.
